# Evolution and Emergence of Enteroviruses through Intra- and Inter-species Recombination: Plasticity and Phenotypic Impact of Modular Genetic Exchanges in the 5’ Untranslated Region

**DOI:** 10.1371/journal.ppat.1005266

**Published:** 2015-11-12

**Authors:** Claire Muslin, Marie-Line Joffret, Isabelle Pelletier, Bruno Blondel, Francis Delpeyroux

**Affiliations:** 1 Institut Pasteur, Biologie des Virus Entériques, Paris, France; 2 INSERM U994, Institut National de Santé et de La Recherche Médicale, Paris, France; 3 Université Paris Diderot, Sorbonne Paris Cité, Cellule Pasteur, Paris, France; University of Michigan, UNITED STATES

## Abstract

Genetic recombination shapes the diversity of RNA viruses, including enteroviruses (EVs), which frequently have mosaic genomes. Pathogenic circulating vaccine-derived poliovirus (cVDPV) genomes consist of mutated vaccine poliovirus (PV) sequences encoding capsid proteins, and sequences encoding nonstructural proteins derived from other species’ C EVs, including certain coxsackieviruses A (CV-A) in particular. Many cVDPV genomes also have an exogenous 5’ untranslated region (5’ UTR). This region is involved in virulence and includes the cloverleaf (CL) and the internal ribosomal entry site, which play major roles in replication and the initiation of translation, respectively. We investigated the plasticity of the PV genome in terms of recombination in the 5’ UTR, by developing an experimental model involving the rescue of a bipartite PV/CV-A cVDPV genome rendered defective by mutations in the CL, following the co-transfection of cells with 5’ UTR RNAs from each of the four human EV species (EV-A to -D). The defective cVDPV was rescued by recombination with 5’ UTR sequences from the four EV species. Homologous and nonhomologous recombinants with large deletions or insertions in three hotspots were isolated, revealing a striking plasticity of the 5’ UTR. By contrast to the recombination of the cVDPV with the 5’ UTR of group II (EV-A and -B), which can decrease viral replication and virulence, recombination with the 5’ UTRs of group I (EV-C and -D) appeared to be evolutionarily neutral or associated with a gain in fitness. This study illustrates how the genomes of positive-strand RNA viruses can evolve into mosaic recombinant genomes through intra- or inter-species modular genetic exchanges, favoring the emergence of new recombinant lineages.

## Author Summary

Recombination shapes viral genomes, including those of the pathogenic circulating vaccine-derived polioviruses (cVDPVs), responsible for poliomyelitis outbreaks. The genomes of cVDPVs consist of sequences from vaccine poliovirus (PV) and other enteroviruses (EVs). We investigated the plasticity of cVDPV genomes and the effects of recombination in the 5’ untranslated region (5’ UTR), which is involved in replication, translation and virulence. We rescued a 5’ UTR-defective recombinant cVDPV genome by cotransfecting cells with 5’ UTR RNAs from human EV species EV-A to -D. Hundreds of recombinants were isolated, revealing striking plasticity in this region, with homologous and nonhomologous recombination sites mostly clustered in three hotspots. Recombination with EV-A and -B affected replication and virulence, whereas recombination with EV-C and -D was either neutral or improved viral fitness. This study illustrates how RNA viruses can acquire mosaic genomes through intra- or inter-species recombination, favoring the emergence of new recombinant strains.

## Introduction

Two driving forces are implicated in the variability of RNA viruses: high mutation rates, which generate a population of related sequences named quasispecies, and genomic RNA recombination [1–3]. By allowing the exchange of genetic information and incorporating viral RNA fragments into new genomic contexts, recombination can eliminate lethal mutations or other genetic alterations [4–9], and increase viral pathogenicity [10–12] and fitness [7, 8, 13, 14]. Genetic recombination has shaped the genetic diversity of RNA viruses and in particular that of enteroviruses (EVs), which often show mosaic genomes [15–22].

EVs belong to the *Picornaviridae* family and are small non-enveloped viruses containing a single positive-strand RNA genome of approximately 7.5 kb in length. This genome consists of two untranslated regions (5’ and 3’ UTR) flanking a unique large open reading frame (ORF). The encoded polyprotein is co- and post-translationally processed by viral proteases to yield four capsid proteins (VP1-4) and non-structural proteins such as the RNA-dependent RNA polymerase 3D that are involved in the viral multiplication and the control of the cellular environment [23]. Human EVs belong to the genus *Enterovirus* and are classified into four species, EV-A, -B, -C and -D [24]. Three species of rhinoviruses (RV-A to -C) have also recently been included in this genus. The three serotypes of poliovirus (PV), the etiological agent of poliomyelitis, belong to the EV-C species.

The global poliomyelitis eradication program conducted by the World Health Organization mainly involves the oral polio vaccine (OPV), which is composed of live attenuated strains of the three PV serotypes, Sabin 1, 2 and 3. These strains are only able to replicate to high titers in the digestive tract, conferring strong systemic and intestinal immunity that limits subsequent PV replication and viral transmission among humans [25]. However, OPV strains are genetically unstable and poor vaccination coverage may allow their circulation between non-immunized humans, leading to genetic drift and the emergence of new pathogenic strains known as circulating vaccine-derived polioviruses (cVDPVs). These cVDPVs threaten the benefits of vaccination campaigns and complicate the eradication strategy [26–28]. Most cVDPVs studied to date have recombinant genomes composed of mutated OPV sequences encoding capsid proteins and sequences encoding non-structural proteins and the 3’ UTR are derived from other EV-Cs [27, 29–32]. The analysis of the cVDPV strains implicated in an outbreak of poliomyelitis in Madagascar in 2002 and another in 2005 revealed that these strains have complex mosaic genomes derived from a highly diverse EV-C viral ecosystem, through intra- and intertypic recombination with different coxsackievirus A (CV-A) types, especially CV-A type 13 (CV-A13) and CV-A17 [21, 29, 33]. Recombination in the non-structural regions was found to influence the phenotypic characteristics of the cVDPVs, including their pathogenicity [12, 34].

Although recombination between PV and other EV-Cs mainly occurs in the non-structural region, many cVDPV genomes have a 5’ UTR acquired by intertypic recombination [21, 35–38]. The EV 5’ UTR is about 740 nucleotides (nt) in length and contains seven highly conserved stem-loop domains (I to VII) forming two functional units. Domain I forms a cloverleaf (CL) structure required for initiating both negative- and positive-strand RNA synthesis [39–42]. Domains II to VI (dII to dVI) contain the internal ribosome entry site (IRES) that initiates cap-independent translation by interacting with canonical and noncanonical cellular translation factors to recruit ribosomes [43, 44]. The CL and IRES elements are separated by a pyrimidine-rich sequence of about 40 nt, named spacer 1, implicated in both the translation and replication of the viral genome. The IRES is linked to the initiation AUG codon by dVII and a poorly structured sequence of about 100 nt, named spacer 2, that is dispensable for viability [45–47].

The human EV 5’ UTR sequences cluster into two different major phylogenetic groups: group I, which is comprised of EV-C and EV-D, and group II, which is formed by EV-A and EV-B [17]. Despite the extent of sequence divergence, the 5’ UTR of all EVs have a similar structure that is essential for its function. Indeed, the construction of some 5’ UTR chimeric genomes leads to viruses with viable progeny, suggesting that 5’ UTR elements are somewhat interchangeable between human EVs [48–53]. However, the rules determining the exchangeability between 5’ UTRs from different EVs have not been extensively defined.

The characterization of chimeric viruses made *in vitro* has also revealed how the 5’ UTR contributes to cell tropism, host range and virulence [48, 52–56]. In addition, the three Sabin vaccine strains contain strong attenuation determinants in the dV of the 5’ UTR of the viral genome, which further indicates that the 5’ UTR influences the pathogenicity of PVs [57–59]. Thus, we hypothesized that the natural acquisition of a new 5’ UTR by a cVDPV affects its phenotypic characteristics and may lead to the emergence of new virulent strains.

Two possible nonexclusive mechanisms of recombination exist for RNA viruses: a replicative copy-choice mechanism, first described for PV, which involves template-switching of the viral polymerase during negative-strand RNA synthesis [60], and a nonreplicative process, which involves breakage of the RNA strands and subsequent ligation reaction, probably mediated by cellular factors [5, 8, 61, 62]. Although both mechanisms can lead to homologous and nonhomologous recombinants, almost all natural cVDPVs reported to date are homologous recombinants. We previously developed a recombination system to study the molecular mechanisms involved in intertypic genetic exchanges between the non-structural regions of PV and non-PV EV-Cs. This system rescues defective type 2 PV RNA genomes with a short deletion at the 3’ end by co-transfecting cells with defective or infectious CV-A17 RNAs [6].

In the present study, we used this *in vitro* system to investigate further the plasticity of the PV genome concerning recombination in the 5’ UTR. The recombination system was adapted to rescue a defective cVDPV genome following the co-transfection of cells with 5’ UTR RNAs from each of the four human EV species. Analysis of the rescued recombinants showed that the cVDPV 5’UTR is very permissive to genetic exchanges. Homologous and nonhomologous recombination sites mainly located in three recombination hotspots in the 5’ UTR were found. We also examined how the exchanges influenced the phenotype of the recombinants. Our results suggest that the acquisition by cVDPVs of 5’ UTRs from certain CV-A favors the emergence of new recombinant lineages.

## Results

### Development of a system targeting recombination to the 5’ UTR

**Fig 1:**
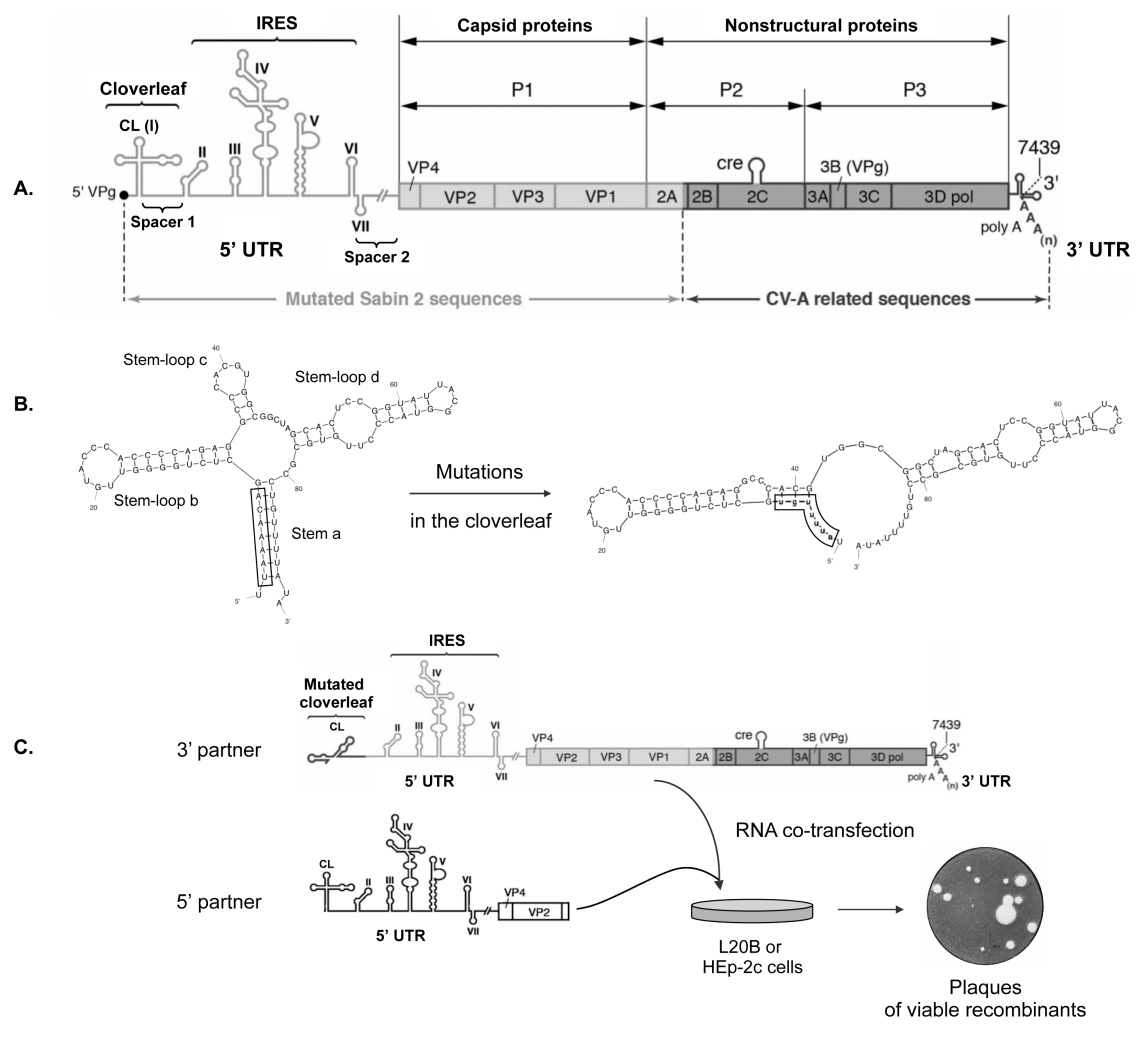
Experimental model of recombination in the 5’ untranslated region of enteroviruses. (A) Schematic representation of the genome of MAD4 cVDPV. The poly-adenylated single positive-strand RNA genome is covalently linked to the viral protein VPg (also named 3B) at the 5’ terminus. The unique large open-reading frame is flanked by two untranslated regions (5’ and 3’ UTRs). The 5’ UTR (nt 1 to 747) is magnified to indicate the seven stem-loop structures (I to VII) forming two functional units, the cloverleaf (CL: I) and the internal ribosome entry site (IRES: II-VI). Genomic regions encoding viral proteins VP4 to 3Dpol are indicated. The MAD4 genome is a PV/EV-C recombinant between mutated Sabin 2 sequences (light shading) and non-vaccine sequences derived from coxsackieviruses A (CV-A) (dark shading). (B) Substitutions in the CL leading to a noninfectious MAD4 genome. The native CL structure can be subdivided into four domains: stem a and stem-loops b to d. Mutations (lowercase) introduced into the CL of MAD4 disrupt stem a and create new predicted base pairing interactions. Secondary structure predictions were generated with mfold, version 3.6 [63]. (C) Rescue of the defective MAD4 genomic RNA (3’ partner) by co-transfection with 5’ UTR sequences from EVs (5’ partners). 5’ partners included the complete 5’ UTR followed by sequences encoding VP4, VP2 and part of VP3 from eight different EV strains belonging to the four human EV species (see Fig 2). Human HEp-2c and murine L20B cells were co-transfected with each RNA partner pair and then incubated in semisolid medium until plaques appeared, indicating the generation of viable recombinants.

To explore recombination and the compatibility between PV, in particular cVDPVs, and the different EV species in the 5’ UTR, we developed an *in vitro* system based on the co-transfection of cells with two noninfectious RNA fragments called 5’ and 3’ partners (Fig 1).

**Fig 2:**
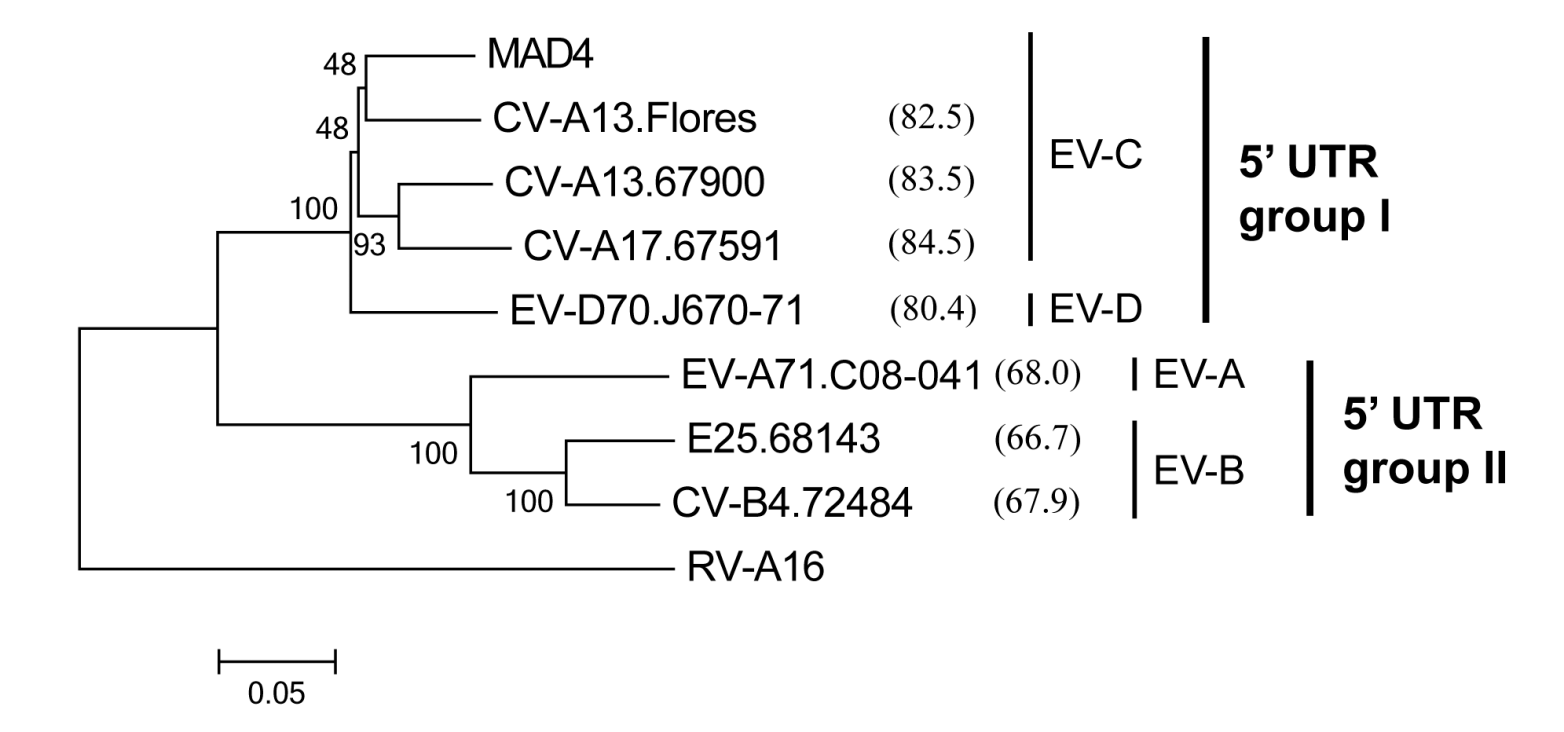
Phylogenetic relationships between the 5’ UTR sequences of the eight 5’ partners. This neighbor-joining tree was constructed with MEGA, version 6.06 [65] using aligned 5’ UTR nt sequences. The reliability of tree topology was estimated using 1000 bootstrap replicates. The nt sequence of rhinovirus A16 (RV-A16) was used as an outgroup. The name of each isolate includes the type of the isolate followed by the laboratory number or name for the prototype strains. For each 5’ partner, the percentage similarity with MAD4 is shown in brackets. The enterovirus species (EV-A to -D) and the 5’ UTR group I and II are indicated.

These recombination partners were designed to create a pair of defective genomes that would generate viable viruses only if a recombination event occurred in the 5’ UTR or in the N-terminal part of the ORF encoding capsid proteins. The 3’ RNA recombination partner was constructed from the genome of the MAD4 strain, a Madagascan Sabin 2-derived cVDPV implicated in an outbreak of poliomyelitis in 2002 [29, 64]. This strain was found to be a PV/CV-A bipartite recombinant with the 5’ half of its genome derived from mutated Sabin 2 sequences, and the 3’ half encoding the non-structural viral proteins related to those of co-circulating EV-C, in particular CV-A17 (Fig 1A). The 3’ RNA partner was made from the whole MAD4 genome in which nt 2 to 8 were replaced by their complementary sequence (Fig 1B). This substitution disrupts the first stem structure of the CL, making the viral RNA noninfectious [39]. The 5’ recombination partners were designed to provide the entire 5’ UTR and the beginning of the ORF including the VP4 and VP2 regions and part of the VP3 region, therefore acting as donors of a functional CL. Eight 5’ partners were constructed from strains belonging to the four human EV species: EV-A71 (EV-A), CV-B4 and echovirus 25 (E25; EV-B), MAD4, CV-A17 and two CV-A13 (EV-C), and EV-D70 (EV-D) (see S1 File for aligned nucleotide sequences). The 5’ UTR sequences of the 5’ partners belong to either group I or II (Fig 2 and S1 Fig).

The MAD4 3’ RNA partner was mixed with each 5’ RNA partner and transfected into human epithelial HEp-2c cells which were subsequently grown on semisolid medium to allow plaque formation (Fig 1C). Murine L20B cells expressing the human PV receptor, CD155, were also transfected [66]. As expected, HEp-2c and L20B cells transfected with the 3’ RNA partner or the 5’ RNA partners alone produced no plaques. However, in both cell lines, co-transfection with each partner pair led to the formation of plaques corresponding to infectious recombinant viruses after 2 to 7 days.

Recombination efficiency depended on the cell line (Table 1). Following co-transfection, L20B cells yielded 2 to 6 times more plaques than HEp-2c cells and the plaques developed 1 to 4 days earlier. The sequence identity shared by the two partners did not influence the number of generated recombinants. Nevertheless, except for one partner pair (MAD4 and CV-B4.72484), recombination appeared to be more efficient when the 3’ and 5’ partners belonged to the same 5’ UTR group than when they belonged to different groups.

**Table 1:** Recombination efficiency following the co-transfection of L20B and HEp-2c cells with the MAD4 3’ RNA partner and each 5’ RNA partner.

		Cell line			
		L20B		HEp-2c	
5’ UTR group^*a*^	5’ partner	Days after transfection^*b*^	PFU / μg of 5’ partner RNA^*c*^	Days after transfection	PFU / μg of 5’ partner RNA
I	MAD4	2	404 ± 4	4	160 ± 8
	CV-A13.Flores	2	366 ± 46	5	150 ± 18
	CV-A13.67900	2	340 ± 60	4	154 ± 10
	CV-A17.67591	2	602 ± 14	3	276 ± 4
	EV-D70.J670-71	2	442 ± 18	5	78 ± 14
II	EV-A71.C08-041	3	106 ± 12	7	36 ± 2
	CV-B4.72484	2	402 ± 6	5	96 ± 12
	E25.68143	3	134 ± 30	5	35 ± 5

^*a*^ The 5’ UTR group of the 5’ partner is indicated.

^*b*^ The period following co-transfection was optimized before staining and counting PFUs, depending on the RNA partner pairs.

^*c*^ The average numbers of plaques ± the standard deviation of two experiments is given. The same amount (in μg) of 5’ and 3’ partner was co-transfected.

Thus, the noninfectious mutated MAD4 3’ partner could be rescued by recombination with all the 5’ partners, even those originating from different 5’ UTR group or species.

### Analysis of recombination sites

We determined the features of the recombinant genomes generated following the co-transfection of the MAD4 3’ partner with each of the seven non PV 5’ partners. Around 30 recovered recombinant viruses per partner pair and cell line were isolated by picking plaques. About 1000 nt at the 5’ end of the viral RNA, the region in which recombination occurs, were sequenced. Recombination sites were determined for 426 viruses, by comparing their genomic sequences with those of the parental partners. Sequence alignments revealed both homologous recombinant genomes and nonhomologous ones showing insertions (mostly duplications) or deletions at the recombination site. Examples of homologous and nonhomologous recombination sites are shown in S2 Fig.

**Fig 3:**
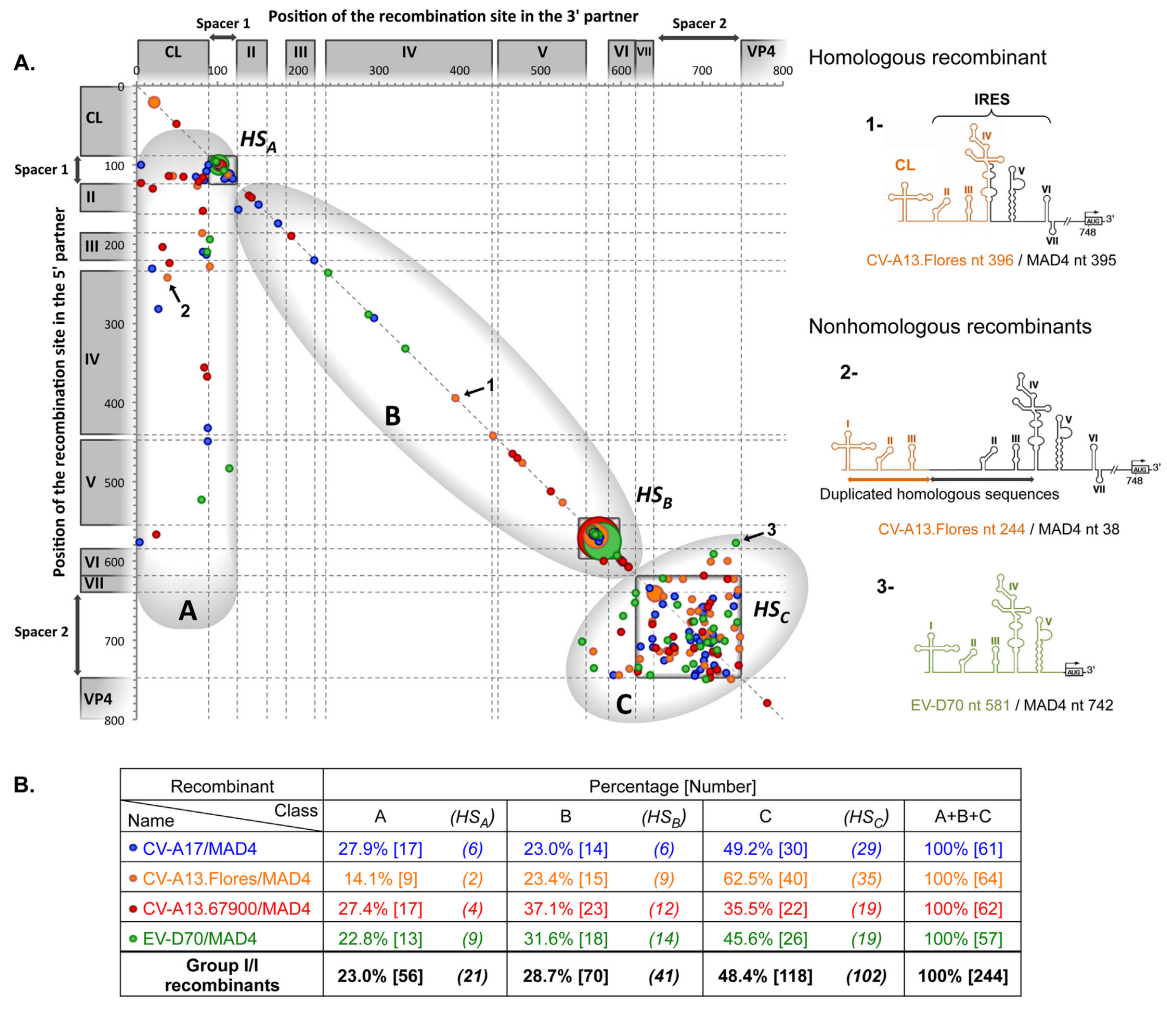
Location of recombination sites in group I/I recombinants. (A) Genomes of recombinant viruses resulting from the co-transfection of the 3’ partner MAD4 with 5’ partners from group I 5’ UTRs (S2 File). Each recombinant is plotted on the graph according to the position of its recombination site in the 3’ partner sequence (on the x-axis) and in the 5’ partner sequence (on the y-axis). To facilitate the comparison between the different 5’ partners, the location of 5’ UTR domains and crossover sites are indicated, following alignment of all considered 5’ UTR sequences, numbered according to the MAD4 sequence. Homologous recombinants are located on the diagonal (identity line), whereas recombinants displaying insertions or deletions are below or above this line, respectively. Three examples numbered from 1 to 3 are shown (arrows). The corresponding 5’ UTR structures are drawn on the right side. Recombinants 1, 2 and 3 are examples of a homologous and two nonhomologous recombinants with an insertion and a deletion, respectively. The three recombinant classes identified on the graph, A, B and C, are circled, and the hotspot (HS) within each class, HS_A_, HS_B_ and HS_C_, are framed. The size of the dot is proportional to the number of recombinants having the same recombination site coordinates (from 1 to 9). The color code indicates the respective recombinant type (see table below). (B) The table shows the percentage and number of recombinants within each class. The number of recombinants with recombination sites located in hotspots are indicated in brackets.

Viruses generated following recombination between partners from the same or from different 5’ UTR groups were designated as group I/I or group II/I recombinants, respectively. The distribution of group I/I recombination sites obtained in both L20B and HEp-2c cells is plotted in Fig 3A. The recombination region stretched from nt 21 (CL) in the 5’ partner to nt 780 (VP4) in the 3’ partner (MAD4) and no recombination site was found further downstream. Most recombinants could be divided into three distinct classes, A, B and C.

Class A recombinants had the CL from the 5’ partner and the IRES from MAD4. A few short deletions of 1 or 2 nt (17.9%) and a high percentage of insertions (60.7%) could be seen. These insertions were 10 to 573 nt long, leading in more than half cases to the duplication of one or more IRES domain(s). Thus, the sequence linking the functional CL, from the 5’ partner, to the complete IRES, from the 3’ partner, appeared to be very tolerant to large insertions. Given that 37.5% of class A recombinant genomes contained a recombination site located in the spacer 1 of both partners, this sequence can be considered to be a recombination “hotspot” region (HS_A_).

Class B genomes had a recombinant IRES, with some domain(s) originating from the 5’ partner and the remaining ones from MAD4. Thus, IRES domains of the 5’ partner were able to replace functionally those of MAD4. Recombination sites were even found within a stem-loop, leading to functional recombinant domains. Interestingly, 77.1% of class B recombinants were homologous, suggesting that the length and secondary structure of the IRES are highly constrained. The other recombinants contained short insertions or deletions (-4 to +23 nt), which were located in some cases in a stem-loop structure. The 25–26 nt long region between dV and dVI appeared to be an important recombination hotspot (HS_B_), because it contained 58.6% of crossover junctions in class B in only 5% of the total nt length of the IRES (Fig 3).

Recombinants of the major class (C) contained a recombination site located within the dVII and spacer 2 region, at the 3’ extremity of the 5’ UTR, resulting from the exchange of both the CL and IRES. Overall, 35.5% of the CV-A13.67900/MAD4 and 62.5% of the CV-A13.Flores/MAD4 recombinants displayed this feature (Fig 3B). In total, 48.4% of group I/I recombinants originated from the recombination of both partners in the dVII and spacer 2 region; therefore, this region can be considered to be a recombination hotspot (HS_C_). Only six of the 118 class C viruses were homologous recombinants. The others contained deletions (44.9%) or insertions (50.0%) ranging from -164 to +170 nt, which could lead to the complete deletion or duplication of the dVII and spacer 2 region. For some genomes containing the entire CL and IRES of the 5’ partner, the recombination breakpoint was located within dVI of the MAD4 IRES or even between dV and dVI leading to a duplication of dVI. More surprisingly, four recombinant genomes lacked part of or the entire dVI.

**Fig 4:**
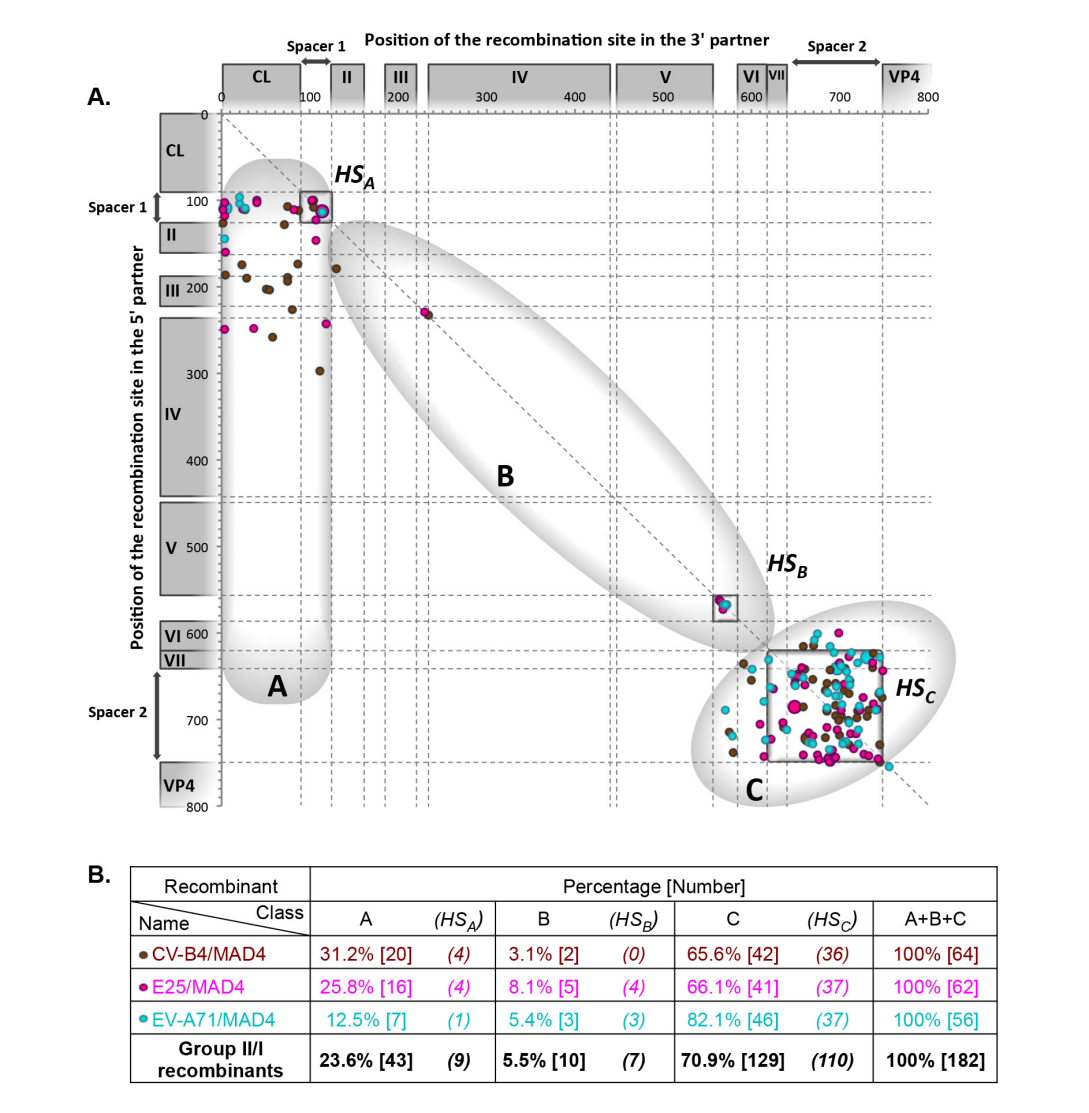
Location of recombination sites in group II/I recombinants. Genomes of recombinant viruses resulting from the co-transfection of the 3’ partner MAD4 with 5’ partners from group II 5’ UTRs (S3 File). For details, see the legend of Fig 3.

Group II/I recombinants from L20B and HEp-2c cells, the genomic features of which are shown in Fig 4, also segregated as group I/I recombinants into three classes, A, B and C. The proportion of class A recombinants was similar in both groups (23.6% in group II/I and 23.0% in group I/I), but the proportions of class B and C recombinants were different between the two groups. Only 5.5% of group II/I recombinants had a recombinant IRES (class B recombinants), versus 28.7% for group I/I recombinants. Class C recombinants, originating from the exchange of the entire 5’ UTR, accounted for 70.9% of recombinants in group II/I versus only 48.4% of group I/I. Thus, the structural and functional features of group I and II 5’ UTRs influence recombination.

The high diversity of viable homologous and nonhomologous recombinant genomes generated with this *in vitro* system reveals that the 5’ UTR of EVs is very plastic. However, when we compared the features of recombinants obtained in HEp-2c and in L20B cells, this diversity appeared to be influenced by the cell line (S3 and S4 Figs). For group I/I recombinants, although the proportions of class A recombinants obtained in HEp-2c and L20B cells were similar (23.4% and 22.5%, respectively), the cell line used affected the proportion of class B (44.4% in HEp-2c and 12.5% in L20B cells) and class C (32.3% in HEp-2c and 65.0% in L20B cells) recombinants (S3 Fig). For group II/I recombinants, the percentages of both class A and B recombinants were lower in L20B cells (32.6% and 10.1%, respectively) than in HEp-2c cells (15.1% and 1.1%, respectively) whereas the proportion of class C recombinants was higher (83.9% in L20B and 57.3% in HEp-2c cells) (S4 Fig). Thus, the distribution of the recombinants in each of the three classes appeared to depend on the cell line. We investigated whether these differences between HEp-2c and L20B cells involved selection events, by comparing the growth, in these two cell lines, of 18 recombinants isolated from HEp-2c cells and six isolated from L20B cells, from each of the three recombinant classes (S4 File). In particular, nine of the recombinants isolated from HEp-2c cells belonged to class B (with a recombinant IRES), the class most rarely observed in L20B cells. No significant difference in viral growth between the two cell lines was observed for the six recombinants isolated from L20B cells. By contrast, five of the 18 recombinants isolated from HEp-2c cells, including three with a recombinant IRES (class B), grew markedly better in these cells than in L20B cells. These findings suggest that there may be factors restricting the multiplication and, thus, the emergence of certain recombinants in mouse cells but not in human cells. However, the specific restriction of the class B recombinants in L20B cells remains to be elucidated. Other factors specific of HEp-2c and L20B cells, possibly acting on recombination processes, may be involved in this restriction.

### Genetic stability of nonhomologous recombinant viruses during cell passaging

Most rescued recombinants (70% of group I/I and 94% of group II/I) were nonhomologous recombinants containing insertions or deletions. Given that almost all natural cVDPVs reported to date are homologous recombinants, we hypothesized that nonhomologous genomes are unstable, and lead to genuine genome-length recombinants through genomic rearrangement processes, as shown previously [6, 7].

We selected eight recombinant viruses obtained in HEp-2c cells, and evaluate their stability after serial passages in these cells at low MOI (0.1 TCID_50_ per cell) (Table 2). Both CV-A13.67900/MAD4 recombinant genomes that displayed insertions (A.54 and A.64) had lost their additional sequences at passage 7. By contrast, both CV-B4/MAD4 recombinants with insertions (A.37 and B.49) were stable until passage 12, and cells at this passage contained traces of homologous sequences and the parental one. At passage 17, homologous recombinants had completely or largely replaced the nonhomologous ones. In all cases, the homologous junctions mapped within the limits of the inserted sequences found in the original recombinants. In the homologous recombinants CV-A13.67900/MAD4 B.69 and B.55, mutations at the recombination site modified the stem loop structure of the IRES domain dII and dVI, respectively (S5 Fig). Both recombinants were stable until the last passage 17, although a C to T substitution at nt 160 of B.69 appeared at passage 7, which partially restored the dII stem (S5 Fig). The two CV-B4/MAD4 nonhomologous recombinants with deletions (C.39 and C.59) also maintained their genomic structure upon passaging. Thus, although nonhomologous recombinants with deletions were genetically stable during passaging, those with insertions evolved more or less rapidly into homologous recombinants.

**Table 2:** Stability of selected CV-A13.67900/MAD4 and CV-B4/MAD4 recombinants upon serial passaging.

				Status at passage no.^*e*^:		
Recombinant type and name^*a*^		Recombination site location^*b*^	Recombination site features^*d*^	P7	P12	P17
(Group I/I) CV-A13.67900/MAD4	A.64	CV-A13 nt 228 (dIII-dIV) / MAD4 nt 41 (CL)	+ 184 nt [dII + dIII]	Homologous		
	A.54	CV-A13 nt 572 (dV-dVI) / MAD4 nt 24 (CL)	+ 544 nt [dII to dV]	Homologous		
	B.69	CV-A13 nt 145 (dII) / AATT / MAD4 nt 146 (dII)^c^	H	Parental	Parental	Parental
	B.55	CV-A13 nt 605 (dVI) / AT / MAD4 nt 604 (dVI)^c^	H	Parental	Parental	Parental
(Group II/I) CV-B4/MAD4	B.49	CV-B4 nt 183 (dII-dIII) / MAD4 nt 130 (dII)	+ 50 nt	Parental	Mixture	Homologous
	A.37	CV-B4 nt 209 (dIII) / MAD4 nt 51 (CL)	+ 153 nt [dII]	Parental	Mixture	Mixture
	C.39	CV-B4 nt 621 (dVI) / MAD4 nt 659 (spacer 2)	- 42 nt	Parental	Parental	Parental
	C.59	CV-B4 nt 628 (dVII) / MAD4 nt 738 (spacer 2)	- 113 nt	Parental	Parental	Parental

^*a*^ The recombinant group is indicated in brackets. The name of a recombinant includes the type (e.g. CV-A13.67900/MAD4) and class (A) followed by the laboratory number (64).

^*b*^ The 5’ UTR domain in which the recombination site is located is indicated in brackets. For example, (dIII-dIV) designates the linker sequence between domains dIII and dIV.

^*c*^ In these particular homologous recombinants (which contain neither an insertion nor a deletion) a few partner nt were replaced with different nt at the junction (sequence indicated).

^*d*^ The length of insertions (+) and deletions (-) is given. For insertions, the duplicated IRES domains are indicated in square brackets. Homologous recombinant sites are shown (H).

^*e*^ Number of passages. The status of the resulting recombinant is indicated following passaging. Homologous: loss of insertion. Parental: recombination site unchanged, conservation of the parental virus sequences. Mixture: mixture of parental and homologous recombinants.

### Fitness of nonhomologous recombinant viruses

**Fig 5:**
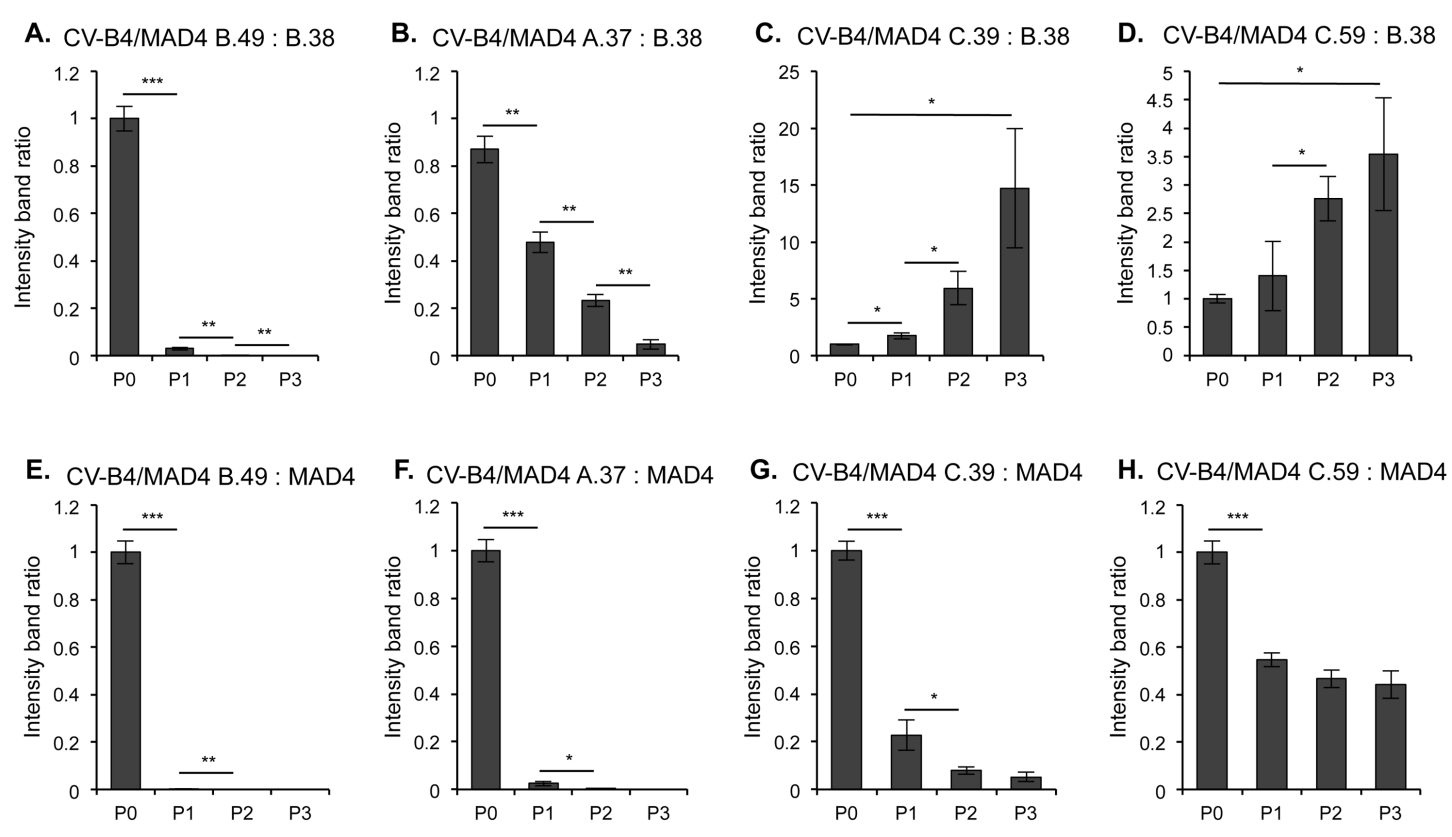
Competition assays comparing the relative fitness of homologous and nonhomologous recombinants. Each of the four nonhomologous selected CV-B4/MAD4 recombinants presented in Table 2 were competed against the homologous recombinant CV-B4/MAD4 B.38, the recombination site of which is located in the spacer between IRES domains dIII and dIV (CV-B4 nt 240 / MAD4 nt 234) (A to D), and against the parental cVDPV MAD4 (E to H). Viruses were mixed at a 1:1 ratio and HEp-2c cells were inoculated in triplicate at an MOI of 0.01 TCID50/cell for three passages. At each passage, the progeny RNA was amplified by RT-PCR and the relative amount of each competitor was evaluated by measuring the intensity of the bands on agarose gel electrophoresis (ImageJ 1.47 software, NIH) corresponding either to the nonhomologous recombinant or to its homologous competitor. The ratio of band intensity for the nonhomologous recombinant versus the homologous competitor is shown for each well for the three passages (S6 Fig). An increase in this ratio indicates that the nonhomologous recombinant is more fit than the homologous one. Error bars indicate the standard deviation (Student’s t-test, n = 3; *P<0.*05*, **P<0.*01*, ***P<0.*001*).

CV-B4/MAD4 nonhomologous recombinant genomes were unexpectedly stable during serial passaging. However, we reasoned that when hosts are co-infected with a nonhomologous and a parental or homologous strain, a growth disadvantage may lead to the counterselection of the nonhomologous strain. We therefore assessed the relative fitness of these strains in competition with a CV-B4/MAD4 homologous recombinant (CV-B4/MAD4 B.38) or the parental strain MAD4 during three successive passages. In a competition assay with the homologous B.38 strain, both of the CV-B4/MAD4 recombinants with insertions (B.49 and A.37) showed a fitness disadvantage (Fig 5A and 5B). Surprisingly, both the C.39 and C.59 strains, which contain deletions, were fitter than the homologous B.38 strain (Fig 5C and 5D). Nevertheless, all CV-B4/MAD4 nonhomologous recombinants were less fit than the parental MAD4 strain (Fig 5E to 5H). In particular, B.49 and A.37 could not be detected after passage 1. Furthermore, from the experiments in which CV-B4/MAD4 B.38 and MAD4 competed against the same strain C.39 (or C.59), we could conclude that the homologous CV-B4/MAD4 B.38 was less fit than the parental MAD4.

Thus, nonhomologous recombinants, in particular those with inserted sequences, were less fit than the parental MAD4. This may prevent them from multiplying when competing with the parental virus or with homologous recombinants in the infected cells or hosts. We then focused on the characteristics of homologous recombinants obtained with our model.

### Impact of the origin of the 5' UTR on the phenotype of homologous recombinants

We next examined how the acquisition of a 5’ UTR originating from a non PV EV (NPEV) influences the phenotypic characteristics of the cVDPV. For each of the four EV species, we selected three homologous or almost homologous recombinants mostly obtained in HEp-2c cells with recombination sites located at the three previously identified hotspots. If no homologous recombinant had been isolated, the nonhomologous recombinant with the smallest insertion or deletion was selected (Table 3 and S5 File).

**Table 3:** Location of recombination site in selected recombinants.

Recombinant group	5’ partner species	Recombinant name^*a*^		Recombination region	5’ partner nt position^*b*^	MAD4 nt position^*b*^	Insertion/ deletion length (nt)^*c*^
Group I/I	EV-C	CV-A17/MAD4	A.37	Spacer 1	115	114	H
			B.57	dV-dVI	568	565	H
			C.51	Spacer 2	689	684	+7
	EV-D	EV-D70/MAD4	A.49	Spacer 1	100	101	H
			B.48	dV-dVI	579	576	H
			C.35	Spacer 2	704	706	- 2
Group II/I	EV-B	E25/MAD4	A.54	Spacer 1	100	102	- 1
			B.58	dV-dVI	572	568	H
			C.45	Spacer 2	734	733	+10
	EV-A	EV-A71/MAD4	A.17	Spacer 1	117	114	+1
			B.55	dV-dVI	571	572	- 4
			C.42	Spacer 2	697	685	+3

^*a*^ Recombinants are named according to their type, class and laboratory number as described in Table 2.

^*b*^ The first number refers to the nt sequence of the indicated 5’ partner and the second to that of MAD4.

^*c*^ Homologous recombination sites (H) display neither an insertion nor a deletion according to aligned parental sequences. The insertion (+) or deletion (-) of nt in nonhomologous sites is indicated.

**Fig 6:**
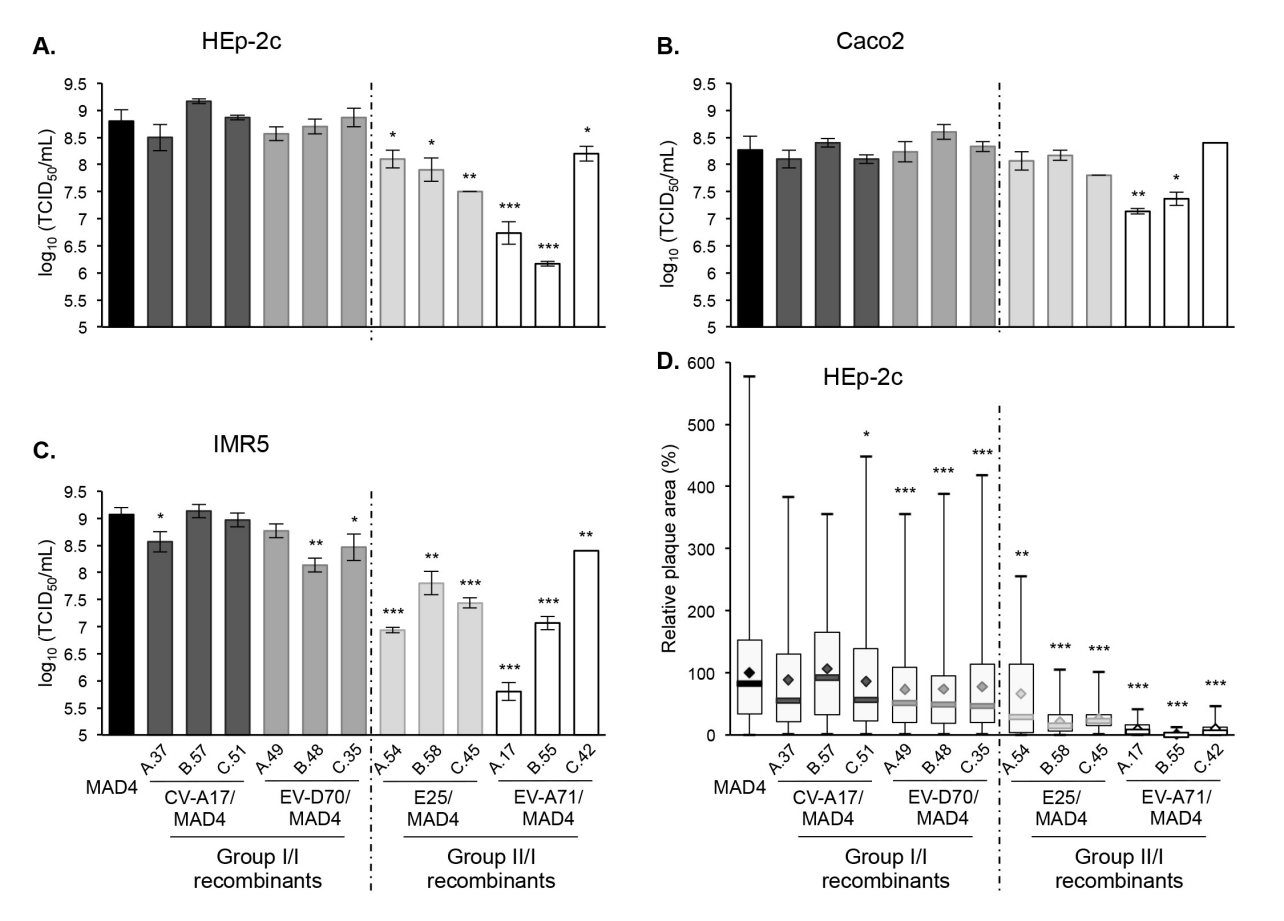
Growth and plaque size of selected recombinant viruses. HEp-2c (A), Caco2 (B) and IMR5 (C) cells were infected with MAD4 and the 12 selected recombinants at an MOI of 10 TCID_50_/cell for 5 hours. Each point shows the log10 of the mean total virus titer from three independent experiments. Error bars indicate the standard deviation. *P<0.05, **P<0.01, ***P<0.001 in a Student’s t-test comparing each selected recombinant virus with MAD4 (n = 3). D. Plaque assays were performed on HEp-2c cell monolayers (two or three experiments). The relative area of plaques was calculated, with the average value of MAD4 plaque area set at 100%. Box plots show median values (thick bars) as well as maximum and minimum values. Averages are indicated (◊). (Student’s t-test, n = 39–578; *P<0.05, **P<0.01, ***P<0.001).

We compared the growth phenotypes of the selected recombinant viruses with that of the parental MAD4 strain in human HEp-2c, intestinal Caco2 and neuronal IMR5 cell lines. In general, group I/I recombinants appeared to replicate as well as MAD4 in the three cell types, although CV-A17/MAD4 A.37, EV-D70/MAD4 B.48 and C.35 displayed a small replication deficit in IMR5 cells, which did not exceed one log_10_ (Fig 6). Thus, the exchange of 5’ UTR within the same 5’ UTR group did not seem to significantly affect viral growth.

By contrast, the group II/I recombinants were all replication deficient in HEp-2c and IMR5 cells compared to MAD4 (Fig 6A and 6C). Furthermore, the difference depended on the recombinant and the cell line. Indeed, in most cases, the replication deficit of group II/I recombinants was more pronounced in IMR5 than in HEp-2c cells. In Caco2 cells, only two recombinant viruses (EV-A71/MAD4 A.17 and B.55) were replication deficient (Fig 6B).

We then assessed whether the growth phenotypes of recombinants were correlated to their capacity to spread and to form plaques in a semi-solid medium (Fig 6D). Among group I/I recombinants, only the three CV-A17/MAD4 recombinants formed plaques as large as those formed by MAD4 in HEp-2c cell monolayers. Plaques formed by the three EV-D70/MAD4s were slightly smaller. Most group II/I E25/MAD4s and EV-A71/MAD4s recombinants formed very small plaques, in particular EV-A71/MAD4 C.42, which nevertheless yielded the highest titer among group II/I recombinants in the three cell lines. Thus, the transfer of the complete 5’ UTR of EV-A71 to MAD4 impairs cell-to-cell viral spread or cytopathogenic effect.

These experiments, involving a few recombinants, suggest that recombination of MAD4 with a group II but not a group I 5’UTR affects in most cases viral replication and/or propagation. Thus, the origin of the 5’ UTR has an impact on the multiplication of recombinants.

### Neurovirulence of homologous recombinant viruses in mice transgenic for the poliovirus receptor

**Fig 7:**
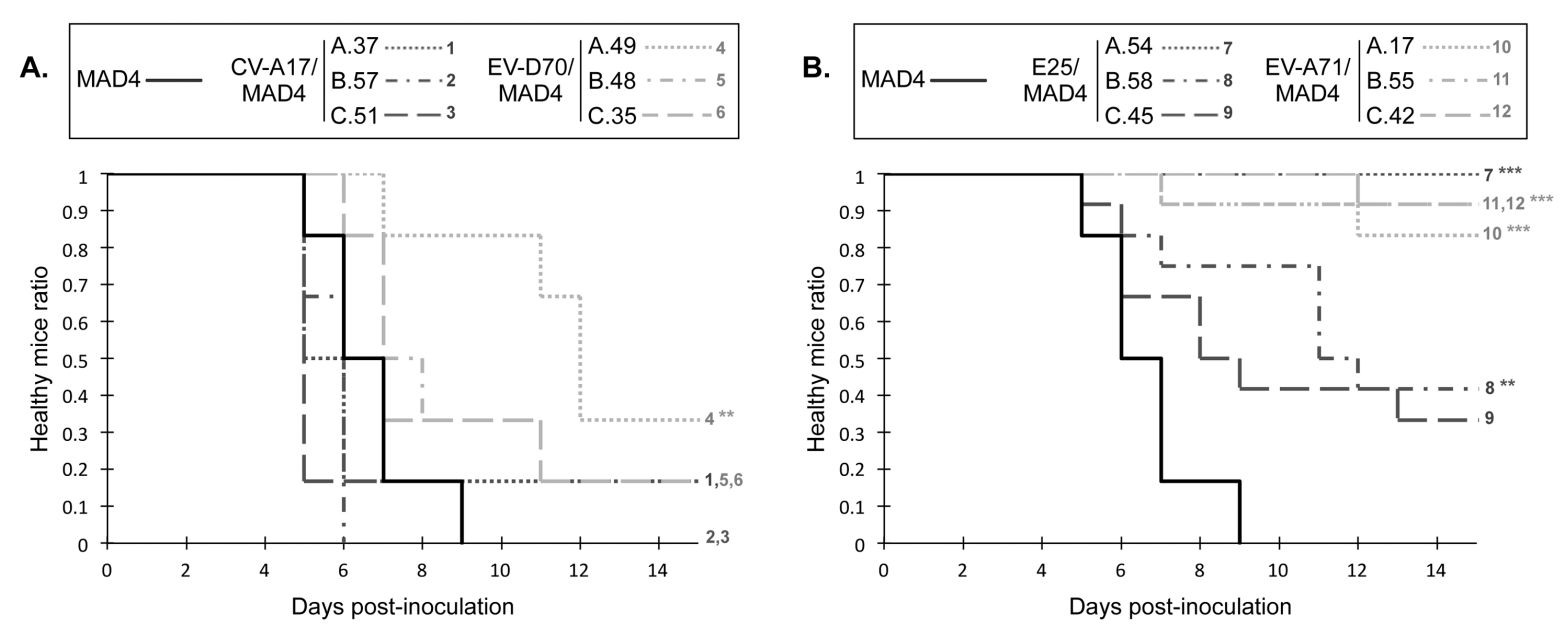
Neurovirulence of MAD4 and selected recombinant viruses in PVR-Tg mice. PVR-Tg21 mice expressing the human PV receptor were inoculated intracerebrally with 10^5^ TCID50 of virus (six mice per virus). Animals were checked daily for 21 days post-inoculation for paralysis or death. The number of healthy mice following inoculation with group I/I recombinants (A) or group II/I recombinants (B) relative to that following inoculation with the parental cVDPV MAD4 is shown. Survival curves are numbered to indicate overlap. No additional mice suffered paralysis or died after day 15 post-inoculation. *P<0.05, **P<0.01, ***P<0.001 in Log Rank tests comparing each selected recombinant virus with MAD4 (S6 File).

We then evaluated the pathogenicity of recombinants in transgenic homozygous PVR-Tg21 mice, which constitutively express the human PV cellular receptor CD155 [67, 68]. The inoculation of these mice with pathogenic PVs through the parenteral or intranasal (mucosal) route produces symptoms of paralysis similar to those of human poliomyelitis. Following intracerebral inoculation, most recombinants were pathogenic in mice and the six group I/I recombinants showed potent neurovirulence that was comparable to that of the virulent MAD4 (Fig 7A). The results obtained with group II/I recombinants were more heterogeneous (Fig 7B). The three EV-A71/MAD4s were either weakly pathogenic or not pathogenic in PVR-Tg21 mice at the tested dose. Interestingly, E25/MAD4 A.54, which contains the CL from E25 in the background of the MAD4 genome, also showed an attenuated phenotype, whereas E25/MAD4 B.58 and C.45 exhibited moderate neurovirulence.

These experiments, involving a few recombinants, suggest that the origin of the 5' UTR also affects the pathogenicity of recombinants in this transgenic mouse model. Interestingly, most group I/I recombinants, including the CV-A17/MAD4 recombinants, were as neurovirulent as the parental cVDPV MAD4.

### Competition assays between 5’ UTR homologous recombinants and the parental MAD4

*In vitro* and *in vivo* studies indicated that group I/I recombinants displayed phenotypic characteristics similar to those of the parental strain MAD4. Given that many isolated cVDPVs were natural recombinants resulting from the exchange of the complete 5’ UTR with a co-circulating CV-A [21, 37], we hypothesized that recombination in the 5’ UTR provides a phenotypic advantage favoring the emergence of new pathogenic strains. We thus compared the fitness of the six group I/I recombinants with that of MAD4 by performing competition assays in HEp-2c cells for three successive passages. At each passage, the relative proportion of the *in vitro* recombinant compared to the parental strain MAD4 was determined by real-time RT-PCR.

**Fig 8:**
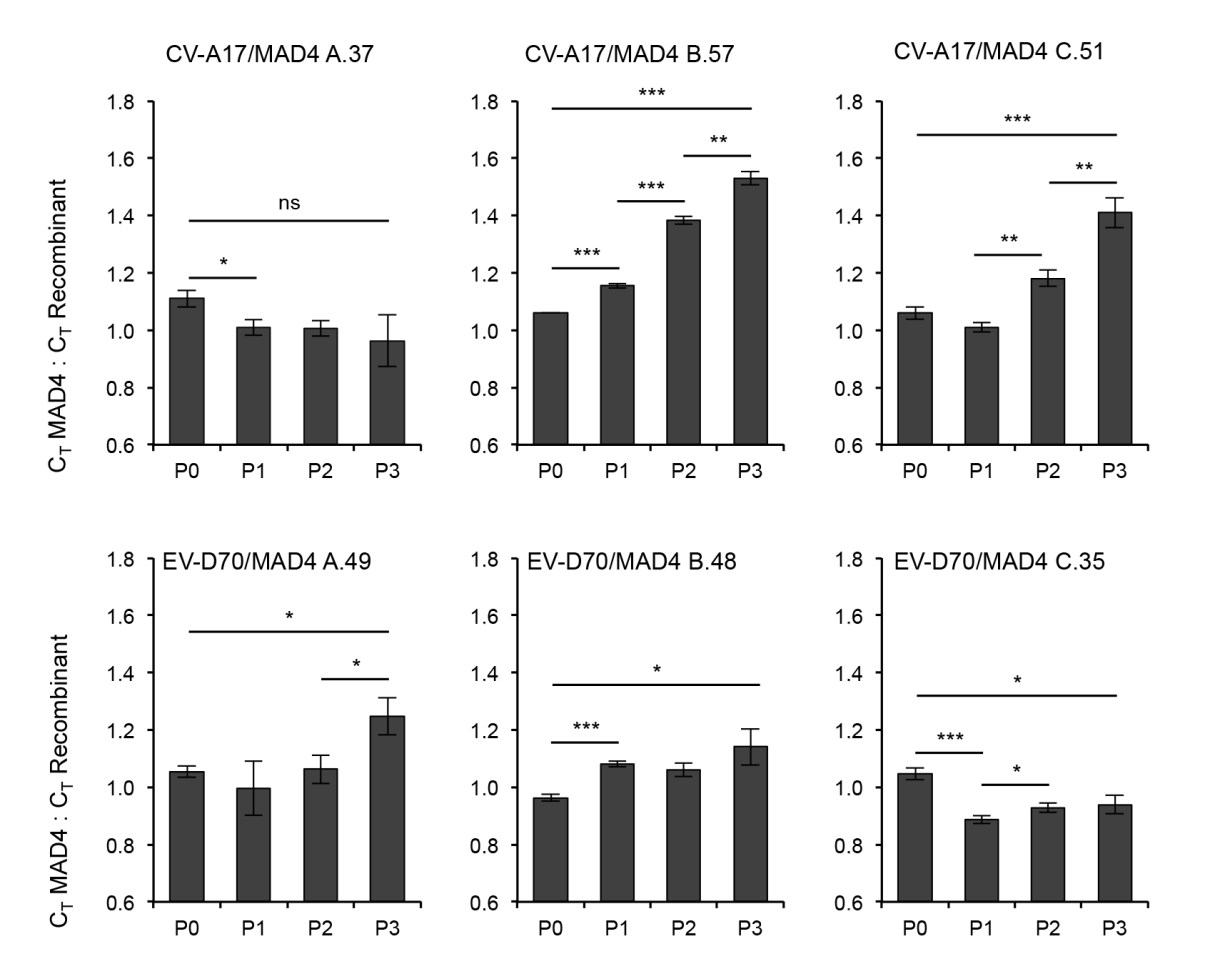
Competition assays comparing the fitness of MAD4 and selected CV-A17/MAD4 or EV-70/MAD4 homologous recombinants. HEp-2c cells were infected in triplicate with a 1:1 mix of a recombinant virus and the parental strain MAD4 at an MOI of 0.01 TCID50/cell for three passages. The proportion of each virus at each passage was determined by real time RT-PCR and expressed as the ratio of the CT values of MAD4 competitor versus recombinant. The mean ± the standard deviation is shown (Student’s t-test, n = 3; *P<0.05, **P<0.01, ***P<0.001). A CT value ratio above 1 indicates that the recombinant is fitter than MAD4.

The exchange of 5’ UTR within the same 5’ UTR group did not appear to affect negatively essential viral functions (Fig 8). Indeed, the fitness of CV-A17/MAD4 A.37 was similar and that of EV-D70/MAD4 C.35 was slightly lower than that of MAD4. The fitness of EV-D70/MAD4 A.49 and that of B.48 appeared to be slightly higher than that of MAD4 after the three passages. By contrast, the group II/I recombinant E25/MAD4 A.54 could not be detected by real-time RT-PCR after the first passage with MAD4.

Interestingly, CV-A17/MAD4 B.57 and C.51, which have a recombination site between dV and dVI and within spacer 2, respectively, were clearly fitter than MAD4 (Fig 8). We confirmed this visually in sequencing chromatograms by following changes during passaging to the height of overlapping peaks corresponding to nt that differentiate each genome. These data indicated that the acquisition of a 5’ UTR from CV-A17 can improve the fitness of MAD4 in particular cells.

The 5’ UTR is known to interact directly and indirectly with the 3’ end of the genome and the non-structural proteins, such as 2B, 3A, 3CD and 3D [69–71]. The 3’ half of the MAD4 genome is derived from CV-A sequences. It is therefore unclear whether the viability and characteristics of the group I/I recombinants, particularly CV-A17/MAD4 recombinants (B.57 and C.51), result from interaction of the 5’ UTR with the CV-A-related 3’ half of the MAD4 genome or the function of the 5’ UTR itself. We replaced the CV-A-related 3’ half of the two CV-A17/MAD4 genomes with the genuine S2 homologous sequences, as previously described for MAD4 in the MAD4.2A/S2 strain [34], to distinguish between these two hypotheses (S7A Fig). In competition experiments, both CV-A17/MAD4.2A/S2 viruses were slightly fitter than MAD4.2A/S2 (S7B Fig). However, the gain in fitness appeared to be smaller than that observed with the two CV-A17/MAD4 viruses (Figs 8 and S7). Thus, the essential interactions between the 5’UTR and the 3’ half of the genome and related proteins are not impaired in the CV-A17/MAD4.2A/S2 recombinants. In addition, these data suggest that the high level of fitness of the CV-A17/MAD4 recombinants may be due to both the function of the CV-A17 5’ UTR itself and its interaction with the CV-A-related 3’ half of the MAD4 genome.

## Discussion

In this study, we used an *in vitro* system targeting recombination to the 5’ UTR to evaluate how the PV genome tolerates 5’ UTRs from other EVs to improve our understanding of the natural evolution of cVDPVs through recombination. Surprisingly, a cVDPV genome made defective by mutagenesis of the 5’UTR could be rescued following transfection of cells with 5’ UTR sequences from the four different human EV species. In this model, we cannot determine whether recombinants were produced by the nonreplicative recombination mechanism (breakage-ligation of genomic RNAs) or the replicative one (template switching of the RNA polymerase), or both. In any case, most recombinants were nonhomologous, consistent with previous studies by our group and others using experimental models based on the rescue of defective viral genomic RNA fragments following the co-transfection of cells [5–7].

### The 5’UTR is highly permissive to genetic exchanges

This study is the first to explore in detail the permissiveness of genetic exchanges in the 5’ UTR between PV and NPEVs by analyzing randomly selected intertypic recombinants obtained in cotransfection experiments designed to mimic the likely events following the co-infection of cells in humans. Characterization of the recombinants obtained between the four species of NPEVs and PV indicated that the complete 5’ UTR including the CL and the IRES domains of the NPEVs used was functionally compatible with that of the PV genome, or at least with that of the PV/CV-A recombinant MAD4. Previous experiments using a few genetically engineered recombinants showed that the IRES of PV can be replaced with that of EVs like CV-B3 or rhinovirus, or even by that of viruses belonging to different genus (e.g. cardioviruses) without affecting substantially viral multiplication [49–51, 72, 73]. The replacement of the CL of PV with that of CV-B3, -B4 or RV-A2 was compatible with PV replication, but the CL of rhinovirus RV-B14 made the PV genome non-infectious [41, 50–52, 73].

In addition, the length of the 5’ UTR varied greatly, since recombinants could tolerate long insertions of up to 573 nt mainly in spacer 1 and the dVII-spacer 2 region. Such plasticity had been found in the 5’ UTR of *in vitro* selected PV recombinants and in a viable but unstable engineered recombinant with a double IRES from PV and encephalomyocarditis virus [5, 72]. The CL interacts with the IRES and they probably modulate the function of each other [74–77]. Nevertheless, in our experiments, additional sequences between the CL and a complete IRES did not seem to affect the three-dimensional folding and function of the 5’ UTR, confirming that the CL and IRES are functionally independent to some extent [51]. The dVII-spacer 2 region between dVI and the authentic initiation codon can tolerate modifications without significant phenotypic changes [46, 47]. We found diverse and profound modifications in this region, including insertions and deletions up to 130 nt. Of note, 10 recombinant genomes lacked part of or the entire dVI, the last IRES domain, confirming that this stem-loop is dispensable at least *ex vivo* [44, 78]. Among the 12 natural recombinant type 2 cVDPV lineages with NPEV 5’ UTR sequences which have been described so far, most were homologous recombinants; however, one showed a shorter spacer 2 of a dozen nt suggesting that a deletion occurred during recombination [21, 35, 36, 38].

### Constraints on genetic exchanges and recombination hotspots

Most recombinants with recombination sites in spacer 1 (class A recombinants) or within the dVII-spacer 2 region (class C) were essentially nonhomologous recombinants. By contrast, almost all genomes containing a recombination site located in the IRES region (dII to dVI, class B recombinants) were homologous recombinants, which reflects high constraints on the length and secondary structure of the IRES, consistent with previous reports [79–83]. Of note, the three recombinants with a recombination site in the CL were also homologous suggesting constraints on the length and structure of the CL (S8 Fig).

The location of three recombination hotspots is likely to be determined by RNA structure and/or functional constraints. Spacer 1 and the dVII-spacer 2 region (HS_A_ and HS_C_, respectively) may have been selected as recombination hotspots because of their capacity to tolerate profound modifications, as described above. However, the location of the HS_B_ between dV and dVI was surprising. The potentially independent functions of dV and dVI may favor the creation of viable recombinants. Another explanation, which is consistent with the copy-choice recombination mechanism [60] implicated in primary recombination events or subsequent rearrangements, is the presence of a large polypyrimidine tract (PPT) between the two domains. This PPT may act as a privileged sequence for polymerase template switching, by favoring fraying or dissociation from the donor template. Such U-rich sequences promote recombination in the plant viruses Brome Mosaic Virus (BMV) [84] and Tomato Bushy Stunt Virus (TBSV) [85], and possibly in PV and rhinoviruses [86–88].

Structural constraints, possibly at the protein level, may also limit recombination in the ORF. Although 5’ partners were designed to allow recombination in the VP4-VP2 capsid region, only two homologous recombination sites corresponding to the N-terminal part of VP4 were found in this part of the genome.

Among the natural recombinant type 2 cVDPV lineages with NPEV 5’ UTR sequences, eight showed recombination sites in HS_C_ and two in HS_B_. The other two resulted from recombination in domains dV or dVI. Recombination in HS_A_ has not been described so far in natural cVDPVs. All nonhomologous recombinants with insertions isolated in this study were prone to evolve into homologous one (see below and [6, 7]) in infected hosts; therefore, the features of our *in vitro* selected recombinants correlate well with those of natural cVDPVs described so far, which validates our experimental model.

A comparative analysis of a large number of complete picornavirus genomes suggested that recombination sites were not randomly distributed, instead being located in hotspots flanking genomic regions with very low rates of recombination [18, 89]. The existence of recombination hotspots in intertypic recombinants was experimentally demonstrated, in the genomic region encoding nonstructural EV proteins, in a previous study [6], and in the 5’ UTR in this study. Successive recombination events involving these hotspots in EVs would lead to exchanges of modules and to the construction of mosaic intertypic genomes through a modular evolution process. In this study, the three hotspots identified flanked three functional regions—CL, IRES dII to dV, and IRES dVI—that may be considered to constitute recombination modules. This new concept of modular evolution for EVs requires further evaluation. In particular, it is not clear whether these modules result from a mechanistic replication process favoring recombination hotspots or selection acting on and preserving viral functions (functional modules).

### Recombination differentiates groups I and II 5’ UTRs

In contrast to the group II/I recombinants, almost a third of the group I/I recombinants had a recombinant IRES, indicating that IRES dII to dVI of the 5’ partners (CV-A17, -A13, or EV-D70 isolates) were able to replace functionally those of MAD4. In addition, the exchanges of 5’ UTR sequences or domains within the same 5’ UTR group had little if any negative effect on viral replication, fitness or neurovirulence. By contrast, the group II/I recombinants rarely showed crossover junctions in the IRES sequence. In addition, all group II/I recombinants exhibited replication defect in the intestinal and neuronal cell lines tested, and/or lower neurovirulence than group I/I recombinants. Thus, the emergence of group I/I recombinants is probably favored over that of group II/I recombinants. Indeed, we found that all the 5’ UTR NPEV sequences present in natural recombinant cVDPVs [21, 35, 36, 38] segregate into molecular phylogenetic trees with sequences of NPEV types with a group I 5’ UTR.

### RNA recombination and genomic rearrangements

As hypothesized for the Hepatitis C virus and EVs including PV, the recombination of at least some plus strand RNA viruses is thought to be biphasic and to involve the generation of initial nonhomologous recombinants which evolve into homologous recombinants through subsequent genomic rearrangements, which can be considered intramolecular recombination events [6, 7, 9, 90]. Indeed, most natural cVDPVs described so far are homologous recombinants [21, 29–32, 35–38]. Consistent with this model, the four recombinants with insertions selected in this study generated homologous recombinants upon serial passaging. Interestingly, the genomic rearrangements occurred faster for the group I/I recombinants than for the group II/I recombinants (Table 2). In addition, for each recombinant class, homologous recombinants were more frequent in group I/I than in group II/I (2.3, 1.5 and 3.2 times for classes A, B and C, respectively). Our previous and present data strongly suggest that genomic rearrangements are facilitated by high sequence similarity [6]. Previous studies of PV, BVDV, BMV, tombusviruses, but also more distant retroviruses and Hepatitis delta virus, have shown that sequence homology is correlated with the overall frequency of recombination [7, 60, 91–95].

### Recombination in the 5’ UTR and cVDPV evolution

The isolate CV-A17.67591 used in this study was found co-circulating with the cVDPV MAD4 in Madagascar and CV-A17 related ancestors are thought to be the donor strains of non PV sequences present in the MAD4 genome [29]. We previously demonstrated that CV-A17.67591 is a recombination partner of MAD4 in the non-structural region and that CV-A17 non-structural sequences contribute to the phenotypic characteristics of MAD4 [12, 34]. In the current study, we show that the acquisition by MAD4 of the complete 5’ UTR from CV-A17, or the first five stem-loops corresponding to the CL and the minimum IRES [78], produces CV-A17/MAD4 recombinants that are as neurovirulent as the MAD4 parental strain in transgenic mice. In addition, these two recombination events increase viral fitness *in vitro*, supporting the hypothesis that intertypic recombination can in certain cases modify the phenotype of recombinants, favoring the emergence of recombinant lineages [12, 34, 96], such as the tripartite CV-A17/MAD4 (CV-A17/S2/CV-A) recombinant, through circulation in humans.

No bipartite recombinant cVDPVs with the 5’ UTR from an NPEV and the rest of the genome from PV have been reported to date [21, 29–32, 35–38]. Thus these bipartite recombinants may tend to evolve more rapidly into tripartite recombinants than bipartite recombinants with the 3’ half of the genome from NPEV, such as MAD4. Nevertheless, bipartite recombinants with the 3’ half of the genome from NPEV may be the major source of tripartite recombinants. One possible reason for this evolution process may be the need for recombinants to optimize the interaction between the 5’ UTR and the 3’ end of the genome and the non-structural proteins playing key roles in viral replication [69–71].

In conclusion, we show that a recombinant cVDPV genome can recombine further with the 5’ UTR sequences from the four human EV species, leading in some cases to a gain of fitness. This study illustrates how a positive strand RNA virus can acquire a mosaic recombinant genome through inter or intra-species genomic exchanges, thus favoring the emergence of new recombinant lineages.

## Materials and Methods

### Cells, viruses and viral infections

Human epithelial HEp-2c and Human rhabdomyosarcoma RD cells were grown in Dulbecco’s Modified Eagle Medium (DMEM; PAA, Pasching, Austria) with 4.5 g/L glucose, tricine and biotine supplemented with 2 mM L-Glutamine (Gibco/Thermo Fisher Scientific, Waltham, MA) and 5% (vol/vol) heat-inactivated new born calf serum (NBCS; PAA). Murine L20B cells [66] were grown in DMEM supplemented with 2 mM L-Glutamine and 10% NBCS. Human epithelial Caco2 cells were cultured in DMEM with 4.5 g/L glucose and L-Glutamine supplemented with 10% heat-inactivated fetal bovine serum (FBS; Gibco) and 1% MEM non-essential amino acid solution (Sigma-Aldrich, St. Louis, MO). Human neuroblastoma IMR5 cells were cultured in DMEM (Gibco) supplemented with 10% FBS. All cells were grown as monolayers and passaged with trypsin-EDTA (Gibco).

The cVDPV strain MAD4 was isolated from stool specimens of a patient with poliomyelitis during the 2002 outbreak in Tolagnaro district, Madagascar [29, 64]. The MAD4 virus used in this study was obtained from pBR-MAD4, a plasmid containing the full-length MAD4 cDNA downstream from the phage T7 RNA polymerase promoter [34]. A viral stock was generated in HEp-2c cells at 37°C and stored at -20°C until use.

The coxsackie A virus CV-A17.67591 and CV-A13.67900 strains and the echovirus E25.68143 strain were isolated in 2002 from stools of healthy children living in the district of Tolagnaro and grown in HEp-2c cells [29].

The coxsackie B virus CV-B4.72484 strain was isolated in 2004 from stool specimens of a healthy child in Toliara province, Madagascar, and amplified in RD cells (kind gift from Richter Razafindratsimandresy).

The EV-A71.C08-041 strain was isolated in 2008 from stool specimens of apparently healthy children in Cameroon and grown in RD cells [97].

The CV-A13 prototype strain Flores and the EV-D70 prototype strain J670-71 were kindly supplied by the National Institute of Public Health and the Environment (RIVM, Bilthoven, The Netherlands) and amplified in HEp-2c and RD cells, respectively.

In all experiments described here, cells grown in a monolayer were inoculated with viruses at the indicated MOI for 30 min. Cells were then washed twice and incubated at 37°C in a 5% CO_2_ incubator (corresponding to time zero post-infection). Total virus yields (extracellular and intracellular) from infected cells were collected after freezing and thawing to release intracellular viruses. Virus titers were evaluated in HEp-2c cells by determining the number of 50% tissue culture infective dose units (TCID_50_) per ml, as previously described [98].

### Plasmids and *in vitro* transcription

#### Construction of the recombination partner cDNAs

*3’ partner*. Nt 2 to 8 (TAAAACA) of the MAD4 cDNA present in the plasmid pBR-MAD4 were replaced with their complementary sequence (attttgt) by PCR with the QuikChange Lightning site-directed mutagenesis kit (Stratagene/Agilent Technologies, Santa Clara, CA), according to the manufacturer’s instructions, with the forward and reverse primers described in S1 Table. After ligation and the transformation of JM109 chemocompetent bacteria (Promega, Madison, WI), the cloned mutated viral genome pBR-mCL-MAD4 was obtained.

*5’ partners*. Viral RNAs of CV-A13.Flores, CV-A13.67900, EV-D70.J670-71, EV-A71.C08-041, E25.68143 and CV-B4.72484 strains were extracted from viral stocks, with the QIAamp Viral RNA Mini Kit (QIAGEN, Hilden, Germany), according to the manufacturer’s instructions. To obtain the plasmid containing the 5’ partner cDNA of each strain, a PCR fragment corresponding to the entire 5’ UTR-VP2 region and the first 18 codons of the VP3 coding region was amplified with specific 5’ phosphorylated primers containing the T7 RNA^pol^ promoter and NotI cloning restriction site (S1 Table). The fragment was amplified in a single step, using the SuperScript One-Step RT-PCR with Platinum *Taq* Polymerase System (Invitrogen/Thermo Fisher Scientific), according to the manufacturer’s instructions. The reaction mix was adjusted to a final concentration of 1.6 mM MgSO_4_. The thermocycler incubation conditions were as follows: 50°C for 30 minutes and 94°C for 2 minutes, followed by 35 cycles of 15 sec at 94°C, 30 sec at 60°C, 2 minutes at 72°C, and a final extension step of 5 minutes at 72°C. To construct the MAD4 5’ partner, pBR-MAD4 was used as the template and PCR was performed with Phire Hot Start II DNA polymerase (Finnzymes/Thermo Fisher Scientific) according to the manufacturer’s protocol. The obtained PCR products were separated by agarose gel electrophoresis and purified with the Wizard SV Gel and PCR Clean-Up System (Promega). Plasmid pBR322 (NEB, Ipswich, MA) was precut with EcoRV and desphosphorylated, and amplicons were cloned into the plasmid by transforming 5-alpha F’*I* ^*q*^ chemocompetent bacteria (NEB) and the cloned cDNA fragments and flanking sequences were sequenced.

#### Construction of recombinant CV-A17/MAD4.2A/S2 B.57 and C.51 cDNAs

The cDNAs of recombinant viruses CV-A17/MAD4.2A/S2 B.57 and CV-A17/MAD4.2A/S2 C.51 were obtained by replacing nt 1 to 565 and 1 to 684 from MAD4, respectively, by nt 1 to 568 and 1 to 689 from CV-A17.67591, in pBR-MAD4.2A/S2, a plasmid containing the cDNA of a chimeric genome consisting of the 5’ half of MAD4 (nt 1 to 3830) and the 3’ half of Sabin 2 (nt 3831 to 7455), downstream from the T7 RNA polymerase promoter [34]. The procedure used to construct the two plasmids was adapted from a previously described fusion PCR-based procedure [99]. Briefly, PCR fragments corresponding to the linearized pBR-MAD4.2A/S2 plasmid with a deletion of nt 1 to 565 and 1 to 684 (vectors) were produced with the QuikChange Lightning site-directed mutagenesis kit (Stratagene/Agilent Technologies), according to the manufacturer’s instructions, with the forward and reverse primers described in S1 Table. Overlapping PCR fragments containing nt 1 to 568 and 1 to 689 of CV-A17.67591 (inserts) were obtained by PCR (Phire Hot Start II DNA polymerase; Finnzymes/Thermo Fisher Scientific) from pBR-CV-A17, a plasmid containing the full-length cDNA under the control of a T7 RNA polymerase promoter [12]. Each vector was fused with its insert, ligated (T4 DNA Ligase; NEB) and used to transform 10-beta chemocompetent bacteria (NEB), for the generation of the two plasmids containing the cDNAs of recombinant viruses CV-A17/MAD4.2A/S2 B.57 and CV-A17/MAD4.2A/S2 C.51.

#### *In vitro* transcription

All the engineered plasmids were linearized by digestion with NotI. To obtain the last 5’ partner from CV-A17.67591, plasmid pBR-CV-A17 was linearized with NcoI (nt 2275) and SnaBI (nt 2831) at codon 170 of the VP3 region and codon 116 of the VP1 region, respectively.

The T7 RNApol promoter located upstream from the cloned viral cDNAs was used to transcribe infectious RNAs from linearized plasmids (T7 RiboMAX Large Scale RNA Production System, Promega). DNA templates were eliminated by RQ1 RNase-free DNase and viral RNA was purified with the RNeasy Mini Kit (QIAGEN) and quantified on an ND-1000 (NanoDrop/Thermo Fisher Scientific) spectrophotometer.

### Co-transfection assays in semisolid medium

Cells were transfected with a mixture of each RNA recombination partner pair and maintained in semisolid medium, to allow viral plaque formation. Six-well culture plates were seeded with 2 x 10^6^ HEp-2c or 4 x 10^6^ L20B cells per well, in their appropriate culture medium. After 24h, cell monolayers were transfected with different amounts of RNA recombination partners, from 2.5 μg to 50 ng of each RNA per well, in the presence of Lipofectamine 2000 (Invitrogen), as previously described [6]. Plates were subsequently maintained under a 0.9% agarose (Type II-A, Sigma-Aldrich) or a 1.2% Avicel (FMC Biopolymer, Philadelphia, PA) overlay at 34°C in a 5% CO_2_ incubator, until viral plaques appeared. To determine the number of plaque forming units (pfu) per μg of transfected RNA, avicel-containing medium was removed, cells were washed twice with phosphate-buffered saline (PBS) (without CaCl_2_, MgCl_2_) and stained with crystal violet.

### Isolation of recombinant viruses

Plaques formed by recombinant viruses under agarose overlay were picked and transferred to 500 μl of DMEM (PAA) supplemented with 2 mM L-Glutamine and 200 U/mL penicillin/streptomycin (P/S; Life Technologies/Thermo Fisher Scientific). Viral stocks at passage P1 were obtained following the infection of HEp-2c or L20B cells in 12-well culture plates with 250 μl of picked plaques in 750 μl of DMEM supplemented with 2 mM L-Glutamine, 200 U/mL P/S and 2% (vol/vol) FBS and incubation at 37°C in a 5% CO_2_ incubator. This P1 stock was used for the analysis of recombinant genomes. Recombinant viruses selected for further experiments were plaque-purified one or two more times on HEp-2c cells, to prevent the production of mixed viral stocks. Virus stocks were generated after two passages on HEp-2c cells and were stored at -20°C until use.

### Analysis of recombinant genomes: RT-PCR and sequencing

The oligonucleotides used for RT-PCR and sequencing of viral stocks at passage P1 are shown in S2 Table. The complete genomes of the recombinant viruses selected for further experiments were sequenced with specific primers previously described [12, 100]. Viral recombinant RNA was extracted from viral supernatants with High Pure Viral RNA kit (Roche, Basel, Switzerland), according to the manufacturer’s instructions. Reverse transcription was performed as described by Bessaud *et al*. [101], with the HeptaN primer. PCR products were obtained with Taq DNA polymerase (Taq CORE kit 10, MP Biomedicals, Santa Ana, CA), according to manufacturer’s protocol, analyzed by electrophoresis on ethidium bromide-stained agarose gels and sequenced by Eurofins Genomics on an ABI Prism 3730XL 96-capillary DNA Analyzer automated sequencer (Applied Biosystems/Thermo Fisher Scientific), with the primers used for PCR. Sequences were aligned and compared using CLC Main Workbench 6.5 software (CLC bio/QIAGEN).

### Viral plaque assays

Cell monolayers in six-well culture plates (2 x 10^6^ per well) were washed twice with DMEM without serum, and then infected with 500μl per well of 1:10 serially diluted viral stocks. Following 30 min of viral adsorption, the media was changed and cells were incubated with Avicel at 37°C for 2 to 4 days. Plaques were then stained and their diameter was measured.

### Neurovirulence in PVR-Tg mice

The neurovirulence of the viruses was tested in homozygous PVR-Tg21 mice (generous gift from A. Nomoto) [67]. Groups containing six 8-week-old mice (equal number of males and females) were inoculated intracerebrally (IC) with 10^5^ TCID_50_ in DMEM supplemented with 2 mM L-Glutamine, 200 U/mL P/S and 2% (vol/vol) FBS (30 μl per mouse). Before IC inoculation, mice were anesthetized by the IP injection of 0.25 mg xylazine (Rompun; Bayer, Leverkusen, Germany) and 2.5 mg ketamine (Imalgene; Merial, Lyon, France) in a total volume of 100 μl PBS. The animals were examined daily for 21 days post-inoculation for paralysis and/or death. Survival curves were determined according to the Kaplan-Meir method and compared using the Log Rank test with XLSTAT software version 2015.1.01 (Addinsoft, Paris, France).

All animal model studies reported here were approved by and conducted in accordance with the guidelines of the Office of Laboratory Animal Care and the Institut Pasteur Ethics Committee. This study is registered as no. 08188 (Experimental infection of mice with poliovirus) and CETEA 2013–0066, respectively. This work was performed in appliance of the French and European regulations on care and protection of the Laboratory Animals (EC Directive 2010/63, French Law 2013–118, February 6th, 2013).

### Competition assays

For competition between homologous and nonhomologous recombinants, the fragment flanking the recombination site was amplified by RT-PCR with the primers described in S2 Table, subjected to agarose gel electrophoresis and the intensity of the band corresponding either to the nonhomologous recombinant or to its homologous competitor was measured using ImageJ 1.47 software (NIH).

When homologous recombinants were competed against the MAD4 strain, the proportion of each virus was determined by real time RT-PCR using a mixture of Taqman probes labeled with two different fluorescent reporter dyes. The oligonucleotides and probes used for real time RT-PCR are shown in S3 Table. Each 20 μl reaction contained 4 μl RNA, 10 μl of 2X Reaction Mix, 250 nM of forward primer, 500 nM of reverse primer, 250 nM of each probe, and 0.4 μl SuperScript III RT/ Platinium *Taq* Mix (Invitrogen).

For *in vivo* competition assays, a dose of 10^7.4^ TCID_50_ of a 1:1 mixture of each recombinant with the MAD4 parental strain was used for the IP inoculation (500 μl per mouse) of groups of 10 6-week-old PVR-Tg21 mice (equal number of males and females). The spinal cord of paralyzed or dead mice was removed to extract viral RNA for analysis by real time RT-PCR as described above.

## Supporting Information

S1 Fig Location of the seven secondary structure domains of the 5’ UTR. Schematic representation of the EV 5’ UTR structure described in Fig 1. The first and last nt of each stem-loop domain are given for each of the eight strains used in this study. The position of the first nt of the open reading frame is also indicated.
(TIF)

S2 Fig Examples of numbering for homologous and nonhomologous recombination sites. Nt sequences around the recombination site and the corresponding electropherogram are shown for the recombinant genome. Alignment with the corresponding sequences of the parental 5’ and 3’ partner genomes is shown. (A) In most cases, the homologous recombination junction cannot be precisely determined because it is located in the homologous genomic segment (underlined with dashed lines) between the two nt (framed in orange) differentiating the 5’ and 3’ partner sequences. In this case, the recombination site was arbitrarily located to include most of this segment from the 5’ partner and is indicated by a vertical line. Therefore, the last 5’ partner nt position and the first MAD4-specific nt position give the coordinates of the recombination site (indicated by arrows). (B) In most cases, nonhomologous recombination sites can be precisely determined following the alignment of the recombinant and parental sequences. In the example shown, the breakpoint is located at a stretch of two identical nt in the parental sequences. In this case the arbitrary coordinates of the recombination site are reported as described for panel A.
(TIF)

S3 Fig Influence of cell line on the location of recombination sites in group I/I recombinants. For legend see Fig 3.
(TIF)

S4 Fig Influence of cell line on the location of recombination sites in group II/I recombinants. For legend see Fig 3.
(TIF)

S5 Fig Predicted secondary structure of the recombinant stem-loop domains of the two homologous recombinants CV-A13.67900/MAD4 B.69 and B.55. Predicted secondary structure of the recombinant stem-loop domains of the two homologous recombinants CV-A13.67900/MAD4 B.69 (A) and B.55 (B) showing mutations at the recombination junction, in IRES domains II and VI, respectively. The predicted secondary structures of IRES domain II and VI in parental MAD4 and CV-A13.67900 strains are shown on the left of each panel (A and B). Sequences specific of CV-A13.67900 and MAD4 are shown in red and black, respectively. Only nt of the CV-A13.67900 sequence that differ from those of MAD4 are indicated for parental structures. Secondary structure predictions were generated with mfold, version 3.6 [63]. The minimum free energy in kcal/mol corresponding to the most probable structure is indicated for each prediction. Predicted secondary structure of domain II in recombinant CV-A13.67900/MAD4 B.69 (A) and domain VI of recombinant CV-A13.67900/MAD4 B.55 (B) shown at passage 2 and 7. The primary mutations present at the recombination site are indicated in lowercase. The C to T substitution that appears at passage 7 in B.69 recombinant is boxed in orange. No change was observed at passage 7 for the B.55 recombinant.
(TIF)

S6 Fig Example of the analysis of a competition assay on agarose gel. RT-PCR analysis of the competition assay comparing the relative fitness of the nonhomologous CV-B4/MAD4 A.37 and the homologous CV-B4/MAD4 B.38 recombinants. Viruses were mixed at a 1:1 ratio and HEp-2c cells were inoculated in triplicate (P0, P0’, P0”) and passaged three times (P1 to P3). Viral RNA was extracted, reverse transcribed, and the fragment flanking the recombination site was amplified by PCR. The resulting products were analyzed on agarose gel electrophoresis after staining with ethidium bromide.
(TIF)

S7 Fig Competition assays comparing the fitness of MAD4.2A/S2 and CV-A17/MAD4.2A/S2 recombinants. In MAD4 and in CVA17/MAD4 recombinants B.57 and C.51 the CV-A related sequences present in the 3’ half of the MAD4 genome were replaced by those of Sabin 2 (A). Competition experiments were performed between MAD4.2A/S2 and CV-A17/MAD4.2A/S2 B.57 or C.51 (B). For details about the method and data see legend of Fig 8.
(TIF)

S8 Fig Predicted secondary structure of the cloverleaf of the parental strain MAD4 and of the homologous recombinants CV-A13.Flores /MAD4 A.33 and A.34, and CV-A13.67900/MAD4 A.5. Sequences specific of MAD4, CV-A13/Flores and CV-A13.67900 are shown in black, orange and red, respectively. Secondary structure predictions were generated with mfold, version 3.6 [63]. The minimum free energy in kcal/mol corresponding to the most probable structure is indicated for each prediction. The C to U substitution present at the recombination site of recombinant CV-A13.67900/MAD4 A.5 is indicated by an arrow.
(TIF)

S1 Table Sequences of the primers used for engineering recombination RNA partners. (PDF)

S2 Table Oligonucleotides used for amplifying and sequencing rescued recombinants. (PDF)

S3 Table Oligonucleotides and probes used for real time RT-PCR analysis of competition experiments. (PDF)

S1 File Nucleotide sequences of the 5’ RNA partners. (PDF)

S2 File Nucleotidic features of group I/I recombinant genomes. (DOCX)

S3 File Nucleotidic features of group II/I recombinant genomes. (DOCX)

S4 File Comparative analysis of viral growth of recombinants on HEp-2c and L20B cells. (XLSX)

S5 File Nucleotide substitutions and codon changes in the whole genome of the selected recombinants, compared to parental strains. (XLSX)

S6 File P values from pairwise comparisons of survival curves after intracerebral inoculation in PVR-Tg21 mice with MAD4 and selected recombinant viruses. (XLSX)
