# Supplementary figures and images for: Evolution and Emergence of Enteroviruses through Intra- and Inter-species Recombination: Plasticity and Phenotypic Impact of Modular Genetic Exchanges in the 5’ Untranslated Region
Source: PLoS Pathog. 2015 Nov 12;11(11):e1005266. doi: 10.1371/journal.ppat.1005266 (PMC4643034; doi:10.1371/journal.ppat.1005266)

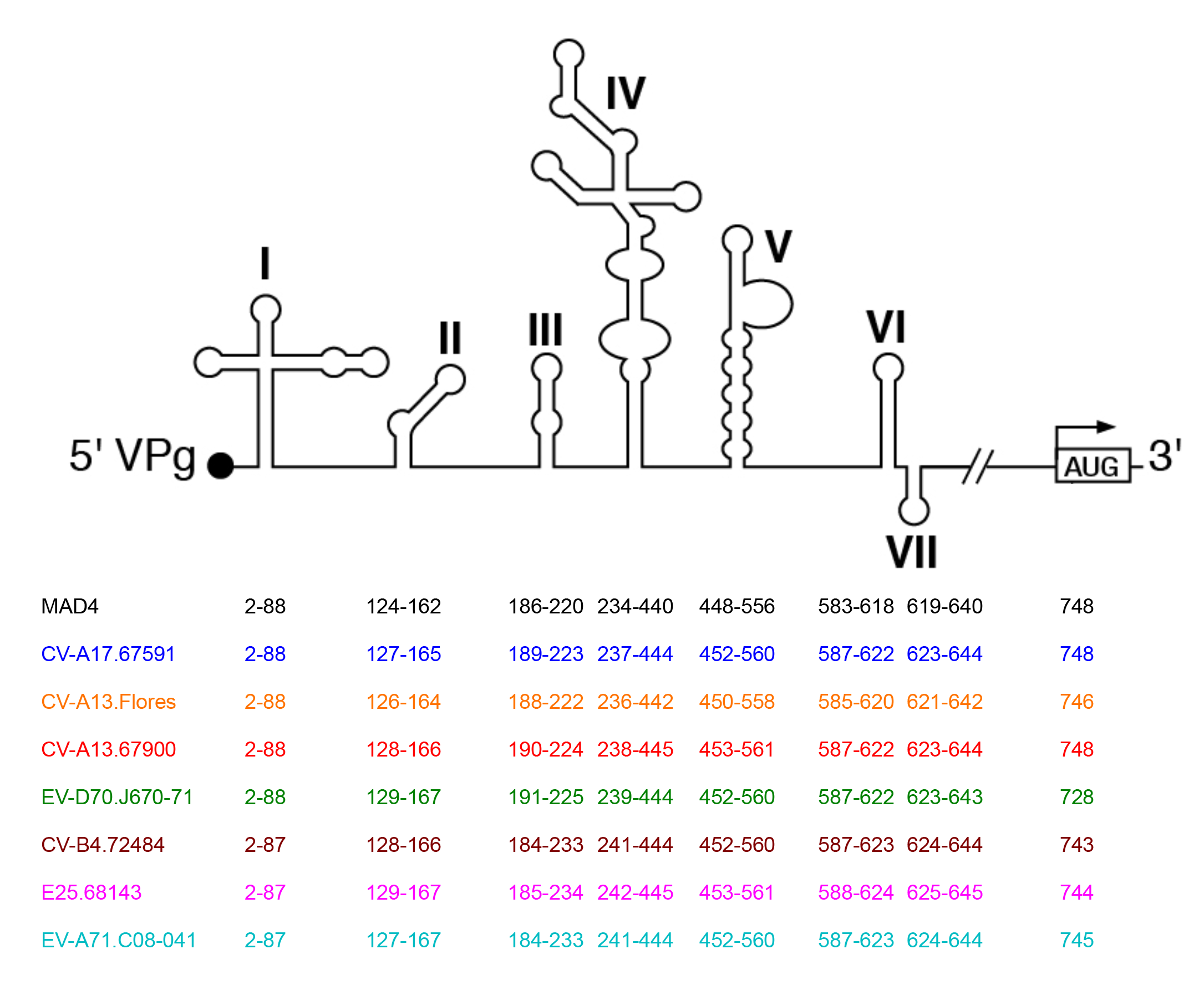

Supplement: S1 Fig — Schematic representation of the EV 5’ UTR structure described in Fig 1. The first and last nt of each stem-loop domain are given for each of the eight strains used in this study. The position of the first nt of the open reading frame is also indicated. (TIF) [file ppat.1005266.s001.tif]

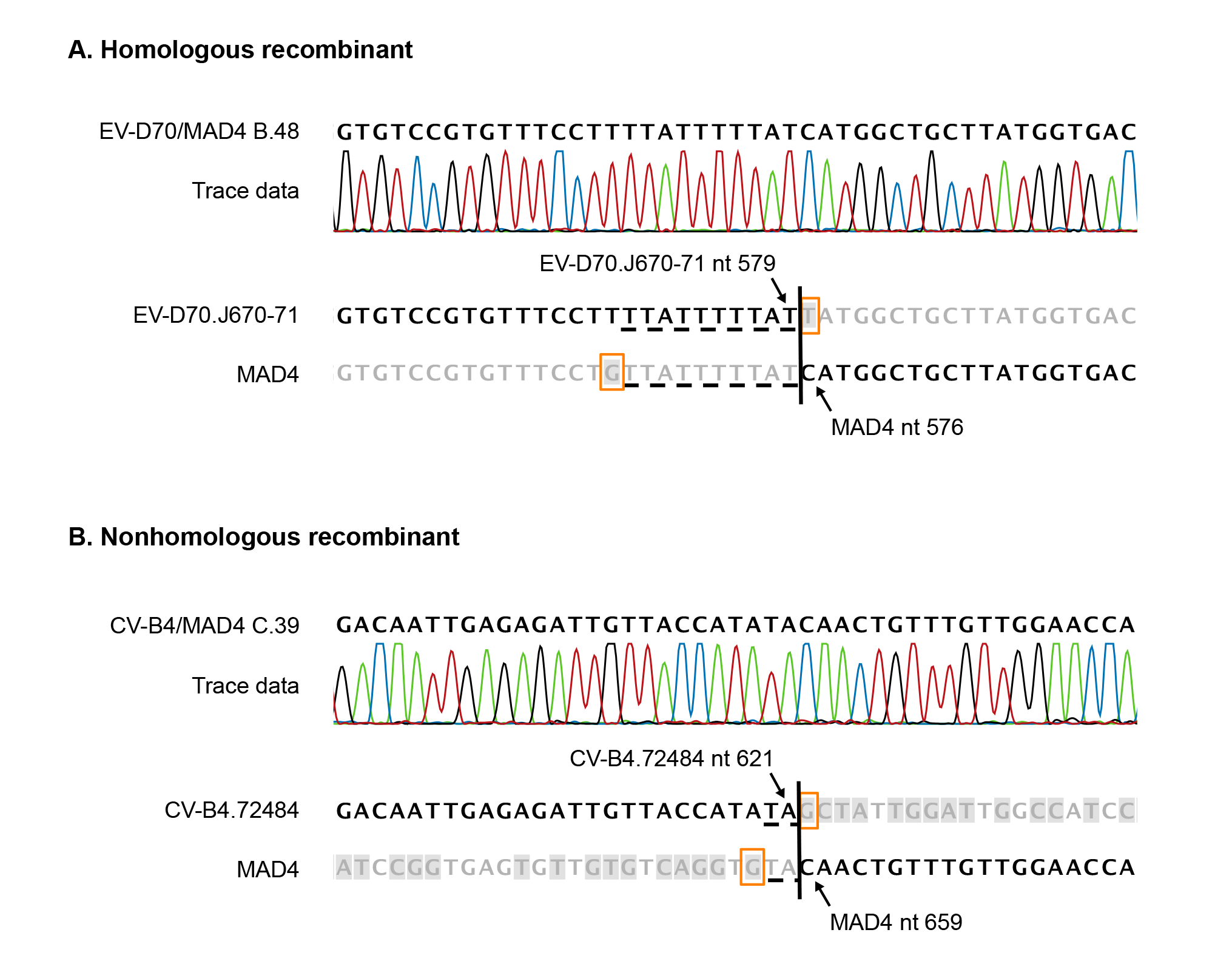

Supplement: S2 Fig — Nt sequences around the recombination site and the corresponding electropherogram are shown for the recombinant genome. Alignment with the corresponding sequences of the parental 5’ and 3’ partner genomes is shown. (A) In most cases, the homologous recombination junction cannot be precisely determined because it is located in the homologous genomic segment (underlined with dashed lines) between the two nt (framed in orange) differentiating the 5’ and 3’ partner sequences. In this case, the recombination site was arbitrarily located to include most of this segment from the 5’ partner and is indicated by a vertical line. Therefore, the last 5’ partner nt position and the first MAD4-specific nt position give the coordinates of the recombination site (indicated by arrows). (B) In most cases, nonhomologous recombination sites can be precisely determined following the alignment of the recombinant and parental sequences. In the example shown, the breakpoint is located at a stretch of two identical nt in the parental sequences. In this case the arbitrary coordinates of the recombination site are reported as described for panel A. (TIF) [file ppat.1005266.s002.tif]

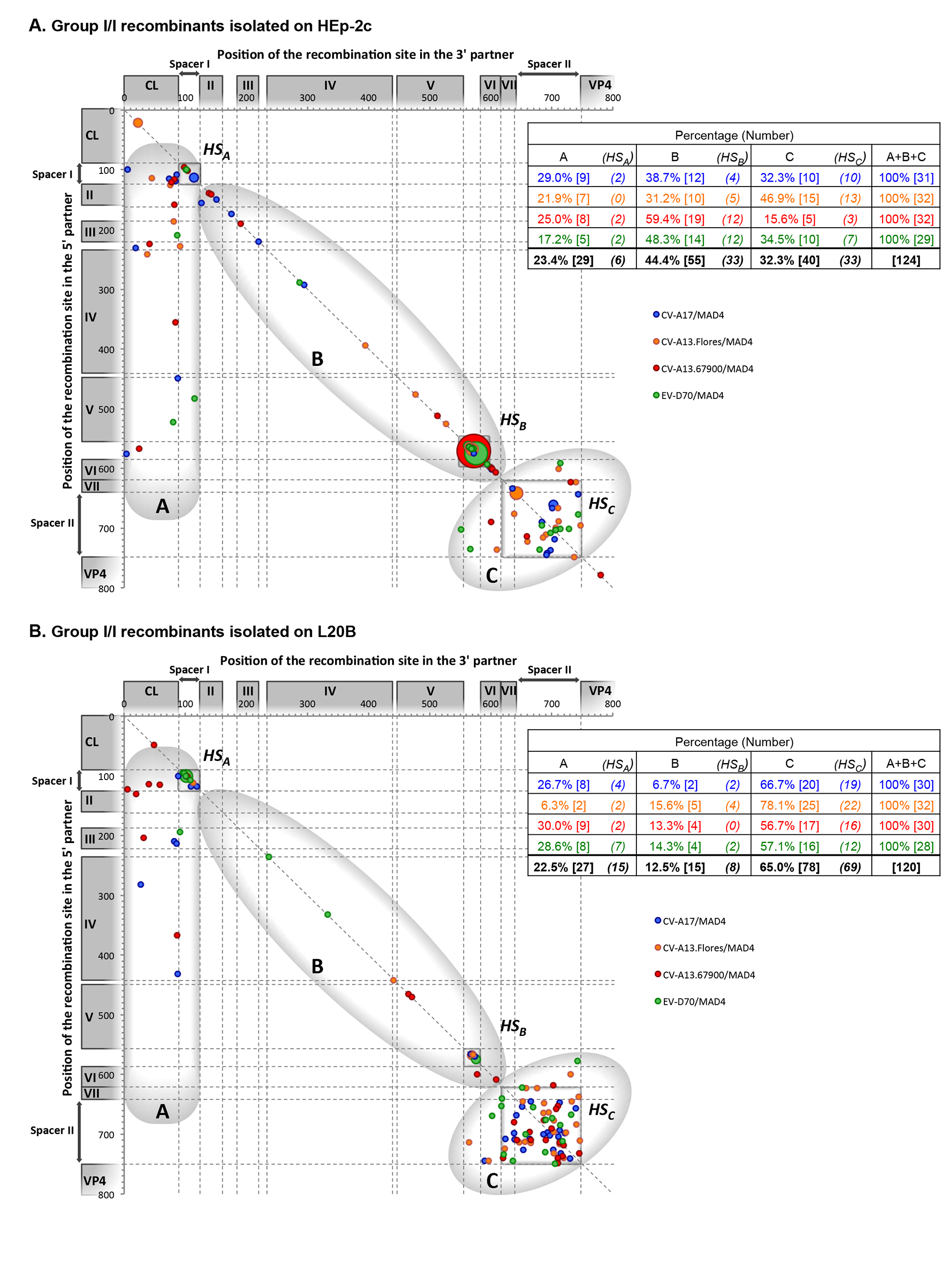

Supplement: S3 Fig — For legend see Fig 3. (TIF) [file ppat.1005266.s003.tif]

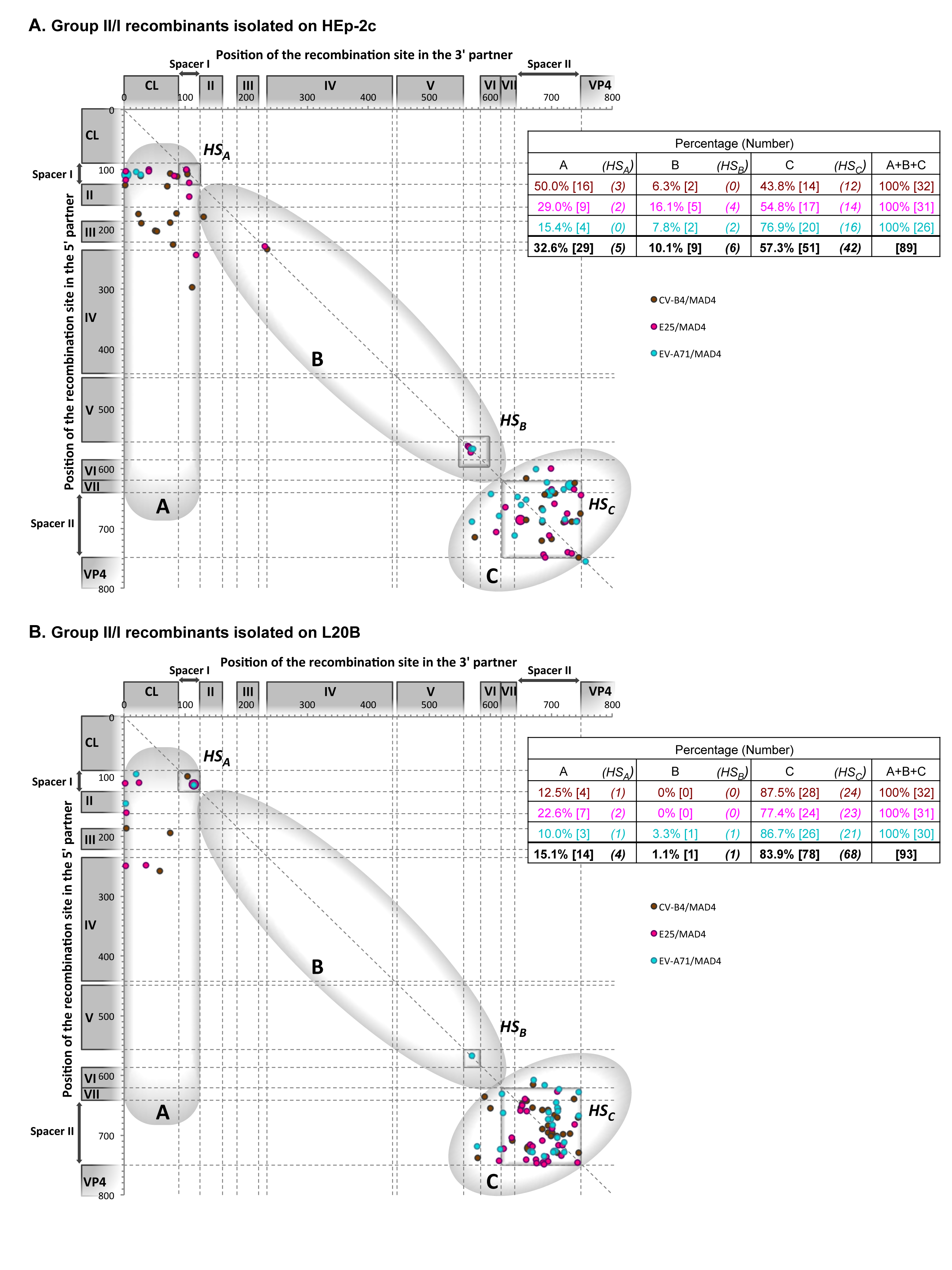

Supplement: S4 Fig — For legend see Fig 3. (TIF) [file ppat.1005266.s004.tif]

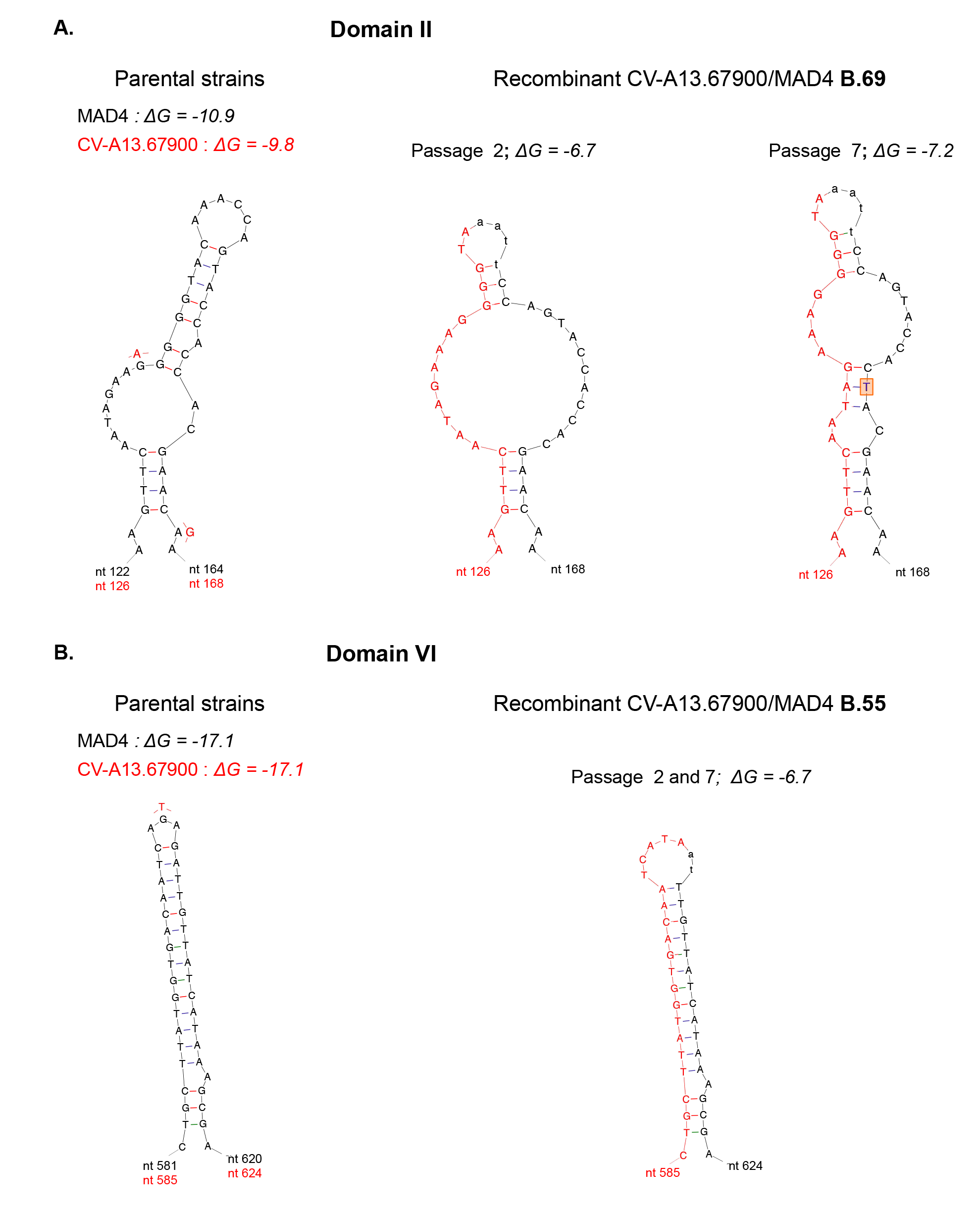

Supplement: S5 Fig — Predicted secondary structure of the recombinant stem-loop domains of the two homologous recombinants CV-A13.67900/MAD4 B.69 (A) and B.55 (B) showing mutations at the recombination junction, in IRES domains II and VI, respectively. The predicted secondary structures of IRES domain II and VI in parental MAD4 and CV-A13.67900 strains are shown on the left of each panel (A and B). Sequences specific of CV-A13.67900 and MAD4 are shown in red and black, respectively. Only nt of the CV-A13.67900 sequence that differ from those of MAD4 are indicated for parental structures. Secondary structure predictions were generated with mfold, version 3.6 [63]. The minimum free energy in kcal/mol corresponding to the most probable structure is indicated for each prediction. Predicted secondary structure of domain II in recombinant CV-A13.67900/MAD4 B.69 (A) and domain VI of recombinant CV-A13.67900/MAD4 B.55 (B) shown at passage 2 and 7. The primary mutations present at the recombination site are indicated in lowercase. The C to T substitution that appears at passage 7 in B.69 recombinant is boxed in orange. No change was observed at passage 7 for the B.55 recombinant. (TIF) [file ppat.1005266.s005.tif]

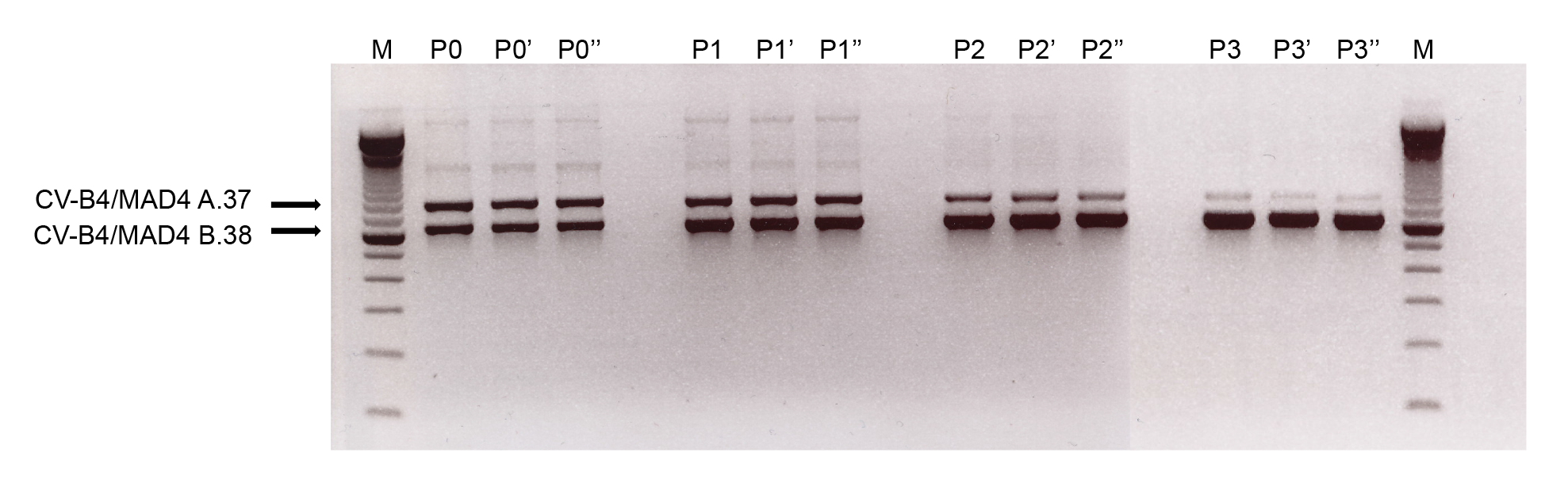

Supplement: S6 Fig — RT-PCR analysis of the competition assay comparing the relative fitness of the nonhomologous CV-B4/MAD4 A.37 and the homologous CV-B4/MAD4 B.38 recombinants. Viruses were mixed at a 1:1 ratio and HEp-2c cells were inoculated in triplicate (P0, P0’, P0”) and passaged three times (P1 to P3). Viral RNA was extracted, reverse transcribed, and the fragment flanking the recombination site was amplified by PCR. The resulting products were analyzed on agarose gel electrophoresis after staining with ethidium bromide. (TIF) [file ppat.1005266.s006.tif]

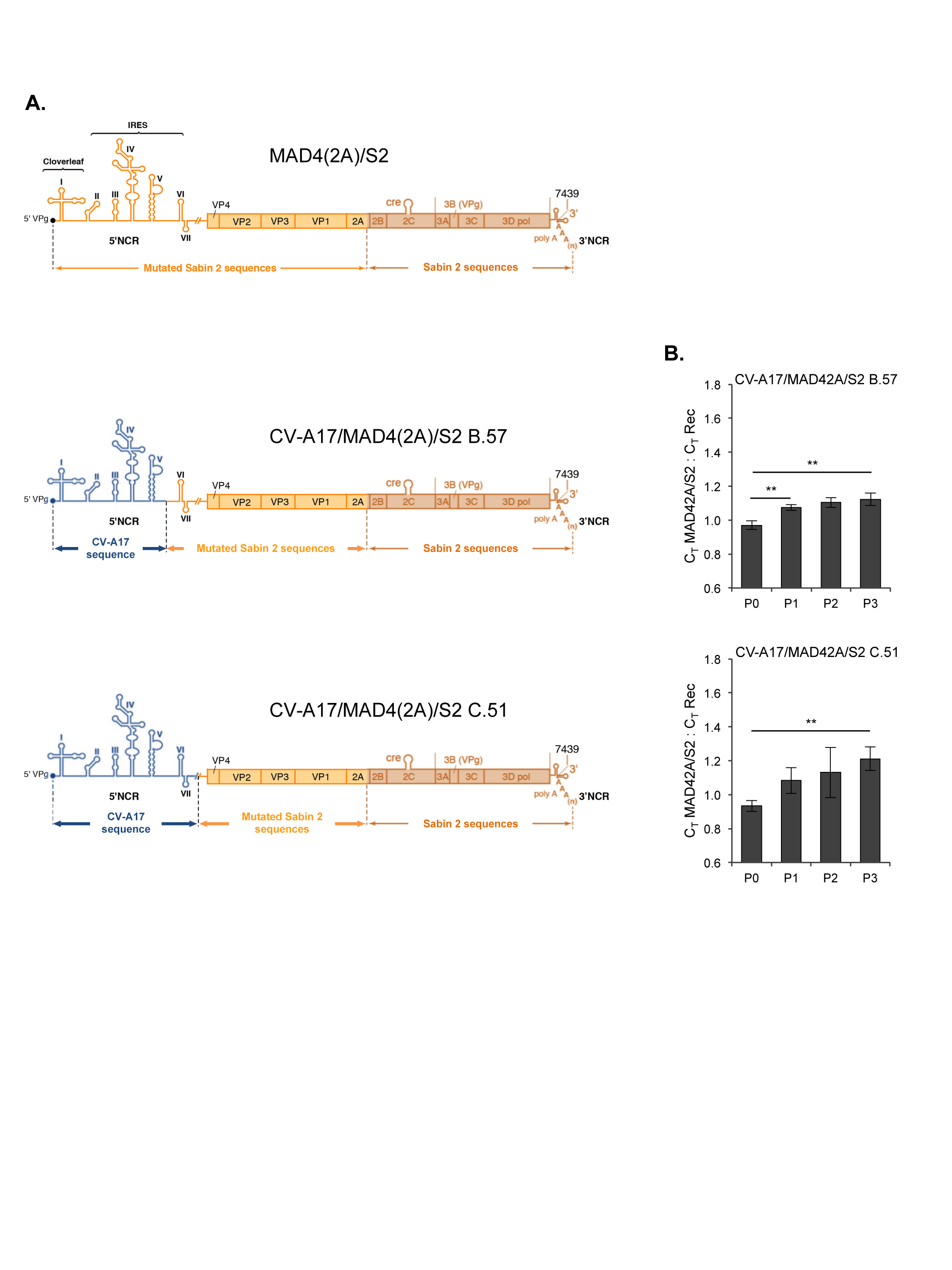

Supplement: S7 Fig — In MAD4 and in CVA17/MAD4 recombinants B.57 and C.51 the CV-A related sequences present in the 3’ half of the MAD4 genome were replaced by those of Sabin 2 (A). Competition experiments were performed between MAD4.2A/S2 and CV-A17/MAD4.2A/S2 B.57 or C.51 (B). For details about the method and data see legend of Fig 8. (TIF) [file ppat.1005266.s007.tif]

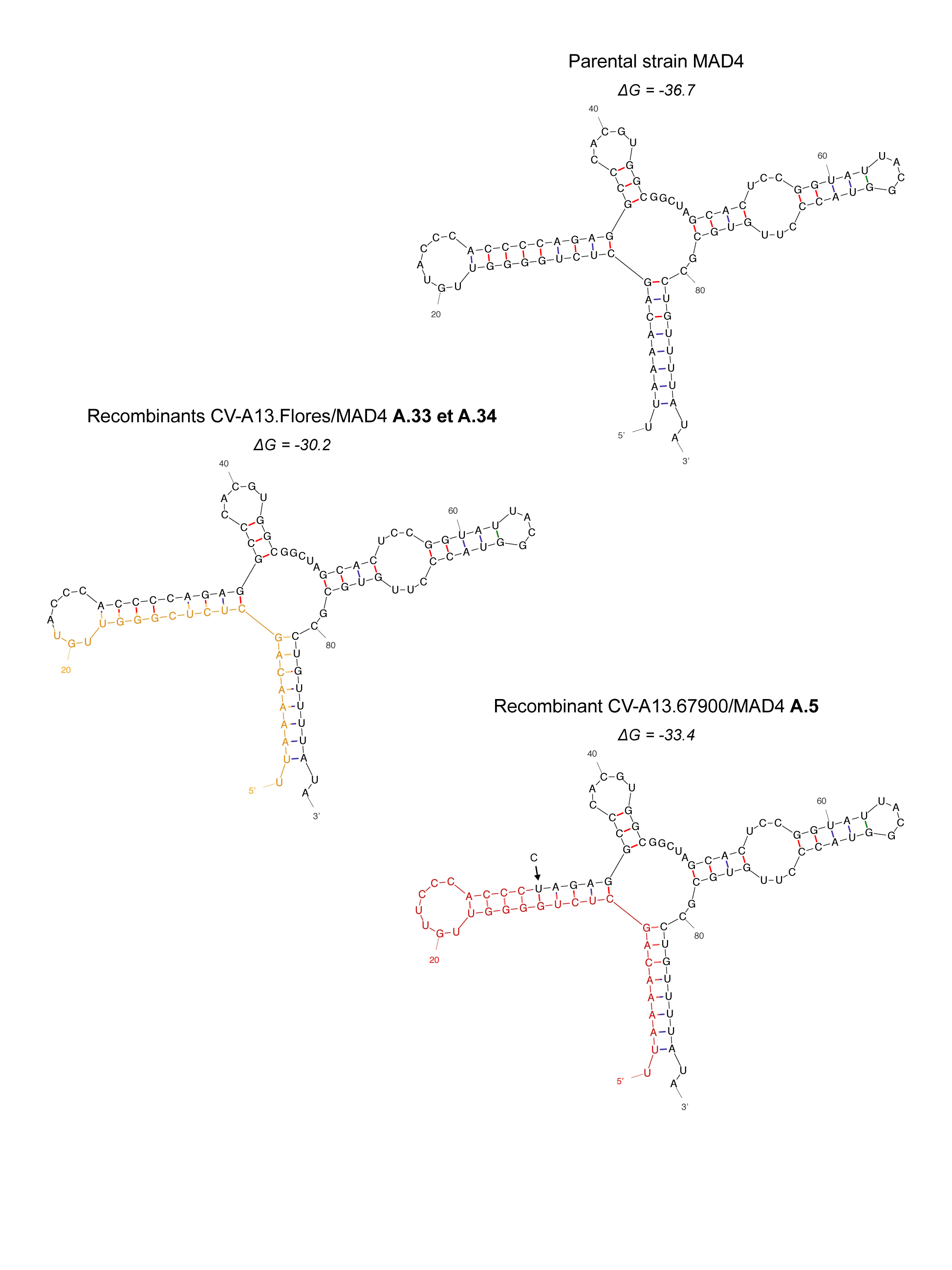

Supplement: S8 Fig — Sequences specific of MAD4, CV-A13/Flores and CV-A13.67900 are shown in black, orange and red, respectively. Secondary structure predictions were generated with mfold, version 3.6 [63]. The minimum free energy in kcal/mol corresponding to the most probable structure is indicated for each prediction. The C to U substitution present at the recombination site of recombinant CV-A13.67900/MAD4 A.5 is indicated by an arrow. (TIF) [file ppat.1005266.s008.tif]
