# Supplementary material for: Evolution and Emergence of Enteroviruses through Intra- and Inter-species Recombination: Plasticity and Phenotypic Impact of Modular Genetic Exchanges in the 5’ Untranslated Region
Source: PLoS Pathog. 2015 Nov 12;11(11):e1005266. doi: 10.1371/journal.ppat.1005266 (PMC4643034; doi:10.1371/journal.ppat.1005266)
Supplement: S1 Table — (PDF) [file ppat.1005266.s009.pdf]

S1 Table. Sequences of the primers used for engineering recombination RNA partners and recombinant CV-A17/MAD4.2/S2 B.57 and C.51 genomes.

Constructs	Sense[a]	Sequence (5'-3')[b]	Genome position[c]	Cloning site or promoter
3' partner				
mCL MAD4	F	tgggggcgcgcctaatacgactcactataggT*ATTTTGT*GCTCTGGGT	1-18*	T7 promoter
	R	5'Phos-agcttggggcaatggcaacaa		
5' partners				
For the three partners below	F	5'Phos-actaatacgactcactataggTTAAAACAGCTCTGGGGT	1-18	T7 promoter
1-1821 MAD4	R	5'Phos-agcggccgcATTGTCTGCGGTCAGGTACTG	1821-1801*	Not I
1-1821 CV-A13.67900	R	5'Phos-agcggccgcGTTATCTGAGGTCAAATACTG	1821-1801	Not I
1-1738 EV-D70.J670.71	R	5'Phos-agcggccgcGTCATCAGTAGTCAAAAATTG	1738-1718	Not I
1-1819 CV-A13.Flores	F	5'Phos-actaatacgactcactataggTTAAAACAGCTCTCGGGT	1-18	T7 promoter
	R	5'Phos-agcggccgcGTTGTCAGAAGTCAGGTACTG	1819-1799	Not I
1-1767 EV-A71.C08-041	F	5'Phos-actaatacgactcactataggTTGAAACAGCCTGTGGGT	1-18	T7 promoter
	R	5'Phos-agcggccgcATCATCAGTGGTCAAAAATTG	1767-1747	Not I
For the two partners below	F	5'Phos-cctaatacgactcactataggTTAAAACAGCCTGTGGGT	1-18	T7 promoter
1-1787 E25.68143	R	5'Phos-agcggccgcGTCATCAGACGTAAGGAACTG	1787-1767	Not I
1-1786 CV-B4.72484	R	5'Phos-agcggccgcGTCATCTGATGTCAAAAATTG	1786-1766	Not I
Vector pBR-MAD4.2A/S2	R	cctatagtgagtcgtattaggcg		T7 promoter
For CV-A17/MAD4.2/S2 B.57	F	GTTATTTTTATCATGGCTGCTTATGG	565-590*	
For CV-A17/MAD4.2/S2 C.51	F	TAGCTTCACTTCTCATTCAATCA	684-706*	
Insert pBR-CV-A17	F	5'Phos-**TTAAAACAGCTCTGGGGTTGTTCC**	1-24♮	
For CV-A17/MAD4.2/S2 B.57	R	CAATTCGCTTTATGATAACAATCTCTGATTGTCACCATAAGCAG CCATGATAAAAATAAC**AGGAAACACGGACACCCAAAGTAG**	545-568♮	
For CV-A17/MAD4.2/S2 C.51	R	TGTTGTTTTATCCTCGTATTGTTTTTGATTAATTGATTGAATGAG AAGTGAAGCTA**GAGTGGAACAAACCCAACAAACAG**	666-689♮	

[a] F, forward; R, reverse

[b] The complementary sequences of viral genomes are indicated in upper case. The nucleotide sequences of restriction sites or promoters mentioned in the last column are underlined. 5' phosphorylation is indicated. Mutations introduced in the 3' partner are indicated in italics. For the primers used to construct CV-A17/MAD4.2/S2 B.57 and C.51 (lower part of the table), complementary sequences of MAD4 are indicated in upper case, and complementary sequences of CV-A17 in bold upper case.

[c] Numbering according to the nucleotide sequence of the strain indicated in the first column. *, according to MAD4 numbering; ♮, according to CV-A17.67591 numbering
