# Supplementary material for: Evolution and Emergence of Enteroviruses through Intra- and Inter-species Recombination: Plasticity and Phenotypic Impact of Modular Genetic Exchanges in the 5’ Untranslated Region
Source: PLoS Pathog. 2015 Nov 12;11(11):e1005266. doi: 10.1371/journal.ppat.1005266 (PMC4643034; doi:10.1371/journal.ppat.1005266)
Supplement: S2 Table — (PDF) [file ppat.1005266.s010.pdf]

S2 Table. Oligonucleotides used for amplifying and sequencing rescued recombinants.

Recombinants viruses	Sense[a]	Name	Sequence (5'-3')[b]	Genome position[c]
5’ UTR group I/I recombinants	R	HEVC-996-R	TCACTRTAWCCACAVGCCTC	986-967*
CV-A13.Flores/MAD4	F	AscIT7 CVA13 Flores 1F	ggcgcgcctaatacgactcactataggTTAAAACAGCTCTCG	1-15*
other group I/I recombinants	F	AscIT7 EV70 J670 1F	ggcgcgcctaatacgactcactataggTTAAAACAGCTCTG	1-14*
5’ UTR group II/I recombinants	R	EV-985-R	TCRCTRTANCCACANKCCTC	983-964♮
EV-A71/MAD4	F	EV71 CAE041 G3A F	acgactcactataggTTAAAACAGCCTGTGGGTTG	1-20♮
other group II/I recombinants	F	AscIT7 E25 1F	ggcgcgcctaatacgactcactataggTTAAAACAGCCTGTGGG	1-17♮
Sequencing and competition assays analysis				
All recombinants	F	UG53	TGGCTGCTTATGGTGACAAT	578-597*
	R	UC53	TTGTCACCATAAGCAGCCA	569-578*
	F	MAD4-73-F	TTGTGCGCCTGTTTTAT	73-89*

[a] F, forward; R, reverse

[b] Sequences complementary to those of viral genome are indicated in uppercase.

[c] *, According to MAD4 numbering; ♮, according to EV-A71.C08-041 numbering
