# Supplementary material for: Evolution and Emergence of Enteroviruses through Intra- and Inter-species Recombination: Plasticity and Phenotypic Impact of Modular Genetic Exchanges in the 5’ Untranslated Region
Source: PLoS Pathog. 2015 Nov 12;11(11):e1005266. doi: 10.1371/journal.ppat.1005266 (PMC4643034; doi:10.1371/journal.ppat.1005266)
Supplement: S3 Table — (PDF) [file ppat.1005266.s011.pdf]

S3 Table. Oligonucleotides and probes used for real time RT-PCR analysis of competition experiments.

Mixture of viruses	Probes and primers[a]	Name[b]	Sequence (5'-3')	Genome position[c]
CV-A17/MAD4 A.37 + MAD4	Probe Rec	6FAM-CA17-39-F	ACGTGGCGGCCAGTACACTG	39-58
	Probe MAD4	HEX-MAD4-48-F	CTAGCACTCCGGTATTACGGTACCC	48-72
	Primer F	MAD4-4-F	AAACAGCTCTGGGGTTGT	4-21
	Primer R	MAD4-173-R	CAGAAGTGCTTGTTCGTG	173-156
CV-A17/MAD4 B.57 + MAD4	Probe Rec	6FAM-CA17-484-F	AGCAGGTAGTTGCAAGCCAGCA	484-505
	Probe MAD4	HEX-MAD4-482-F	CAGGCGGTCGCGAACC	482-497
	Primer F	MAD4-453-F	CCTGAATGCGGCTAATC	453-469
	Primer R	MAD4-592-R	CACCATAAGCAGCCATGAT	592-574
CV-A17/MAD4 C.51 + MAD4	Probe Rec	6FAM-CA17-635-F	CCATCCAGTGAAAGTCAGACTGATT	635-659
	Probe MAD4	HEX-MAD4-636-F	CGGTGAGTGTTGTGTCAGGTGTA	636-658
	Primer F	MAD4-581-F	CTGCTTATGGTGACAATCA	581-599
	Primer R	MAD4-707-R	TTGATTGAATGAGAAGTGAAG	707-687
EV-D70/MAD4 A.49 + MAD4	Probe Rec	6FAM-EV70-61-F	ACCCCGGTACCCTTGTACGC	61-80
	Probe MAD4	HEX-MAD4-48-F		
	Primer F	MAD4-4-F		
	Primer R	MAD4-173-R		
EV-D70/MAD4 B.48 + MAD4	Probe Rec	6FAM-EV70-494-F	CACAATCCAGTGAGTGGTTTGTCG	494-517
	Probe MAD4	HEX-MAD4-482-F		
	Primer F	MAD4-453-F		
	Primer R	MAD4-592-R		
EV-D70/MAD4 C.35 + MAD4	Probe Rec	6FAM-EV70-638-F	CCGGTGATATCTTGAAATTTTGCC	638-661
	Probe MAD4	HEX-MAD4-636-F		
	Primer F	MAD4-580-F	GCTGCTTATGGTGACAATC	580-598
	Primer R	MAD4-739-R	TGTTGTTTTATCCTCGTATTG	739-719
E25/MAD4 A.54 + MAD4	Probe Rec	6FAM-E25Mr54-88-F	TCCACCTTTCCCCTAACTTAGAAGC	88-112
	Probe MAD4	HEX-MAD4-95-F		
	Primer F	MAD4-73-F	TTGTGCGCCTGTTTTAT	73-89
	Primer R	MAD4-173-R		

[a] Specific probes of the recombinant (Probe Rec) and of MAD4 (Probe MAD4) are indicated. Generic forward (F) or reverse (R) primers used for amplifying both viruses in mixture are given.

[b] The 5' dye is indicated for each probe

[c] Most numbering is given according to MAD4 sequence, except for the probes that are specific of recombinants. In this case, numbering is that of the given recombinant (Probe Rec).
