# Supplementary material for: Evolution and Emergence of Enteroviruses through Intra- and Inter-species Recombination: Plasticity and Phenotypic Impact of Modular Genetic Exchanges in the 5’ Untranslated Region
Source: PLoS Pathog. 2015 Nov 12;11(11):e1005266. doi: 10.1371/journal.ppat.1005266 (PMC4643034; doi:10.1371/journal.ppat.1005266)
Supplement: S1 File — (PDF) [file ppat.1005266.s012.pdf]

S1 File: Nucleotide sequences of the 5’ RNA partners

```
                                       20                    40                    60                    80
                                        |                     |                     |                     |
MAD4            TTAAAACAGC TCTGGGGTTG TACCCACCCC AGAGGCCCAC GTGGCGGCTA GCACTCCGGT ATTACGGTAC CCTTGTGCGC 80
CV-A13.Flores   .......... ...C...... .T........ .......... ........C. .T........ .......... ......A... 80
CV-A13.67900    .......... .......... .T........ .......... ........C. .T..A..... ..C....... ......A... 80
CV-A17.67591    .......... .......... .T........ .......... ........C. .T..A.T... ..C....... ......A... 80
EV-D70.J670-71  .......... .......... .T........ .......... .......... .T........ .CCC...... ......A... 80
EV-A71.C08-041  .......... CTGT...... C........T CAG....... TG....-.C. ......T... .C.GA..... .T........ 79
E25.68143       .......... CTGT...... .T.......A CAG......T TG....-T.. ....A.T... ..CC...... .T........ 79
CV-B4.72484     .......... CTGT...... .T.......A CAG....... TG....-.C. ....A.T... ..C....... .T........ 79

                                      100                   120                   140                   160
                                        |                     |                     |                     |
MAD4            CTGTTTTATA CTCCCCTCCC -----GTAAC TT-AGAAGC- ACGAAACCAA GTTCAATAGA AGGGGGTACA AACCAGTACC 153
CV-A13.Flores   .......... TC...T.... CC---.C... ..-.....TT .TA..-.... .......... .......... .G........ 155
CV-A13.67900    .......... .C...T.T.. CCT--..... ..-......A .AC....... .......... .A........ .......... 157
CV-A17.67591    .......... .....T.... CC---..... ..-......C .TA..T.... ......C... ......C... .......G.. 156
EV-D70.J670-71  .......... .....T.T.. CAA--..... ..T.....AA .TA..CTA.T ......C..G .......... .......... 158
EV-A71.C08-041  .........T TC..TTC... TAAA-..... ..-......T TT...C.A.T .A...GC..T ...T.TG... T......CA. 157
E25.68143       .........C .A..TT.... CAAAT..... ..-......C CATC.TA..C .G...G...G T..CTCAGT. TG...ACTGA 158
CV-B4.72484     .........C .A...T.... CAGA-.A... ..-......A TAATCTGA.C .G........ TAACCCAGT. C....ACTGG 157

                                      180                   200                   220                   240
                                        |                     |                     |                     |
MAD4            ACCACGAACA AGCACTTCTG TTTCCCCGG- -TGACATTGC ATAGACTGCT CACGCGGTTG AAAGTGATCG -ATCCGTTAC 230
CV-A13.Flores   .......... .......... .........- -...TG...T .......... A......... ...ACAGCT. -........T 232
CV-A13.67900    .........G .......... .........- -...GG...T ...A....TC .C.A...... ...AC..CT. -........T 234
CV-A17.67591    .......... .......... .C.......- -...AG.... ........TA AC.A.....A ....C...T. -........T 233
EV-D70.J670-71  .......... CA........ .........- -...AG.... ........TA .C.A...... ....C...GA -......... 235
EV-A71.C08-041  .T.T...T.. .......... .........A C...GTA.CA ...G...... T.....C... ..G.A..AAA CG.T...... 237
E25.68143       GT.TT.GC.. .....A.... ..A......A CC..GTA.CA ...G...... A...T..C.. ..G.A..AA. CG.T.....T 238
CV-B4.72484     GT.....C.. .......... .AA......A CC..GTA.CA ...AG..... ....T..C.. ..G.A..AAA CG.T...... 237

                                      260                   280                   300                   320
                                        |                     |                     |                     |
MAD4            CCGCTTGTGT ACTTCGAAAA GCCTAGTATC GCCTTGGAAT CTTCGACGCG TTGCGCTCAG CACCCGACCC CGGGGTGTAG 310
CV-A13.Flores   .......... .......G.. .......... AT........ ......T... .....T.... .G.T.T.... .A.A.C.... 312
CV-A13.67900    ....CT.... .......G.. .......... AT........ ......T... .......... ...T..G... ...A...... 314
CV-A17.67591    ....CA.... .......G.. ........C. A........C .......... .......... ...T.A.A.. .A.A...... 313
EV-D70.J670-71  .....AG... .......G.. .......... AT........ ......T... .....A.... ...T.T.... .-.A...... 314
EV-A71.C08-041  ...GCCAAC. .......... ........C. A..A..A.CG AGG.AGA... ..T.....G. ...--T.... .--A...... 313
E25.68143       ...GCCAAT. .......G.. ........C. A..A..A.GG T.G..CA... ..T.....C. ...--A.... .--A...... 314
CV-B4.72484     ...GCCAAT. .......G.. A..C...... A..A..A.TG T.G..CA... ..T..T..C. ...--A.... .--A...... 313

                                      340                   360                   380                   400
                                        |                     |                     |                     |
MAD4            CTTGGGCTGA TGAGTCTGGA CATTCCTCAC CGGTGACGGT GGTCCAGGCT GCGTTGGCGG CCTACCTATG GCT-AACGCC 389
CV-A13.Flores   ...A...... .........G .....C.... ...C...... ..C....... .......... .......... ...-...... 391
CV-A13.67900    ...A...... .......... .G.C..C... ...C...... .......... .......... .......G.. ..CC..A... 394
CV-A17.67591    ...A...C.. .......... .G....C... ...C...... .......... .......... .......G.. ...C..A... 393
EV-D70.J670-71  ......TC.. .......... ..CC..A... ...C...... .......... .......... ......C... ...-.G.A.. 393
EV-A71.C08-041  A.CA...... ......ACTG ..A...C..T G..C...CA. ..CAGT.... ...C...... ...G..C... .AGA..T-.. 392
E25.68143       A.CA...... ....C.ACCG .G........ G......C.. ..CGGT.... .......... ...G..C... .GAC...-.. 393
CV-B4.72484     A.CA...C.. ......ACCG .G........ G......C.. ..CGGT.... .......... ...G..C... .GGC...-.. 392

                                      420                   440                   460                   480
                                        |                     |                     |                     |
MAD4            ATAGGACGCT A-GATGTGAA CAAGGTGTGA AGAGCCTATT GAGCTACATA AGAGTCCTCC GGCCCCTGAA TGCGGCTAAT 468
CV-A13.Flores   .....C.... .-.T...... .......... .......... ........C. .......... .......... .......... 470
CV-A13.67900    .C........ .-.T...... .......... .......... .....G..AG .......... .......... .......... 473
CV-A17.67591    .C........ .-.T..C... ..G....... .......... .........G ...T...... .......... .......... 472
EV-D70.J670-71  ..G....... .-.T...... .......C.. .......... .......C.G .......... .......... .......... 472
EV-A71.C08-041  ..G....... CTA..T.TG. ..T....C.. .......... ......GT.G GT.A...... .......... .......... 472
E25.68143       ..G....... TCA..ACTG. ..T....G.. ....T..... ......AT.G GT........ .......... .......... 473
CV-B4.72484     ..G....... TCA..ACTG. ..T....C.. ....T..... ......AT.G GT........ .......... .......... 472

                                      500                   520                   540                   560
                                        |                     |                     |                     |
MAD4            CCTAACCACG GAGCAGGCGG TCGCGAACCA GTGACTGGCT TGTCGTAACG CGCAAGTCTG TGGCGGAACC GACTACTTTG 548
CV-A13.Flores   ........T. .....A.T.A ..A.A.T... ....TATT.. .......... ........C. .......... .......... 550
CV-A13.67900    ..C....... ..A....T.. .T..A.T... .CA...A..C .......... .......... .......... .......... 553
CV-A17.67591    .......G.. .......TA. .T..A.G... .CA......C .......... ..T....... C......... .......... 552
EV-D70.J670-71  ..C....... .....AAT.C ..A.A.T... ....G...T. ........T. .......... .......... .......... 552
EV-A71.C08-041  ......TG.. ....GCA.AC C.A.A..... ...GGCA.TG C......... G....C.... CA........ .......... 552
E25.68143       ......TG.. ......ATAC C...A..... .C.GGCA.TC ........T. G....C.... CA........ .......... 553
CV-B4.72484     ......TG.. ......ATAC CTA.ACG... ..AGGCA.TC .......... G....C.... CA........ .......... 552

                                      580                   600                   620                   640
                                        |                     |                     |                     |
MAD4            GGTGTCCGTG TTTCCTGTTA TTTTTATCAT GGCTGCTTAT GGTGACAATC -AGAGATTGT TATCATAAAG CGAATTGGAT 627
CV-A13.Flores   .......... ......T... C......... .......... .......... -.TT...... .......... .......... 629
CV-A13.67900    .......... ......T... ...A.-CA.. .......... .......... -.T....... .......... .......... 631
CV-A17.67591    .......... ......T.AT ..C....A.. .......... .......... -.T....... .......... .......... 631
EV-D70.J670-71  .......... ......T... .......T.. .......... .......... -T........ .......T.. .T-....... 630
EV-A71.C08-041  .......... ......T... ...C...GT. .......... ........A. G.AGAG.... ..C....T.. .T-....... 631
E25.68143       .......... ......T..T C.C..T.AT. .......... .........T G......... .GC....T.. .T-....... 632
CV-B4.72484     .......... ......A... ....C..A.. .......... .........T G......... ..C....T.. .T-....... 631

                                      660                   680                   700                   720
                                        |                     |                     |                     |
MAD4            TGGCCATCCG GTGAGTGTTG TGTCAGGTGT ACAACTGTTT GTTGGAACCA CTGTGTTAGC TTCACTTCTC ATTCAAtCAA 707
CV-A13.Flores   .......... ....AA..GA GACTTATCA. CT.CT..... ......TT.. ..CCA..GAA CA..T.-.A. TCC..G.A-- 706
CV-A13.67900    .......... ....AA...A GA.T.AT.A. TT.C.....C ..C...TT.. ..CATC.GA. C....A-T.. ...T.GC.-- 708
CV-A17.67591    .........A ....AA..CA GACTGAT.A. C.TT...... .....GTTTG T.CCAC.CAA .....A-.A. T..GC.CT-- 708
EV-D70.J670-71  .A........ ....TATC.T GAAATTT..C CAT.ACT... TCACA..T.C TACAAC--AT .A....A.A. T...TC.TG. 708
EV-A71.C08-041  .......... ...T.CAACA GAG..AT... CT.T.A.... .....TTTTG TACCAC.GA. A.T.AAGTCT G.GATCA.TC 711
E25.68143       .......... ....CAAACA GAG..AT... TT.C..T... .....CTT.G TACC....AA ..TTAAAG.T T.AA..A.CC 712
CV-B4.72484     .......... ..ATC.AACA GAG..ATCA. TT.T...... ......TA.G TACCT...AA C.TTAAAG.T C.CAG.A.CC 711
```

S1 File: Nucleotide sequences of the 5' RNA partners

```
                                740                   760                   780                   800
MAD4           TTAATCAAAA ACAATACGAG GATAAAACAA CAATATTACA ATGGGCGCCC AAGTTTCATC ACAGAAAGTT GGAGCCCACG  787
CV-A13.Flores  -.C..TTC.. TAKTA.TC.. A..T.G...G TTG...CG.. .....T..A. .......... ...A..G... ........T.  785
CV-A13.67900   -.T..T.G.. TTGT.CT.CT A...CG.... ..T..CC... .....T..T. .G........ .......... ..T..T..T.  787
CV-A17.67591   -......G.. TTGTCTTATT ..C.CG.... T.TC..C..T .....T..T. .......G.. .........C ..T..T..T.  787
EV-D70.J670-71 A....----- ---------- --.G.G.... .----.C.T. .....A..A. .......TAG ...AC..AC. ..CA.G..T.  767
EV-A71.C08-041 ..G.ATTTGT CTTGAC.CTC A.C.C.GTC. -------.GC ......T.A. .G..G..CA. G..ACGCTCC ..CT.G..T.  784
E25.68143      .C..ATTG.T CAT.C.ACTC A..TC.G... ---------. .....G.... .G..G...A. ...A..GAC. .....G..T.  783
CV-B4.72484    .C..C.TTGT T.T.C.ATTT A.CTCGG... ---------. .....A..T. .G......A. C..A..GACA .....A....  782

                                820                   840                   860                   880
MAD4           AAAATTCAAA CAGAGCCTAC GGCGGGTCCA CCATCAATTA CACTACAATC AATTACTATA AGGACTCTGC AAGCAATGCA  867
CV-A13.Flores  .G..CA.C.. TGTT..TACT ..T.....A. .AG....... T..C..T... .....T..C. ....T..... C..T..C...  865
CV-A13.67900   ....CA.T.. TGTT...ACT ..T..T.... .AG.T..... T..G.....T ..C.....C. ....T..A.. C........T  867
CV-A17.67591   .....A.C.. ..TT...ACT ..T.....G. .TG.G..... T..G...... ........C. .......A.. T..T......  867
EV-D70.J670-71 .G...G.T.. .GTT...ACT ..A..C..A. G...A.C... ..A.CA...A ....T...C. .A..TAG.TA TGCAGCCT..  847
EV-A71.C08-041 .......T.. .TC...TACT .AA..C.... .T..A..C.. ...C..C... ..C....... .......CTA TGCTGC.A..  864
E25.68143      .G.C.GGCTT G..C...AG. ..AAAT..AG T....C.C.. ...C.AC... .....T.... ....TG.A.. TTCG...T.G  863
CV-B4.72484    .G.CCAGTCT G..T..TAGT ...AAC..T. T....C.C.. ...C.AT... ..C..T.... ....TG.... .TCG..CT.G  862

                                900                   920                   940                   960
MAD4           GCAAACAAGC AAGATTTTGC ACAAGATCCG TCCAAGTTCA CCGAACCCAT TAAGGACGTC CTTATTAAGA CCGCTCCCAT  947
CV-A13.Flores  ...TCA.... .G..C..CT. C........T ........T. ........G. A....T.... T.A..A...T .T..A..AGC  945
CV-A13.67900   ...TCA.... ........T. C..G.....A ..T..A..T. .T.....AG. G..A..T..G ..C..A..AT .A.....AGC  947
CV-A17.67591   ..CTCT..A. ........T. ...G.....C ..A....... .A.....AG. C..A..TA.. A.GT.G..AT .A......GC  947
EV-D70.J670-71 ..T.GT..A. .......CAG C.....C..A .......... .A..G..TG. AGCT..A.C. ..A.AAGCTG GA..C..AG.  927
EV-A71.C08-041 ...GG...A. .GAGCC.CAA .........A GAT......G .AA.T..TG. C..A..TA.T T.C.C.G.A. TG..GG.ACC  944
E25.68143      .......G.. .......CA. G..G..C..C AG...A.... ....G..... G..A...... A.G.....AT .ACTC..GGC  943
CV-B4.72484    .....T.G.. ........A. ...G..C..T AG........ .A.....AG. .......T.. A.G..A...T .ACTA..AGC  942

                                980                 1 000                 1 020                 1 040
MAD4           GtTAAACTCT CCAAACATTG AGGCGTGTGG TTATAGTGAC AGGGTAATGC AGCTAACTCT GGGCAATTCA ACAATCACCA 1027
CV-A13.Flores  .C.G.....C ........A. ....A..... A..C.....T ..A..T.... .AT.G..A.. T..A..C... ..C..T.... 1025
CV-A13.67900   A.....T... .......... ....T..... A......... .......... .A.....A.. C..G.....C .....A.... 1027
CV-A17.67591   AC....T..C .......... .......... G..C...... C.A..G.... .....C.AT. ...A.....C ..C..A..T. 1027
EV-D70.J670-71 CC....A..A ...TCAGCA. ....T..... .......... .....CT.A. ....C.AAT. A..A.....C .GT..AGTT. 1007
EV-A71.C08-041 AC....A... ...TCTGC.. .......... G..C..C... C.A..GGCA. ..T....CA. T........T ..C....... 1024
E25.68143      .C.G.....A ..C.CTG.G. ...A...... C..C.....T C....GCGCT CCA.C..... A..G..C..G ..C....... 1023
CV-B4.72484    .C.C..T..A ....CTG.A. ...A...... G......... .....T.GAT CAA....A.. A..A..C..G ..T..A..A. 1022

                              1 060                 1 080                 1 100                 1 120
MAD4           CCCAAGAGGC GGCCAATTCT GTGGTTGCCT ACGGTAGATG GCCTGAATAC ATCAGAGATA CCGAGGCAAA TCCTGTAGAC 1107
CV-A13.Flores  .T..G..A.. A..T..C..G .....G..T. .T..AGTG.. ....AGT... T.G..T.... AA..T..T.. C..A..T..T 1105
CV-A13.67900   .T.....A.. T........A ........T. ....AGTG.. ...CAGC..T T.G..T.... AA..T..T.. C..A..G... 1107
CV-A17.67591   .T........ A........A .......... .T...C.C.. ...AAC...T C.G.AC..C. AA..A..... ...A...... 1107
EV-D70.J670-71 .......A.. A..T..CAT. TGTTG..... .T..AGAG.. ...CACC..T C.ACCT.... AT......GT GG..A.T..T 1087
EV-A71.C08-041 .A.....A.. ...A..CAT. A.A....G.. .T...GA... ....TCC... TG.TCT...T .T..C..T.C AG.A..G..T 1104
E25.68143      .G..G..AAG T..A...GT. .......GA. .T...GTC.. ...G...... T.G......G AG.....C.C AG.ACA...T 1103
CV-B4.72484    .A.....ATG T..A...GT. .....A.GG. .....GTG.. ...C..C... C.T..T...G AA..A..G.C AG.A.A.... 1102

                              1 140                 1 160                 1 180                 1 200
MAD4           CAACCAACCG AGCCCGATGT GGCCGCGTGC AGGTTCTACA CATTAGATAC CGTCACTTGG CGCAAGGAGT CCAGAGGGTG 1187
CV-A13.Flores  .....C.... ....A..... AT.A..A..T ..A.....T. .......C.. A..AGAA... GA...A..A. ...A...A.. 1185
CV-A13.67900   ..G..C..T. .A..T..C.. ...G..A... ..A..T..T. ..C....... A..AGAA... GAG.G..... .T.A...A.. 1187
CV-A17.67591   .....C.... .A..G..... T..T..A..T C.......T. ....G..AT. A..G.TG... AAT..A.... .T.....T.. 1187
EV-D70.J670-71 A....T...C ....A..AAC AT..A.AGAT ..A..T.... .T...A.AT. .AAG.AA... GAA.GCA.CA GT.CT..... 1167
EV-A71.C08-041 A.......GC GC..A..... TT.G.T.AAT .......T.. ....G..... .AAATTA... GAG..ATC.. ...AG..A.. 1184
E25.68143      .....C..TC .A..A..C.. T..TA.C..T .....T.... .CC.G..AT. T....TG... GAG...TCC. .AGCG..C.. 1183
CV-B4.72484    .....C..TC .A..A..... C..AA.A... ..A..T.... ....GA.CT. A..G.AA... GAG.T.C.A. .AGCG..... 1182

                              1 220                 1 240                 1 260                 1 280
MAD4           GTGGTGGAAA CTACCAGACG CTTTAAAAGA CATGGGGTTA TTTGGTCAAA ACATGTTTTA TCATTATCTT GGGAGGGCTG 1267
CV-A13.Flores  .......... ..G..C..T. .A..G..... .........G .....A.... ......A... C......T.G ..C...T... 1265
CV-A13.67900   .........G ........T. ..C.C..G.. ......TC.G .....A..G. ......A... ...C..C..G .C..AT.... 1267
CV-A17.67591   .........G .......... .AC.C..... ......AC.. .....C..G. .......... C.....C... ...C.CAG.. 1267
EV-D70.J670-71 .........G ..C..T..T. ....G..TC. A..T..TA.G .......... ..G.CCAA.. C..C..C..G TAT...AG.. 1247
EV-A71.C08-041 ..AC...... T.C..G..T. TAC...C... A.CC...G.C .....C..G. .TGCACAA.T C..C..C..A TATC..T.A. 1264
E25.68143      .........G T.C.....T. .CC.TGCT.. A.....CC.. .....A.... .T.....G.. C......T.A ..A...T.A. 1263
CV-B4.72484    .........G T.C..G..T. .A...TC... G.....AC.T .....G.... .T...CAA.. ...C...... ..C..AT.G. 1262

                              1 300                 1 320                 1 340                 1 360
MAD4           GCTACACAGT GCACGTACAG TGCAATGCTT CAAAGTTTCA TCAAGGAGCT CTAGGGGTGT TTGCAGTTCC AGAAATGTGT 1347
CV-A13.Flores  .A.....C.. ...T..G... ........C. ....A..C.. ......TR.C ..T..T..A. ....T..G.. G..GTAT..C 1345
CV-A13.67900   .G........ A.....G... ........C. .T..A..C.. C..G..CA.. ..T..T..T. .......... T...TGT..C 1347
CV-A17.67591   .A..T..C.. C..T..C... .....C..C. .C........ C......A.C T.G.....A. ....CA.C.. T..GTAC... 1347
EV-D70.J670-71 .G.T.CTGTG C........A .........A ....A..... C.....TA.. ..TCTAA.AG .G..TA.C.. ....CACCA- 1326
EV-A71.C08-041 .A.TTTGCA. T.....G..A .........A GC..A..C.. .........A ..CCTA..TG C..TCC.C.. G..GTAC--- 1341
E25.68143      .T.....TA. A..T..G... ........A. .C.....C.. .......TGC ..TCTC..AG ..TGT..G.. ....GCC--- 1340
CV-B4.72484    .A..T...A. C......... ..T..C.... .T..A..C.. C.....CTGC T.GCT...TG .GTGC..A.. ....GCC--- 1339

                              1 380                 1 400                 1 420                 1 440
MAD4           TTAGCTGGTG AT---AGCAC AACTCACATG TTCACAAAGT ACGAGAATGC GAATCCAGGC GAAAAAGGAG GCGAATTCAA 1424
CV-A13.Flores  .....A.... .CTCC.ATT. G.AAAC..AC .A...T.GT. ...TS..C.. A........A ...GC...C. ..A....TGT 1425
CV-A13.67900   ........A. ..TCC.A... G.AAACA.AT .AT...TCA. .T.TC..... A..C.....T ........G. .A.TT..TGT 1427
CV-A17.67591   ..G..A..A. ..TCTGA.GT G.AAA..TCT .A...GTCA. .T.TC..C.. A........T ....GT..G. .T.TG..TGT 1427
EV-D70.J670-71 --.AT...AA .G---.AAGG ...AGGT.C. .CAG...GC. TT.CTG.A.T C.TGAA...G .CTG....T. .A.T...TG. 1401
EV-A71.C08-041 ---.T.ATC. GAACAGTGG. GGG.GGT.CA GGG...G.AG .TAGTC.CC. CCC.TACAAG C.G------- A.TC.AC.CG 1411
E25.68143      ---.AAATG. GCTCTGCGCA GCTCG...AA ACACTC..TC .TACC..ACT T.GCAACACA ..GC.C.CCA ..AC....GG 1417
CV-B4.72484    ---.AAATG. GATGC.CT.A TG..G.A.AC GCAC.C.C.. .T.GTG.CCT .TGCGGT..A ...-.C..CA AA.C.A.TTG 1415
```

S1 File: Nucleotide sequences of the 5’ RNA partners

```
                                       1 460                 1 480                 1 500                 1 520
                                           |                     |                     |                     |
MAD4           AGGAAGTTTC ACCCTTGATA CCAACGCCAC TAACCCTGCA CGGAACTTCT GCCCAGTTGA TTACCTCTTC GGGAGTGGAG 1504
CV-A13.Flores  TTCT.CC... ..T.CA..C. ..GG.---.. C.G...CAA. ..AG.G..TC AG.....G.. ......A..T ..TT....T. 1502
CV-A13.67900   .TC..C.... ..A.C...C. .ATCA---G. .C.A..A.A. ..C..A...C AA........ ....T.A... ..C....... 1504
CV-A17.67591   ..AT.AG..T ..AGC.AG.G ....G---.. A.....CA.. ..T..A..T. ..G....G.. ......AC.G ...T...... 1504
EV-D70.J670-71 .--------- ---------- ---------- C.....---- ---------- ---------- -.....T..A .ATGA...TA 1427
EV-A71.C08-041 G--------- --------CG ..G...GGTT CG.G.TACA. .ATC------ ---..TA..T GCT.GATGCT ..C.T----- 1460
E25.68143      GTC----CAT G-----A.CT ....T.AAG. .GGAG.C.TC .A..------ ---AT..A.T AC..AATGC. ..T.TG..T. 1479
CV-B4.72484    .AC.GAA.G. .-----.CC. .AGGT.AA.. .G---.C.TG .A..------ ---.G.CA.T G.G.AATGCA ..C.TG..C. 1478

                                       1 540                 1 560                 1 580                 1 600
                                           |                     |                     |                     |
MAD4           TGCTAGTAGG GAATGCGTTT GTTTATCCAC ATCAAATAAT AAACCTGCGC ACTAACAACT GTGCTACACT AGTATTGCCC 1584
CV-A13.Flores  .TA.G.C... T.....A... ....TC.... .C..G..C.. T.....C... ..A..T..T. ....C..T.. G..C...... 1582
CV-A13.67900   .T..G.CG.. T.....T..C ..A....... .C..G..C.. T..TT.AA.A ..C.....T. ....C..T.. G..GC....A 1584
CV-A17.67591   .T..C.C... C..C..T... ..G.T..... .......C.. T........G ..C....... .C..C..... T..G.....A 1584
EV-D70.J670-71 CTAGTT.G.C CTG...TC.. ..A..C..T. .C..GTGG.. ...T..TA.A ..C....... CA..A...A. T..G..A..A 1507
EV-A71.C08-041 .C.A.TATC. C..C-TAACA ..A.GC.... .C..GTGG.. ....T..A.G ..C..T..T. ....C..TA. .A..G....A 1539
E25.68143      .TGG...T.. T..CTT.ACC A.C..C..T. ....GTGG.. T.....TA.A ..C....... .C..C..CA. ....A....G 1559
CV-B4.72484    .AGG...T.. C..CCT.ACC A.A..C.... .C...TGG.. T......A.G ..C..T...A .C..C..CA. T..GA..... 1558

                                       1 620                 1 640                 1 660                 1 680
                                           |                     |                     |                     |
MAD4           TACGTGAACT CACTCTCAAT AGATAGCATG ACAAAGCACA ACAACTGGGG GATCGCTATC CTCCCCCTGG ---CGCCGCT 1661
CV-A13.Flores  .....C..T. .CT.GG.C.. T..CT.T... G......... .......... T..T.TC..A ..A..A..AT ---.CAA... 1659
CV-A13.67900   ..T..C.... .C...G.C.. ....T.T... G......... .T........ A..A.T.... T.A..A...T ---.AAAAT. 1661
CV-A17.67591   ..T.....T. ....GG.C.. C..C...... ..C..A.... .T........ A..T..A... T.A..G..CT ---.AAAA.. 1661
EV-D70.J670-71 .GGA.....A GTGCAC.... G...TTTGCT CTT.GA..T. ........AC A..A..CG.T A.T..GG.AT GTC.ATT.GC 1587
EV-A71.C08-041 ..TA.A...G ....GC.CT. T...TCTGCC TTG..C..TT G.....TT.. TC.ACTGG.T G.G...A.-- -TAGC...T. 1616
E25.68143      ..TA.A...A GTG.GC.... G....AT... TTT.G...TT ......TTAC AC.TATGG.G A.A..AT.-- -TG.A.A... 1636
CV-B4.72484    ..TA.C..TA GTG..C.... G....AT... TTC.GA.... .T..T.TTAC AC.GATG..A A.A..AT.-- -TG.A..A.. 1635

                                       1 700                 1 720                 1 740                 1 760
                                           |                     |                     |                     |
MAD4           AGATTTTGCC ACTGAATCTT CCACTGAGAT ACCCATTACA CTGACCATTG CTCCAATGTG CTGCGAATTC AATGGTTTAC 1741
CV-A13.Flores  .....A.AAT C....TG..A G....A.AT. ......C... G.T.....C. ....G..... T..T..G..T ..C.....G. 1739
CV-A13.67900   G....A.T.T C.A..C...A G...CA.AC. ...A...... A.C..G.... ....T..... ...T..G... ..C..C..GA 1741
CV-A17.67591   T..C...... T.A..CG.C. .AGTG..AT. G.....C..C G....T.... ....C..... T..T...... ..C..GC.TA 1741
EV-D70.J670-71 ..GAGG.A.T GGCA.CA.AA A...CT.TG. G..A.....C A.CT...... ....C..... TGCT....A. .....GC.TA 1667
EV-A71.C08-041 G..C....A. CAA.G.G.GA .GC.A.T... C..T.....T A.C..AC.G. .......... T.CT..G..T GCA...C.TA 1696
E25.68143      T...CAC... C.CAGCG.G. .....C.CG. T..A..A..T G....AG.C. .C..C..... TGC.....A. .....GC..A 1716
CV-B4.72484    .....A..TT ....G.G.G. ..T.CT.T.. C..A.....C G.T..GG... .C......A. .GCT....A. ..C..A..G. 1715

                                       1 780                 1 800                 1 820                 1 840
                                           |                     |                     |                     |
MAD4           GCAACATCAC TGTGCCAAGG ACCCAAGGAT TGCCAGTCTT GAACACTCCA GGGAGTAACC AGTACCTGAC CGCAGACAAT 1821
CV-A13.Flores  .G...C.G.. AA.T...GCC ........TC .C.....GA. ..G...A..T ..T....... .......... TT.T.....C 1819
CV-A13.67900   .G..TC.T.. CA.T...GC. GTA..G.... ....T..GA. ......C... ..A..C.... ....TT.... .T....T..C 1821
CV-A17.67591   .A........ CA.A..TGCA ........CC ....G..GA. ......A..C ..T.....T. .......... AT.T...... 1821
EV-D70.J670-71 .A.------- --ATG.C.TT ..A......G ....CACT.G CCTGTTG... ..CTCC.... .A.TTT.... TA.T..TG.C 1738
EV-A71.C08-041 .GC------- --AAG..GTC ..A.....G. .C..TACTGA ACTG.AA..T ..C.CG.... .A.TTT.... .A.T..TG.. 1767
E25.68143      .AC------- --.AG..G.. .AA..G..C. .A...ACAA. .CT......T ..C..C.... ...T...T.. GT.T..TG.C 1787
CV-B4.72484    .GT------- --..G.CG.. CAT.....CC .A..GAC.A. .CT...A... ..T..C.CA. .A.TTT.... AT....TG.C 1786
```
