# Supplementary material for: Evolution and Emergence of Enteroviruses through Intra- and Inter-species Recombination: Plasticity and Phenotypic Impact of Modular Genetic Exchanges in the 5’ Untranslated Region
Source: PLoS Pathog. 2015 Nov 12;11(11):e1005266. doi: 10.1371/journal.ppat.1005266 (PMC4643034; doi:10.1371/journal.ppat.1005266)
Supplement: S2 File — (DOCX) [file ppat.1005266.s013.docx]

S2 File: Nucleotidic features of group I/I recombinant genomes obtained in HEp-2c cells and L20B cells

|  | | Recombination site location | | | |  |  |
| --- | --- | --- | --- | --- | --- | --- | --- |
| Recombinant name | | 5’ partner  nt position*^a^* | Region | MAD4  nt position*^a^* | Region | Insertion/deletion length (nt)*^b^* | Mutations*^c^* |
| CV-A17/MAD4 | A.46 | 100 | Spacer 1 | 5 | CL | 96 |  |
|  | A.54 | 110 | Spacer 1 | 86 | CL | 23 |  |
|  | A.37 | 115 | Spacer 1 | 114 | Spacer 1 | H |  |
|  | A.41 | 115 | Spacer 1 | 114 | Spacer 1 | H |  |
|  | A.55 | 118 | Spacer 1 | 73 | CL | 43 |  |
|  | A.61 | 122 | Spacer 1 | 84 | CL | 32 |  |
|  | B.35 | 153 | dII | 151 | dII | H |  |
|  | B.64 | 159 | dII | 126 | dII | 31 |  |
|  | B.33 | 177 | dII-dIII | 175 | dII-dIII | H |  |
|  | B.63 | 223 | dIII-dIV | 220 | dIII-dIV | 1 |  |
|  | A.50 | 234 | dIII-dIV | 19 | CL | 213 |  |
|  | B.43 | 296 | dIV | 294 | dIV | H |  |
|  | A.56 | 453 | dV | 88 | CL | 362 |  |
|  | B.57 | 568 | dV-dVI | 565 | dV-dVI | H |  |
|  | B.53 | 570 | dV-dVI | 567 | dV-dVI | H | MAD4 568 A => U  574 A => U |
|  | B.59 | 570 | dV-dVI | 570 | dV-dVI | -3 |  |
|  | B.40 | 579 | dV-dVI | 572 | dV-dVI | 4 |  |
|  | A.44 | 580 | dV-dVI | 4 | CL | 573 |  |
|  | B.45 | 601 | dVI | 598 | dVI | H | MAD4 nt 598 to 608 were substituted by 9 U |
|  | B.60 | 603 | dVI | 600 | dVI | H |  |
|  | B.47 | 606 | dVI | 601 | dVI | 2 | MAD4 601-2 AG => UU  606 G => U |
|  | C.34 | 640 | dVII | 635 | dVII | H |  |
|  | C.39 | 647 | Spacer 2 | 743 | Spacer 2 | -99 |  |
|  | C.36 | 665 | Spacer 2 | 702 | Spacer 2 | -40 |  |
|  | C.38 | 665 | Spacer 2 | 702 | Spacer 2 | -40 |  |
|  | C.58 | 671 | Spacer 2 | 701 | Spacer 2 | -33 |  |
|  | C.51 | 689 | Spacer 2 | 684 | Spacer 2 | 7 |  |
|  | C.42 | 719 | Spacer 2 | 704 | Spacer 2 | 16 |  |
|  | C.49 | 737 | Spacer 2 | 698 | Spacer 2 | 40 |  |
|  | C.52 | 742 | Spacer 2 | 692 | Spacer 2 | 51 |  |
|  | C.62 | 745 | Spacer 2 | 691 | Spacer 2 | 55 |  |
|  | A.19 | 100 | Spacer 1 | 101 | Spacer 1 | H |  |
|  | A.14 | 101 | Spacer 1 | 89 | Spacer 1 | 12 |  |
|  | A.20 | 121 | Spacer 1 | 109 | Spacer 1 | 10 |  |
|  | A.25 | 121 | Spacer 1 | 119 | Spacer 1 | H |  |
|  | A.13 | 213 | dIII | 82 | CL | 129 |  |
|  | A.9 | 216 | dIII | 86 | CL | 128 |  |
|  | A.2b | 285 | dIV | 27 | CL | 256 |  |
|  | A.4 | 436 | dIV | 88 | CL | 345 |  |
|  | B.28 | 570 | dV-dVI | 567 | dV-dVI | H |  |
|  | B.2a | 574 | dV-dVI | 574 | dV-dVI | -3 |  |
|  | C.22 | 649 | Spacer 2 | 666 | Spacer 2 | -20 |  |
|  | C.1 | 651 | Spacer 2 | 713 | Spacer 2 | -65 |  |
|  | C.3 | 658 | Spacer 2 | 651 | Spacer 2 | 4 |  |
|  | C.7 | 661 | Spacer 2 | 739 | Spacer 2 | -81 |  |
|  | C.6 | 672 | Spacer 2 | 642 | Spacer 2 | 27 |  |
|  | C.26 | 692 | Spacer 2 | 713 | Spacer 2 | -19 |  |
|  | C.5 | 696 | Spacer 2 | 693 | Spacer 2 | -3 |  |
|  | C.10 | 697 | Spacer 2 | 638 | dVII | 61 |  |
|  | C.23 | 697 | Spacer 2 | 721 | Spacer 2 | -22 |  |
|  | C.31 | 699 | Spacer 2 | 686 | Spacer 2 | 15 |  |
|  | C.15 | 701 | Spacer 2 | 697 | Spacer 2 | 6 |  |
|  | C.18 | 704 | Spacer 2 | 711 | Spacer 2 | -6 |  |
|  | C.12 | 708 | Spacer 2 | 624 | dVII | 85 |  |
|  | C.32 | 708 | Spacer 2 | 664 | Spacer 2 | 45 |  |
|  | C.17 | 709 | Spacer 2 | 639 | dVII | 71 |  |
|  | C.8 | 726 | Spacer 2 | 653 | Spacer 2 | 74 |  |
|  | C.16 | 726 | Spacer 2 | 702 | Spacer 2 | 25 |  |
|  | C.21 | 732 | Spacer 2 | 715 | Spacer 2 | 18 |  |
|  | C.29 | 741 | Spacer 2 | 730 | Spacer 2 | 12 |  |
|  | C.11 | 744 | Spacer 2 | 590 | dVI | 155 |  |
| CV-A13.Flores/  MAD4 | A.33 | 21 | CL | 22 | CL | H |  |
|  | A.34 | 21 | CL | 22 | CL | H |  |
|  | A.62 | 116 | Spacer 1 | 45 | CL | 70 |  |
|  | A.61 | 128 | dII | 75 | CL | 52 |  |
|  | A.55 | 188 | dIII | 81 | CL | 106 |  |
|  | A.38 | 230 | dIII-dIV | 91 | Spacer 1 | 138 |  |
|  | A.59 | 244 | dIV | 38 | CL | 205 |  |
|  | B.48 | 396 | dIV | 395 | dIV | H |  |
|  | B.57 | 478 | dV | 477 | dV | H |  |
|  | B.53 | 528 | dV | 527 | dV | H |  |
|  | B.42 | 566 | dV-dVI | 565 | dV-dVI | H |  |
|  | B.56 | 566 | dV-dVI | 565 | dV-dVI | H |  |
|  | B.41 | 570 | dV-dVI | 569 | dV-dVI | H |  |
|  | B.45 | 570 | dV-dVI | 569 | dV-dVI | H |  |
|  | B.63 | 570 | dV-dVI | 569 | dV-dVI | H |  |
|  | B.47 | 596 | dVI | 595 | dVI | H | MAD4 595 A => U |
|  | B.64 | 601 | dVI | 600 | dVI | H |  |
|  | C.46 | 603 | dVI | 711 | Spacer 2 | -109 |  |
|  | C.37 | 625 | dVII | 739 | Spacer 2 | -115 |  |
|  | C.36 | 643 | Spacer 2 | 642 | Spacer 2 | H |  |
|  | C.40 | 643 | Spacer 2 | 642 | Spacer 2 | H |  |
|  | C.52 | 643 | Spacer 2 | 642 | Spacer 2 | H |  |
|  | C.50 | 669 | Spacer 2 | 710 | Spacer 2 | -42 |  |
|  | C.35 | 678 | Spacer 2 | 638 | dVII | 39 |  |
|  | C.39 | 691 | Spacer 2 | 711 | Spacer 2 | -21 |  |
|  | C.44 | 697 | Spacer 2 | 747 | Spacer 2 | -50 |  |
|  | C.54 | 700 | Spacer 2 | 706 | Spacer 2 | -6 |  |
|  | C.51 | 710 | Spacer 2 | 690 | Spacer 2 | 22 |  |
|  | C.58 | 715 | Spacer 2 | 685 | Spacer 2 | 32 |  |
|  | C.43 | 721 | Spacer 2 | 660 | Spacer 2 | 64 |  |
|  | C.60 | 734 | Spacer 2 | 610 | dVI | 127 |  |
|  | C.49 | 747 | Spacer 2 | 736 | Spacer 2 | 14 |  |
|  | A.76 | 99 | Spacer 1 | 101 | Spacer 1 | -1 |  |
|  | A.70 | 114 | Spacer 1 | 113 | Spacer 1 | H |  |
|  | B.90 | 444 | dIV-dV | 441 | dIV-dV | 2 |  |
|  | B.84 | 569 | dV-dVI | 570 | dV-dVI | -2 |  |
|  | B.72 | 569 | dV-dVI | 571 | dV-dVI | -3 | MAD4 571 U => C |
|  | B.66 | 570 | dV-dVI | 569 | dV-dVI | H |  |
|  | B.83 | 570 | dV-dVI | 569 | dV-dVI | H |  |
|  | C.68 | 602 | dVI | 731 | Spacer 2 | -130 |  |
|  | C.71 | 625 | dVII | 658 | Spacer 2 | -34 |  |
|  | C.79 | 625 | dVII | 676 | Spacer 2 | -52 |  |
|  | C.69 | 639 | dVII | 744 | Spacer 2 | -106 |  |
|  | C.67 | 647 | Spacer 2 | 733 | Spacer 2 | -87 |  |
|  | C.96 | 647 | Spacer 2 | 652 | Spacer 2 | -6 |  |
|  | C.87 | 650 | Spacer 2 | 687 | Spacer 2 | -38 |  |
|  | C.74 | 665 | Spacer 2 | 695 | Spacer 2 | -31 |  |
|  | C.93 | 666 | Spacer 2 | 686 | Spacer 2 | -21 |  |
|  | C.86 | 679 | Spacer 2 | 703 | Spacer 2 | -25 |  |
|  | C.94 | 679 | Spacer 2 | 686 | Spacer 2 | -8 |  |
|  | C.89 | 686 | Spacer 2 | 740 | Spacer 2 | -55 |  |
|  | C.77 | 697 | Spacer 2 | 703 | Spacer 2 | -2 |  |
|  | C.92 | 698 | Spacer 2 | 723 | Spacer 2 | -25 |  |
|  | C.85 | 710 | Spacer 2 | 746 | Spacer 2 | -34 |  |
|  | C.75 | 712 | Spacer 2 | 656 | Spacer 2 | 58 |  |
|  | C.73 | 713 | Spacer 2 | 565 | dV-dVI | 150 |  |
|  | C.95 | 713 | Spacer 2 | 647 | Spacer 2 | 68 |  |
|  | C.81 | 714 | Spacer 2 | 666 | Spacer 2 | 50 | Flores 633 C => U |
|  | C.88 | 715 | Spacer 2 | 713 | Spacer 2 | 4 |  |
|  | C.65 | 722 | Spacer 2 | 623 | dVII | 102 |  |
|  | C.82 | 730 | Spacer 2 | 703 | Spacer 2 | 30 |  |
|  | C.80 | 738 | Spacer 2 | 719 | Spacer 2 | 22 |  |
|  | C.78 | 740 | Spacer 2 | 710 | Spacer 2 | 33 |  |
|  | C.91 | 742 | Spacer 2 | 597 | dVI | 148 |  |
| CV-A13.67900/  MAD4 | A.56 | 96 | Spacer 1 | 98 | Spacer 1 | -1 |  |
|  | A.61 | 104 | Spacer 1 | 104 | Spacer 1 | -2 |  |
|  | A.72 | 119 | Spacer 1 | 82 | CL | 35 |  |
|  | A.51 | 125 | Spacer 1 | 77 | CL | 45 |  |
|  | B.63 | 143 | dII | 139 | dII | 1 |  |
|  | B.69 | 145 | dII | 142 | dII | H | MAD4 nt 142 to 145 were substituted by AAUU |
|  | A.71 | 162 | dII | 82 | CL | 77 |  |
|  | B.58 | 194 | dIII | 191 | dIII | H |  |
|  | A.64 | 228 | dIII-dIV | 41 | CL | 184 |  |
|  | A.46 | 360 | dIV | 84 | CL | 273 |  |
|  | B.45 | 517 | dV | 513 | dV | H | MAD4 513 G => A |
|  | B.60 | 569 | dV-dVI | 565 | dV-dVI | H |  |
|  | B.67 | 571 | dV-dVI | 568 | dV-dVI | -1 |  |
|  | B.48 | 572 | dV-dVI | 568 | dV-dVI | H | MAD4 568 A => U |
|  | A.54 | 572 | dV-dVI | 24 | CL | 544 |  |
|  | B.44 | 576 | dV-dVI | 572 | dV-dVI | H |  |
|  | B.49 | 576 | dV-dVI | 572 | dV-dVI | H |  |
|  | B.50 | 576 | dV-dVI | 572 | dV-dVI | H |  |
|  | B.57 | 576 | dV-dVI | 572 | dV-dVI | H |  |
|  | B.59 | 576 | dV-dVI | 572 | dV-dVI | H |  |
|  | B.62 | 576 | dV-dVI | 572 | dV-dVI | H |  |
|  | B.65 | 576 | dV-dVI | 572 | dV-dVI | H |  |
|  | B.68 | 576 | dV-dVI | 572 | dV-dVI | H |  |
|  | B.74 | 576 | dV-dVI | 572 | dV-dVI | H |  |
|  | B.53 | 603 | dVI | 600 | dVI | H |  |
|  | B.55 | 605 | dVI | 602 | dVI | H | MAD4 602-3 GA => AU |
|  | B.52 | 611 | dVI | 608 | dVI | H | MAD4 608-9 UA => AU |
|  | C.47 | 627 | dVII | 731 | Spacer 2 | -107 |  |
|  | C.73 | 694 | Spacer 2 | 600 | dVI | 91 |  |
|  | C.70 | 706 | Spacer 2 | 706 | Spacer 2 | -2 |  |
|  | C.66 | 714 | Spacer 2 | 659 | Spacer 2 | 56 |  |
|  | C.43 | 779 | VP4 | 780 | VP4 | H |  |
|  | A.5 | 29 | CL | 30 | CL | H | MAD4 30 C => U |
|  | A.19 | 97 | Spacer 1 | 99 | Spacer 1 | -1 |  |
|  | A.17 | 104 | Spacer 1 | 104 | Spacer 1 | -2 |  |
|  | A.32 | 117 | Spacer 1 | 40 | CL | 75 |  |
|  | A.20 | 118 | Spacer 1 | 58 | CL | 58 |  |
|  | A.7 | 127 | Spacer 1 | 5 | CL | 119 |  |
|  | A.28 | 134 | dII | 20 | CL | 111 |  |
|  | A.3 | 208 | dIII | 32 | CL | 173 |  |
|  | A.6 | 371 | dIV | 87 | CL | 281 |  |
|  | B.9 | 470 | dV | 465 | dV | 1 |  |
|  | B.12 | 475 | dV | 471 | dV | H | 67900 28 C => U |
|  | B.24 | 604 | dVI | 578 | dV-dVI | 23 |  |
|  | B.30 | 612 | dVI | 609 | dVI | H | MAD4 609 A => U |
|  | C.15 | 622 | dVI | 702 | Spacer 2 | -83 |  |
|  | C.27 | 657 | Spacer 2 | 710 | Spacer 2 | -56 |  |
|  | C.21 | 662 | Spacer 2 | 707 | Spacer 2 | -48 |  |
|  | C.10 | 680 | Spacer 2 | 688 | Spacer 2 | -11 |  |
|  | C.4 | 684 | Spacer 2 | 638 | dVII | 43 |  |
|  | C.31 | 694 | Spacer 2 | 700 | Spacer 2 | -9 |  |
|  | C.23 | 699 | Spacer 2 | 664 | Spacer 2 | 33 |  |
|  | C.2 | 710 | Spacer 2 | 643 | Spacer 2 | 68 |  |
|  | C.8 | 710 | Spacer 2 | 666 | Spacer 2 | 45 |  |
|  | C.13 | 710 | Spacer 2 | 690 | Spacer 2 | 21 |  |
|  | C.26 | 714 | Spacer 2 | 713 | Spacer 2 | 2 |  |
|  | C.14 | 718 | Spacer 2 | 719 | Spacer 2 | H |  |
|  | C.25 | 732 | Spacer 2 | 745 | Spacer 2 | -12 |  |
|  | C.18 | 737 | Spacer 2 | 718 | Spacer 2 | 20 |  |
|  | C.22 | 740 | Spacer 2 | 710 | Spacer 2 | 31 |  |
|  | C.29 | 740 | Spacer 2 | 620 | dVII | 121 |  |
|  | C.1 | 748 | Spacer 2 | 710 | Spacer 2 | 39 |  |
| EV-D70/MAD4 | A.49 | 100 | Spacer 1 | 101 | Spacer 1 | H |  |
|  | A.60 | 101 | Spacer 1 | 102 | Spacer 1 | -1 |  |
|  | A.42 | 215 | dIII | 87 | CL | 124 |  |
|  | B.43 | 294 | dIV | 287 | dIV | 3 |  |
|  | A.64 | 487 | dV | 115 | Spacer 1 | 369 |  |
|  | A.52 | 527 | dV | 80 | CL | 444 |  |
|  | B.45 | 566 | dV-dVI | 563 | dV-dVI | H | MAD4 563 U => C |
|  | B.46 | 566 | dV-dVI | 563 | dV-dVI | H | MAD4 563 U => C |
|  | B.41 | 568 | dV-dVI | 565 | dV-dVI | H |  |
|  | B.44 | 571 | dV-dVI | 572 | dV-dVI | -4 |  |
|  | B.56 | 571 | dV-dVI | 569 | dV-dVI | -1 |  |
|  | B.58 | 571 | dV-dVI | 568 | dV-dVI | H | MAD4 568 A => U |
|  | B.36 | 579 | dV-dVI | 576 | dV-dVI | H |  |
|  | B.39 | 579 | dV-dVI | 576 | dV-dVI | H |  |
|  | B.40 | 579 | dV-dVI | 576 | dV-dVI | H |  |
|  | B.48 | 579 | dV-dVI | 576 | dV-dVI | H |  |
|  | B.53 | 579 | dV-dVI | 576 | dV-dVI | H |  |
|  | B.61 | 579 | dV-dVI | 576 | dV-dVI | H |  |
|  | C.57 | 595 | dVI | 714 | Spacer 2 | -122 |  |
|  | B.38 | 597 | dVI | 594 | dVI | H | MAD4 nt 594 to 599 were substituted by 6 U |
|  | C.33 | 682 | Spacer 2 | 743 | Spacer 2 | -65 |  |
|  | C.34 | 697 | Spacer 2 | 684 | Spacer 2 | 13 |  |
|  | C.47 | 702 | Spacer 2 | 728 | Spacer 2 | -26 |  |
|  | C.62 | 702 | Spacer 2 | 714 | Spacer 2 | -12 |  |
|  | C.50 | 703 | Spacer 2 | 551 | dV | 152 |  |
|  | C.35 | 704 | Spacer 2 | 706 | Spacer 2 | -2 |  |
|  | C.54 | 709 | Spacer 2 | 698 | Spacer 2 | 11 |  |
|  | C.51 | 719 | Spacer 2 | 566 | dV-dVI | 170 |  |
|  | C.37 | 720 | Spacer 2 | 680 | Spacer 2 | 57 |  |
|  | A.16 | 95 | Spacer 1 | 96 | Spacer 1 | H |  |
|  | A.22 | 96 | Spacer 1 | 98 | Spacer 1 | -1 |  |
|  | A.7 | 101 | Spacer 1 | 101 | Spacer 1 | H |  |
|  | A.24 | 102 | Spacer 1 | 102 | Spacer 1 | -1 |  |
|  | A.25 | 102 | Spacer 1 | 102 | Spacer 1 | -1 |  |
|  | A.27 | 102 | Spacer 1 | 102 | Spacer 1 | -1 |  |
|  | A.15 | 110 | Spacer 1 | 108 | Spacer 1 | H |  |
|  | A.28 | 199 | dIII | 91 | Spacer 1 | 104 |  |
|  | B.13 | 241 | dIV | 237 | dIV | H |  |
|  | B.8 | 336 | dIV | 333 | dIV | H |  |
|  | B.6 | 579 | dV-dVI | 576 | dV-dVI | H |  |
|  | B.9 | 579 | dV-dVI | 576 | dV-dVI | H |  |
|  | C.1 | 581 | dV-dVI | 742 | Spacer 2 | -164 |  |
|  | C.11 | 625 | dVII | 651 | Spacer 2 | -28 |  |
|  | C.14 | 643 | dVII | 618 | dVI | 23 |  |
|  | C.29 | 657 | Spacer 2 | 617 | dVI | 37 |  |
|  | C.32 | 659 | Spacer 2 | 669 | Spacer 2 | -13 |  |
|  | C.2 | 671 | Spacer 2 | 732 | Spacer 2 | -64 |  |
|  | C.20 | 673 | Spacer 2 | 602 | dVI | 68 |  |
|  | C.18 | 677 | Spacer 2 | 701 | Spacer 2 | -27 |  |
|  | C.21 | 681 | Spacer 2 | 690 | Spacer 2 | -13 |  |
|  | C.4 | 690 | Spacer 2 | 714 | Spacer 2 | -28 |  |
|  | C.23 | 701 | Spacer 2 | 657 | Spacer 2 | 44 |  |
|  | C.31 | 713 | Spacer 2 | 718 | Spacer 2 | -5 |  |
|  | C.12 | 714 | Spacer 2 | 689 | Spacer 2 | 42 |  |
|  | C.10 | 718 | Spacer 2 | 621 | dVII | 114 |  |
|  | C.30 | 724 | Spacer 2 | 636 | dVII | 109 |  |
|  | C.17 | 729 | Spacer 2 | 705 | Spacer 2 | 45 |  |

*^a^* Numbering refers to the nucleotide sequence of the indicated 5’ partner and of MAD4, respectively.

*^b^* Homologous recombination sites (H) display neither an insertion nor a deletion according to aligned parental sequences. The insertion (+) or deletion (-) of nt in nonhomologous sites is indicated.

*^c^* Additional mutations compared to parental strains (about 1000 nt at the 5’ end of the viral RNA were sequenced).
