# Supplementary material for: Evolution and Emergence of Enteroviruses through Intra- and Inter-species Recombination: Plasticity and Phenotypic Impact of Modular Genetic Exchanges in the 5’ Untranslated Region
Source: PLoS Pathog. 2015 Nov 12;11(11):e1005266. doi: 10.1371/journal.ppat.1005266 (PMC4643034; doi:10.1371/journal.ppat.1005266)
Supplement: S3 File — (DOCX) [file ppat.1005266.s014.docx]

S3 File: Nucleotidic features of group II/I recombinant genomes obtained in HEp-2c cells and L20B cells

|  | | Recombination site location | | | |  |  |
| --- | --- | --- | --- | --- | --- | --- | --- |
| Recombinant name | | 5’ partner  nt position*^a^* | Region | MAD4  nt position*^a^* | Region | Insertion/deletion length (nt)*^b^* | Mutations*^c^* |
| CV-B4/MAD4 | A.53 | 100 | Spacer 1 | 102 | Spacer 1 | H |  |
|  | A.61 | 101 | Spacer 1 | 103 | Spacer 1 | H |  |
|  | A.40 | 110 | Spacer 1 | 75 | CL | 33 |  |
|  | A.45 | 111 | Spacer 1 | 104 | Spacer 1 | 5 |  |
|  | A.50 | 114 | Spacer 1 | 27 | CL | 85 | MAD4 200 C => A |
|  | A.44 | 115 | Spacer 1 | 87 | CL | 26 |  |
|  | A.56 | 130 | dII | 2 | CL | 125 |  |
|  | A.52 | 132 | dII | 71 | CL | 58 |  |
|  | A.34 | 178 | dII-dIII | 86 | CL | 89 |  |
|  | A.43 | 179 | dII-dIII | 23 | CL | 153 |  |
|  | B.49 | 183 | dII-dIII | 130 | dII | 50 |  |
|  | A.33 | 195 | dIII | 75 | CL | 115 |  |
|  | A.62 | 196 | dIII | 28 | CL | 163 |  |
|  | A.37 | 209 | dIII | 51 | CL | 153 |  |
|  | A.36 | 210 | dIII | 54 | CL | 151 |  |
|  | A.46 | 233 | dIII-dIV | 80 | CL | 147 |  |
|  | B.38 | 240 | dIII-dIV | 234 | dIV | H |  |
|  | A.58 | 302 | dIV | 111 | Spacer 1 | 187 |  |
|  | C.39 | 621 | dVI | 659 | Spacer 2 | -42 |  |
|  | C.59 | 628 | dVII | 738 | Spacer 2 | -113 |  |
|  | C.48 | 645 | Spacer 2 | 706 | Spacer 2 | -64 |  |
|  | C.35 | 647 | Spacer 2 | 689 | Spacer 2 | -45 |  |
|  | C.63 | 671 | Spacer 2 | 684 | Spacer 2 | -16 |  |
|  | C.64 | 679 | Spacer 2 | 748 | Spacer 2 | -72 |  |
|  | C.54 | 690 | Spacer 2 | 659 | Spacer 2 | 28 |  |
|  | C.42 | 693 | Spacer 2 | 733 | Spacer 2 | -43 |  |
|  | C.57 | 694 | Spacer 2 | 720 | Spacer 2 | -29 |  |
|  | C.60 | 694 | Spacer 2 | 685 | Spacer 2 | 6 |  |
|  | C.51 | 719 | Spacer 2 | 575 | dV-dVI | 141 |  |
|  | C.47 | 722 | Spacer 2 | 700 | Spacer 2 | 19 |  |
|  | C.55 | 725 | Spacer 2 | 684 | Spacer 2 | 38 |  |
|  | C.41 | 744 | Spacer 2 | 745 | Spacer 2 | 5 |  |
|  | A.24 | 103 | Spacer 1 | 104 | Spacer 1 | -3 |  |
|  | A.19 | 192 | dIII | 4 | CL | 183 |  |
|  | A.15 | 200 | dIII | 75 | CL | 120 |  |
|  | A.26 | 265 | dIV | 58 | CL | 201 |  |
|  | C.12 | 620 | dVI | 670 | Spacer 2 | -54 |  |
|  | C.31 | 640 | dVII | 591 | dVI | 46 |  |
|  | C.11 | 644 | Spacer 2 | 737 | Spacer 2 | -96 |  |
|  | C.7 | 646 | Spacer 2 | 660 | Spacer 2 | -17 |  |
|  | C.28 | 651 | Spacer 2 | 652 | Spacer 2 | -4 |  |
|  | C.14 | 658 | Spacer 2 | 669 | Spacer 2 | -14 |  |
|  | C.18 | 658 | Spacer 2 | 650 | Spacer 2 | 5 |  |
|  | C.3 | 659 | Spacer 2 | 600 | dVI | 56 |  |
|  | C.2 | 660 | Spacer 2 | 711 | Spacer 2 | -54 |  |
|  | C.13 | 662 | Spacer 2 | 695 | Spacer 2 | -36 |  |
|  | C.25 | 662 | Spacer 2 | 685 | Spacer 2 | -28 |  |
|  | C.16 | 671 | Spacer 2 | 705 | Spacer 2 | -37 |  |
|  | C.4 | 674 | Spacer 2 | 710 | Spacer 2 | -39 |  |
|  | C.30 | 674 | Spacer 2 | 744 | Spacer 2 | -73 |  |
|  | C.5 | 688 | Spacer 2 | 695 | Spacer 2 | -10 |  |
|  | C.6 | 694 | Spacer 2 | 685 | Spacer 2 | 6 |  |
|  | C.22 | 700 | Spacer 2 | 695 | Spacer 2 | 2 |  |
|  | C.23 | 700 | Spacer 2 | 701 | Spacer 2 | -8 |  |
|  | C.20 | 701 | Spacer 2 | 731 | Spacer 2 | -33 |  |
|  | C.29 | 702 | Spacer 2 | 719 | Spacer 2 | -20 |  |
|  | C.1 | 705 | Spacer 2 | 709 | Spacer 2 | -7 |  |
|  | C.21 | 705 | Spacer 2 | 699 | Spacer 2 | 3 |  |
|  | C.27 | 713 | Spacer 2 | 636 | dVII | 74 | MAD4 658 A => G ;  661 A => G ; 685 A => G |
|  | C.8 | 726 | Spacer 2 | 663 | Spacer 2 | 60 |  |
|  | C.10 | 726 | Spacer 2 | 663 | Spacer 2 | 60 |  |
|  | C.9 | 730 | Spacer 2 | 710 | Spacer 2 | 20 |  |
|  | C.17 | 730 | Spacer 2 | 745 | Spacer 2 | -15 |  |
|  | C.32 | 739 | Spacer 2 | 579 | dV-dVI | 160 |  |
| E25/MAD4 | A.54 | 100 | Spacer 1 | 102 | Spacer 1 | -1 |  |
|  | A.38 | 101 | Spacer 1 | 40 | CL | 61 |  |
|  | A.34 | 107 | Spacer 1 | 3 | CL | 101 |  |
|  | A.50 | 107 | Spacer 1 | 40 | CL | 64 |  |
|  | A.39 | 115 | Spacer 1 | 82 | CL | 42 | nt 1 to 115 from E25, then insertion of E25 nt 102 to 113, then nt 82 to the end from MAD4 |
|  | A.60 | 122 | Spacer 1 | 3 | CL | 116 |  |
|  | A.42 | 128 | Spacer 1 | 107 | Spacer 1 | 17 |  |
|  | A.53 | 151 | dII | 107 | Spacer 1 | 40 |  |
|  | B.52 | 237 | dIII-dIV | 230 | dIII-dIV | H |  |
|  | A.48 | 251 | dIV | 118 | Spacer 1 | 126 |  |
|  | B.56 | 567 | dV-dVI | 563 | dV-dVI | H | MAD4 563 C => U |
|  | B.43 | 569 | dV-dVI | 565 | dV-dVI | H |  |
|  | B.58 | 572 | dV-dVI | 568 | dV-dVI | H |  |
|  | B.55 | 578 | dV-dVI | 568 | dV-dVI | 6 |  |
|  | C.49 | 606 | dVI | 699 | Spacer 2 | -98 |  |
|  | C.41 | 640 | dVII | 700 | Spacer 2 | -64 |  |
|  | C.44 | 640 | dVII | 737 | Spacer 2 | -101 |  |
|  | C.63 | 649 | Spacer 2 | 749 | Spacer 2 | -104 |  |
|  | C.61 | 664 | Spacer 2 | 705 | Spacer 2 | -45 |  |
|  | C.37 | 670 | Spacer 2 | 625 | dVII | 41 |  |
|  | C.40 | 680 | Spacer 2 | 726 | Spacer 2 | -50 |  |
|  | C.46 | 691 | Spacer 2 | 649 | Spacer 2 | 38 |  |
|  | C.47 | 691 | Spacer 2 | 649 | Spacer 2 | 38 |  |
|  | C.62 | 693 | Spacer 2 | 742 | Spacer 2 | -53 |  |
|  | C.59 | 694 | Spacer 2 | 723 | Spacer 2 | -33 |  |
|  | C.36 | 698 | Spacer 2 | 610 | dVI | 97 |  |
|  | C.35 | 704 | Spacer 2 | 697 | Spacer 2 | 16 |  |
|  | C.33 | 732 | Spacer 2 | 727 | Spacer 2 | 14 |  |
|  | C.45 | 734 | Spacer 2 | 733 | Spacer 2 | 10 |  |
|  | C.64 | 738 | Spacer 2 | 687 | Spacer 2 | 58 |  |
|  | C.57 | 745 | VP4 | 690 | Spacer 2 | 60 |  |
|  | A.31 | 114 | Spacer 1 | 24 | CL | 87 |  |
|  | A.18 | 115 | Spacer 1 | 2 | CL | 110 |  |
|  | A.6 | 117 | Spacer 1 | 114 | Spacer 1 | H |  |
|  | A.22 | 117 | Spacer 1 | 114 | Spacer 1 | H |  |
|  | A.29 | 165 | dII | 4 | CL | 157 |  |
|  | A.32 | 256 | dIV | 36 | CL | 213 |  |
|  | A.16 | 257 | dIV | 3 | CL | 247 |  |
|  | C.10 | 632 | dVII | 710 | Spacer 2 | -82 |  |
|  | C.11 | 645 | Spacer 2 | 657 | Spacer 2 | -16 |  |
|  | C.3 | 655 | Spacer 2 | 652 | Spacer 2 | -1 |  |
|  | C.30 | 664 | Spacer 2 | 649 | Spacer 2 | 11 |  |
|  | C.28 | 665 | Spacer 2 | 660 | Spacer 2 | 1 |  |
|  | C.21 | 687 | Spacer 2 | 738 | Spacer 2 | -55 |  |
|  | C.2 | 694 | Spacer 2 | 701 | Spacer 2 | -11 |  |
|  | C.26 | 696 | Spacer 2 | 635 | dVII | 70 |  |
|  | C.20 | 701 | Spacer 2 | 685 | Spacer 2 | 25 |  |
|  | C.14 | 708 | Spacer 2 | 665 | Spacer 2 | 52 |  |
|  | C.12 | 709 | Spacer 2 | 712 | Spacer 2 | 54 | Insertion of a nontemplate sequence of 48 nt : ACCGUGAUUGAUUGAUUGAUUGAUUGAUUGAUUUUGGUCUUCGGUUUU |
|  | C.19 | 709 | Spacer 2 | 718 | Spacer 2 | H |  |
|  | C.17 | 711 | Spacer 2 | 669 | Spacer 2 | 51 |  |
|  | C.4 | 715 | Spacer 2 | 622 | dVII | 102 |  |
|  | C.1 | 726 | Spacer 2 | 716 | Spacer 2 | 19 |  |
|  | C.27 | 728 | Spacer 2 | 692 | Spacer 2 | 45 |  |
|  | C.7 | 733 | Spacer 2 | 659 | Spacer 2 | 83 | Insertion of an A between E25 nt 709 and 710 |
|  | C.23 | 733 | Spacer 2 | 675 | Spacer 2 | 67 |  |
|  | C.13 | 735 | Spacer 2 | 614 | dVI | 130 | Insertion of an A between E25 nt 709 and 710 |
|  | C.8 | 740 | Spacer 2 | 695 | Spacer 2 | 50 |  |
|  | C.24 | 742 | Spacer 2 | 686 | Spacer 2 | 61 |  |
|  | C.25 | 742 | Spacer 2 | 743 | Spacer 2 | 4 |  |
|  | C.9 | 743 | Spacer 2 | 676 | Spacer 2 | 66 |  |
|  | C.15 | 745 | Spacer 2 | 688 | Spacer 2 | 62 |  |
| EV-A71/MAD4 | A.57 | 107 | Spacer 1 | 20 | CL | 85 |  |
|  | A.33 | 112 | Spacer 1 | 27 | CL | 83 |  |
|  | A.35 | 112 | Spacer 1 | 4 | CL | 106 |  |
|  | A.36 | 112 | Spacer 1 | 4 | CL | 106 |  |
|  | B.45 | 571 | dV-dVI | 569 | dV-dVI | -1 |  |
|  | B.55 | 571 | dV-dVI | 572 | dV-dVI | -4 |  |
|  | C.58 | 605 | dVI | 675 | Spacer 2 | -73 |  |
|  | C.51 | 627 | dVII | 693 | Spacer 2 | -69 |  |
|  | C.38 | 632 | dVII | 731 | Spacer 2 | -102 |  |
|  | C.39 | 632 | dVII | 731 | Spacer 2 | -102 |  |
|  | C.59 | 639 | dVII | 720 | Spacer 2 | -84 |  |
|  | C.41 | 642 | dVII | 701 | Spacer 2 | -62 |  |
|  | C.48 | 645 | Spacer 2 | 697 | Spacer 2 | -55 |  |
|  | C.49 | 645 | Spacer 2 | 697 | Spacer 2 | -55 |  |
|  | C.40 | 646 | Spacer 2 | 601 | dVI | 42 |  |
|  | C.37 | 651 | Spacer 2 | 645 | Spacer 2 | 3 |  |
|  | C.50 | 656 | Spacer 2 | 659 | Spacer 2 | -6 |  |
|  | C.47 | 665 | Spacer 2 | 650 | Spacer 2 | 12 |  |
|  | C.56 | 673 | Spacer 2 | 686 | Spacer 2 | -16 |  |
|  | C.60 | 688 | Spacer 2 | 614 | dVI | 66 |  |
|  | C.46 | 695 | Spacer 2 | 722 | Spacer 2 | -36 |  |
|  | C.42 | 697 | Spacer 2 | 685 | Spacer 2 | 3 |  |
|  | C.44 | 699 | Spacer 2 | 570 | dV-dVI | 120 |  |
|  | C.54 | 699 | Spacer 2 | 741 | Spacer 2 | -51 |  |
|  | C.43 | 721 | Spacer 2 | 640 | dVII | 73 |  |
|  | C.34 | 752 | VP4 | 756 | VP4 | H |  |
|  | A.24 | 95 | Spacer 1 | 20 | CL | 77 |  |
|  | A.17 | 117 | Spacer 1 | 114 | Spacer 1 | 1 |  |
|  | A.12 | 149 | dII | 3 | CL | 143 |  |
|  | B.14 | 571 | dV-VI | 570 | dV-VI | -2 |  |
|  | C.30 | 613 | dVI | 671 | Spacer 2 | -62 | EV-A71 525 C => A |
|  | C.28 | 621 | dVI | 689 | Spacer 2 | -72 |  |
|  | C.13 | 627 | dVII | 713 | Spacer 2 | -89 |  |
|  | C.32 | 632 | dVII | 745 | Spacer 2 | -116 |  |
|  | C.6 | 635 | dVII | 619 | dVII | 13 |  |
|  | C.15 | 649 | Spacer 2 | 708 | Spacer 2 | -62 |  |
|  | C.5 | 658 | Spacer 2 | 710 | Spacer 2 | -55 |  |
|  | C.2 | 664 | Spacer 2 | 711 | Spacer 2 | -50 |  |
|  | C.7 | 666 | Spacer 2 | 696 | Spacer 2 | -33 |  |
|  | C.20 | 667 | Spacer 2 | 621 | dVII | 43 |  |
|  | C.9 | 672 | Spacer 2 | 745 | Spacer 2 | -76 |  |
|  | C.1 | 680 | Spacer 2 | 699 | Spacer 2 | -25 |  |
|  | C.10 | 682 | Spacer 2 | 695 | Spacer 2 | -21 |  |
|  | C.8 | 693 | Spacer 2 | 702 | Spacer 2 | -18 | Insertion of a U between  EV-A71 nt 575 and 576 |
|  | C.11 | 713 | Spacer 2 | 710 | Spacer 2 | -5 |  |
|  | C.29 | 721 | Spacer 2 | 721 | Spacer 2 | -8 |  |
|  | C.27 | 728 | Spacer 2 | 578 | dV-VI | 142 |  |
|  | C.19 | 733 | Spacer 2 | 616 | dVI | 109 |  |
|  | C.22 | 735 | Spacer 2 | 704 | Spacer 2 | 23 |  |
|  | C.23 | 735 | Spacer 2 | 707 | Spacer 2 | 20 |  |
|  | C.16 | 736 | Spacer 2 | 665 | Spacer 2 | 63 |  |
|  | C.21 | 736 | Spacer 2 | 668 | Spacer 2 | 60 |  |
|  | C.25 | 737 | Spacer 2 | 703 | Spacer 2 | 26 |  |
|  | C.26 | 737 | Spacer 2 | 669 | Spacer 2 | 60 |  |
|  | C.31 | 737 | Spacer 2 | 721 | Spacer 2 | 8 |  |
|  | C.4 | 744 | Spacer 2 | 688 | Spacer 2 | 48 |  |

*^a^* Numbering refers to the nucleotide sequence of the indicated 5’ partner and of MAD4, respectively.

*^b^* Homologous recombination sites (H) display neither an insertion nor a deletion according to aligned parental sequences. The insertion (+) or deletion (-) of nt in nonhomologous sites is indicated.

*^c^* Additional mutations compared to parental strains (about 1000 nt at the 5’ end of the viral RNA were sequenced).
